# Supplementary material for: Development of a Joint-Specialty Simulation-Based Workshop to Optimize Counseling at Extreme Prematurity
Source: MedEdPORTAL. 2026 Jul 29;22:11623. doi: 10.15766/mep_2374-8265.11623 (PMC13415433; doi:10.15766/mep_2374-8265.11623)
Supplement: Supplementary file 1 — Prenatal Counseling Workshop.pptxPrenatal Counseling Case.docxFacilitator Guide.docxPostworkshop Survey.docx [file mep_2374-8265.11623-s001.zip › A. Prenatal Counseling Workshop.pptx]

## Slide 1
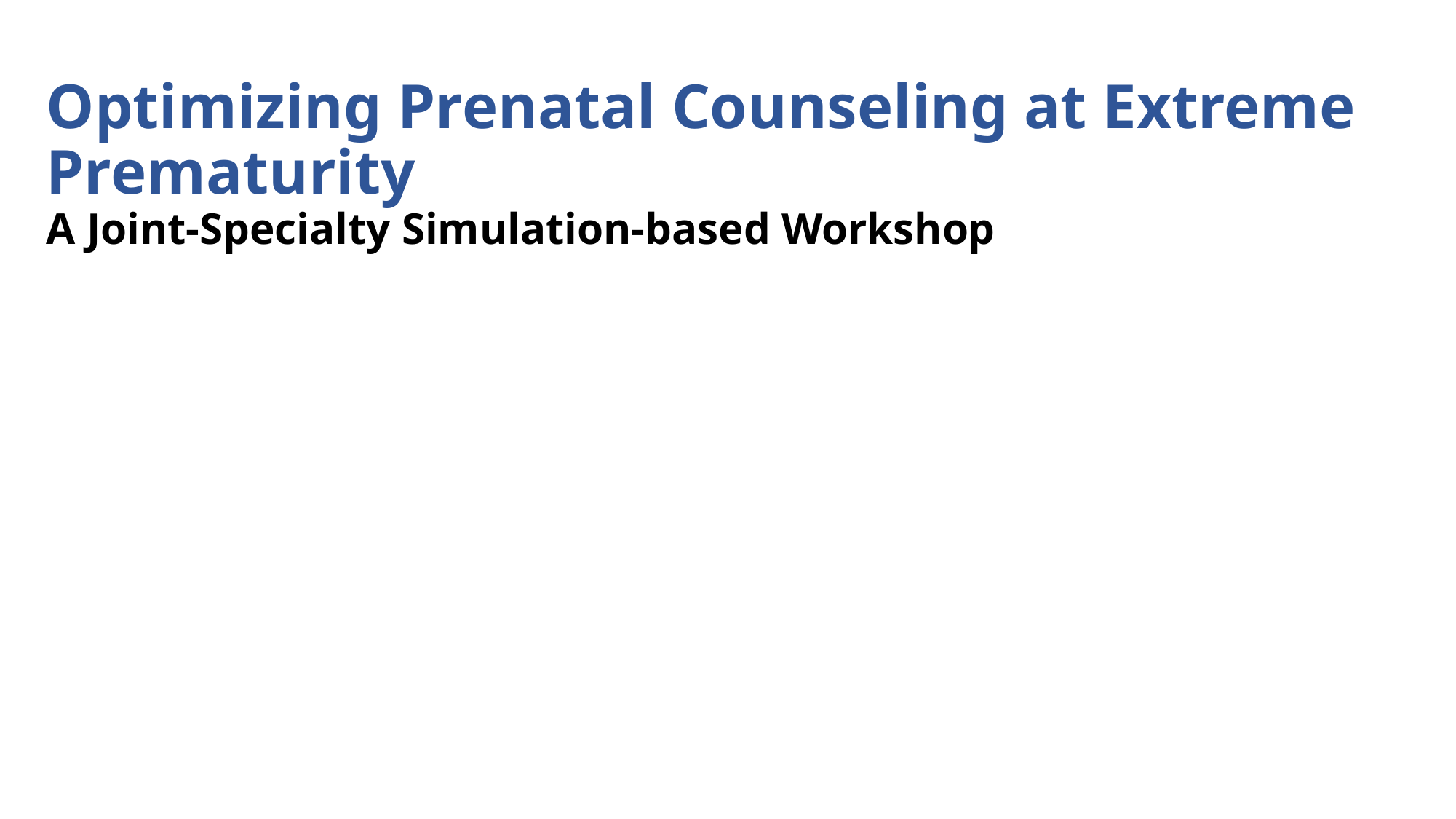

# Optimizing Prenatal Counseling at Extreme Prematurity A Joint-Specialty Simulation-based Workshop

## Slide 2
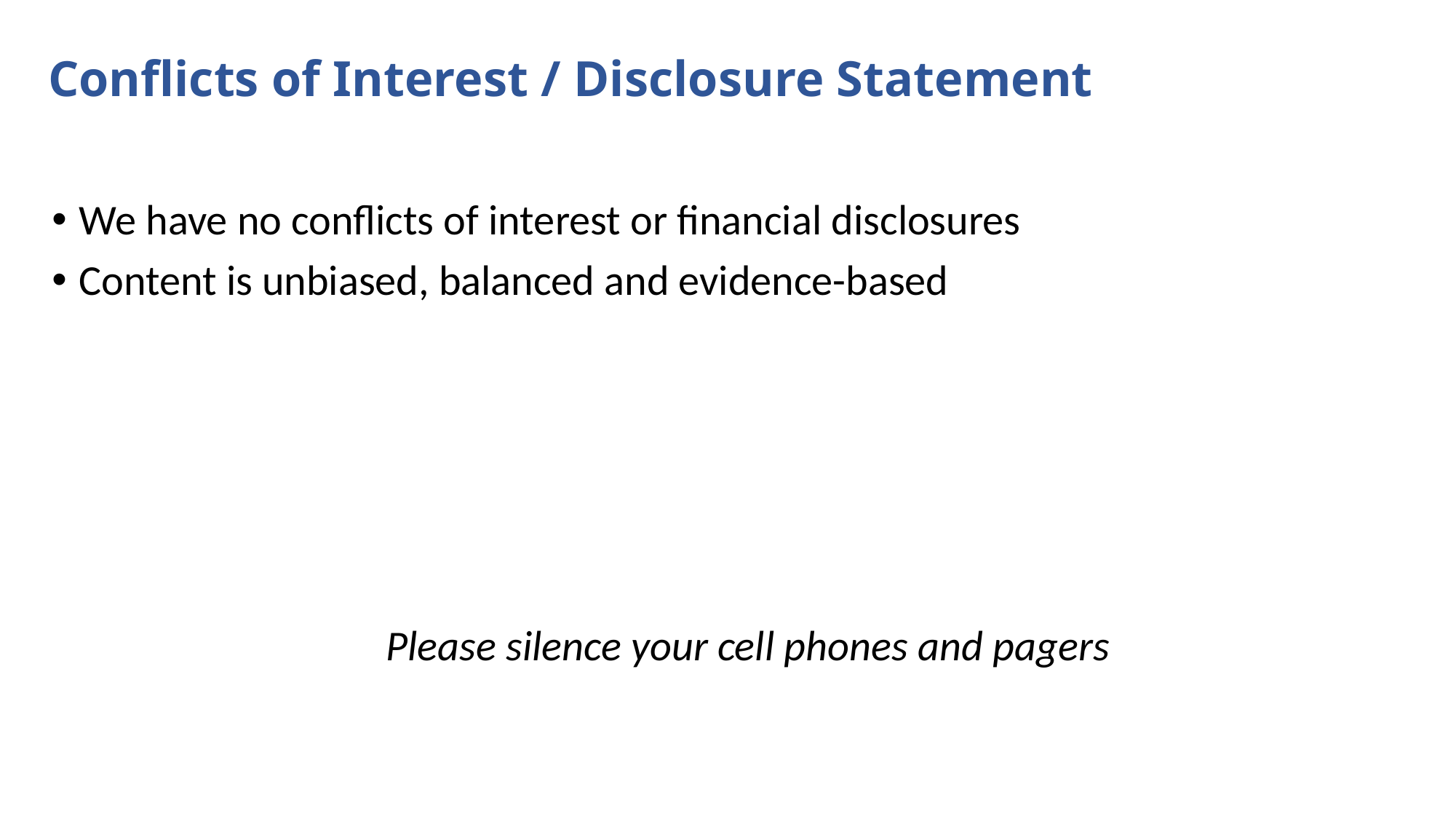

# Conflicts of Interest / Disclosure Statement
We have no conflicts of interest or financial disclosures
Content is unbiased, balanced and evidence-based
Please silence your cell phones and pagers

## Slide 3
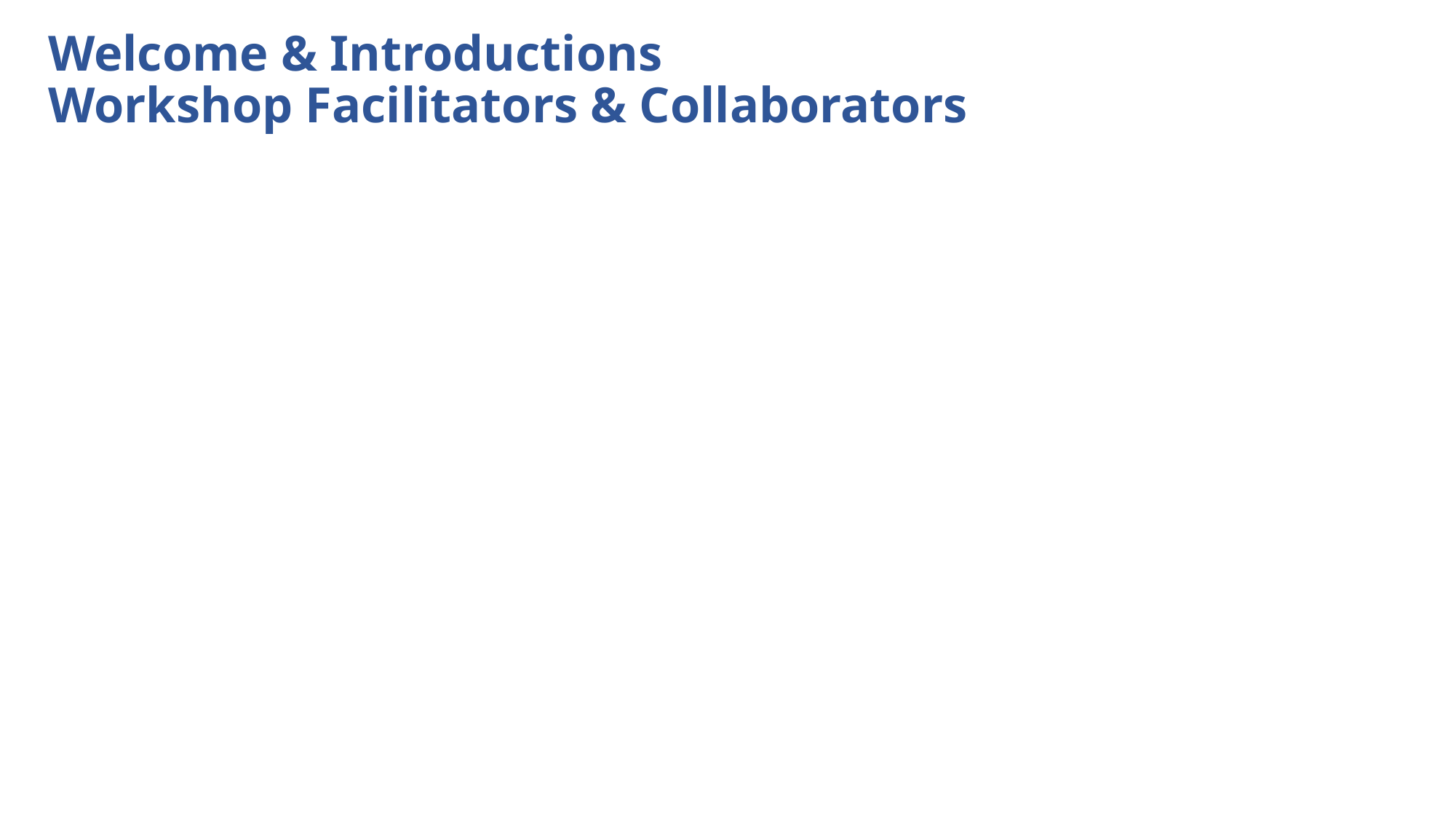

# Welcome & Introductions Workshop Facilitators & Collaborators

## Slide 4
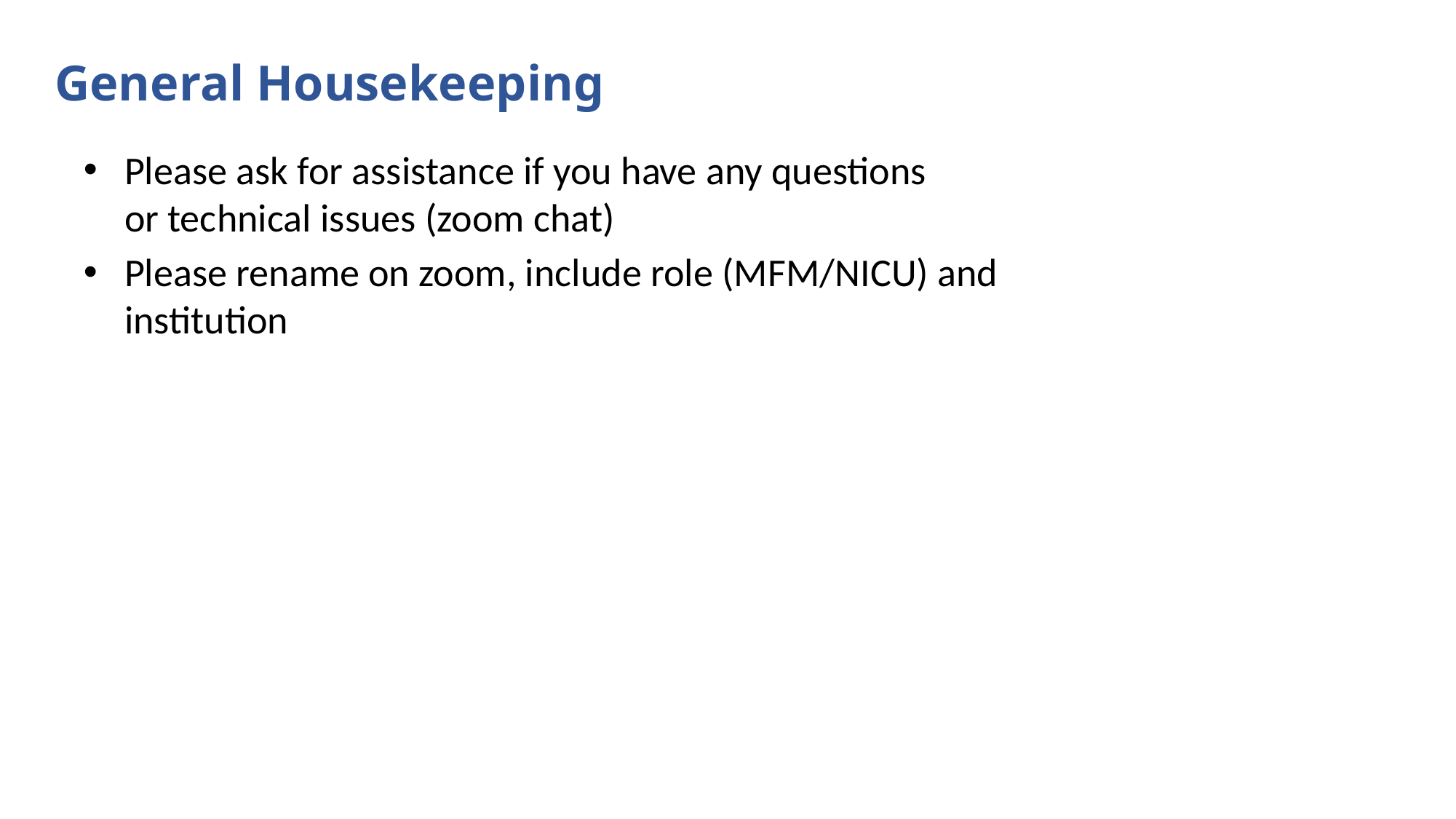

# General Housekeeping
Please ask for assistance if you have any questions
or technical issues (zoom chat)
Please rename on zoom, include role (MFM/NICU) and institution

## Slide 5
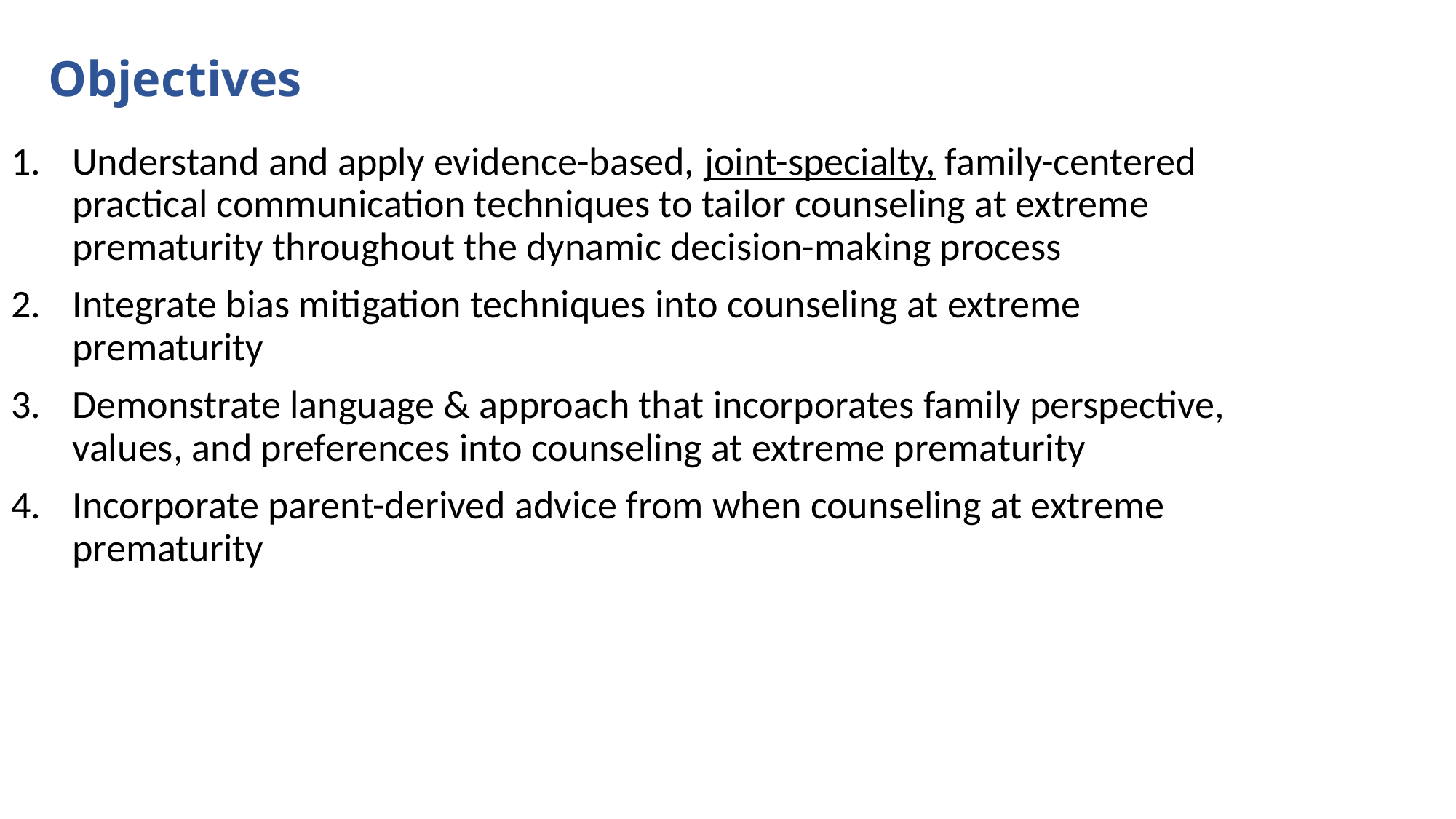

# Objectives
Understand and apply evidence-based, joint-specialty, family-centered practical communication techniques to tailor counseling at extreme prematurity throughout the dynamic decision-making process
Integrate bias mitigation techniques into counseling at extreme prematurity
Demonstrate language & approach that incorporates family perspective, values, and preferences into counseling at extreme prematurity
Incorporate parent-derived advice from when counseling at extreme prematurity

## Slide 6
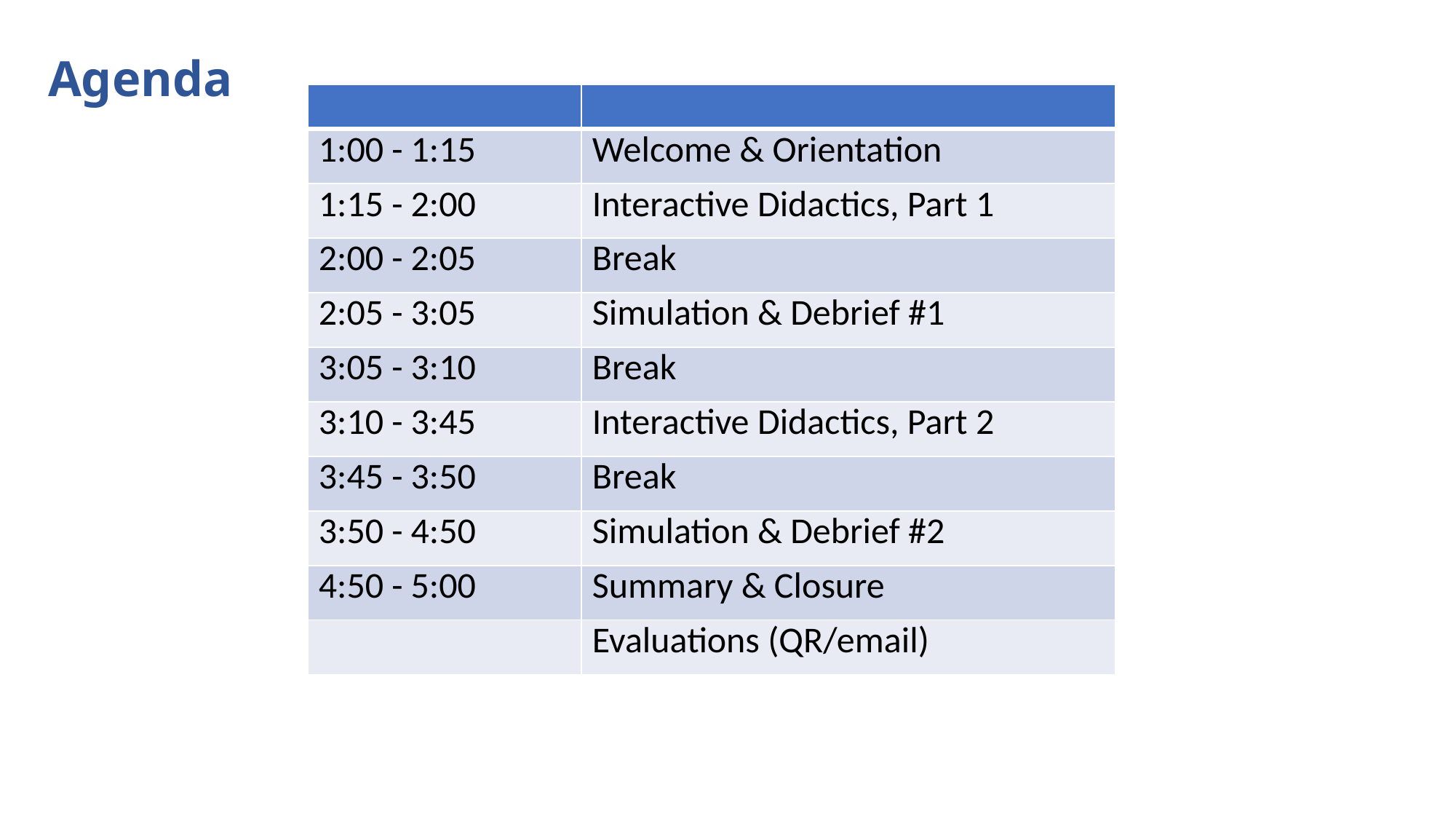

# Agenda
| | |
| --- | --- |
| 1:00 - 1:15 | Welcome & Orientation |
| 1:15 - 2:00 | Interactive Didactics, Part 1 |
| 2:00 - 2:05 | Break |
| 2:05 - 3:05 | Simulation & Debrief #1 |
| 3:05 - 3:10 | Break |
| 3:10 - 3:45 | Interactive Didactics, Part 2 |
| 3:45 - 3:50 | Break |
| 3:50 - 4:50 | Simulation & Debrief #2 |
| 4:50 - 5:00 | Summary & Closure |
| | Evaluations (QR/email) |

## Slide 7
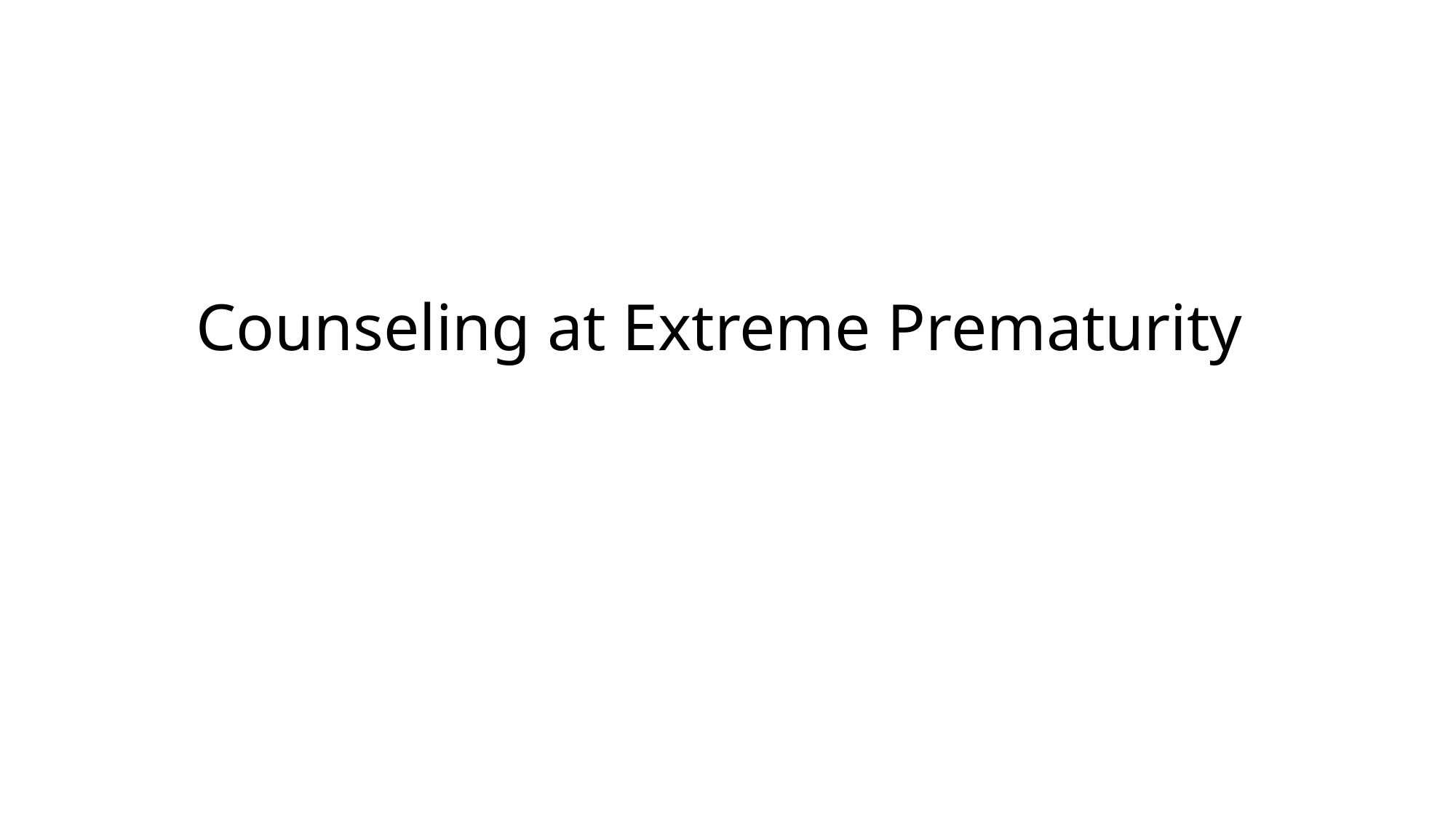

# Counseling at Extreme Prematurity

## Slide 8
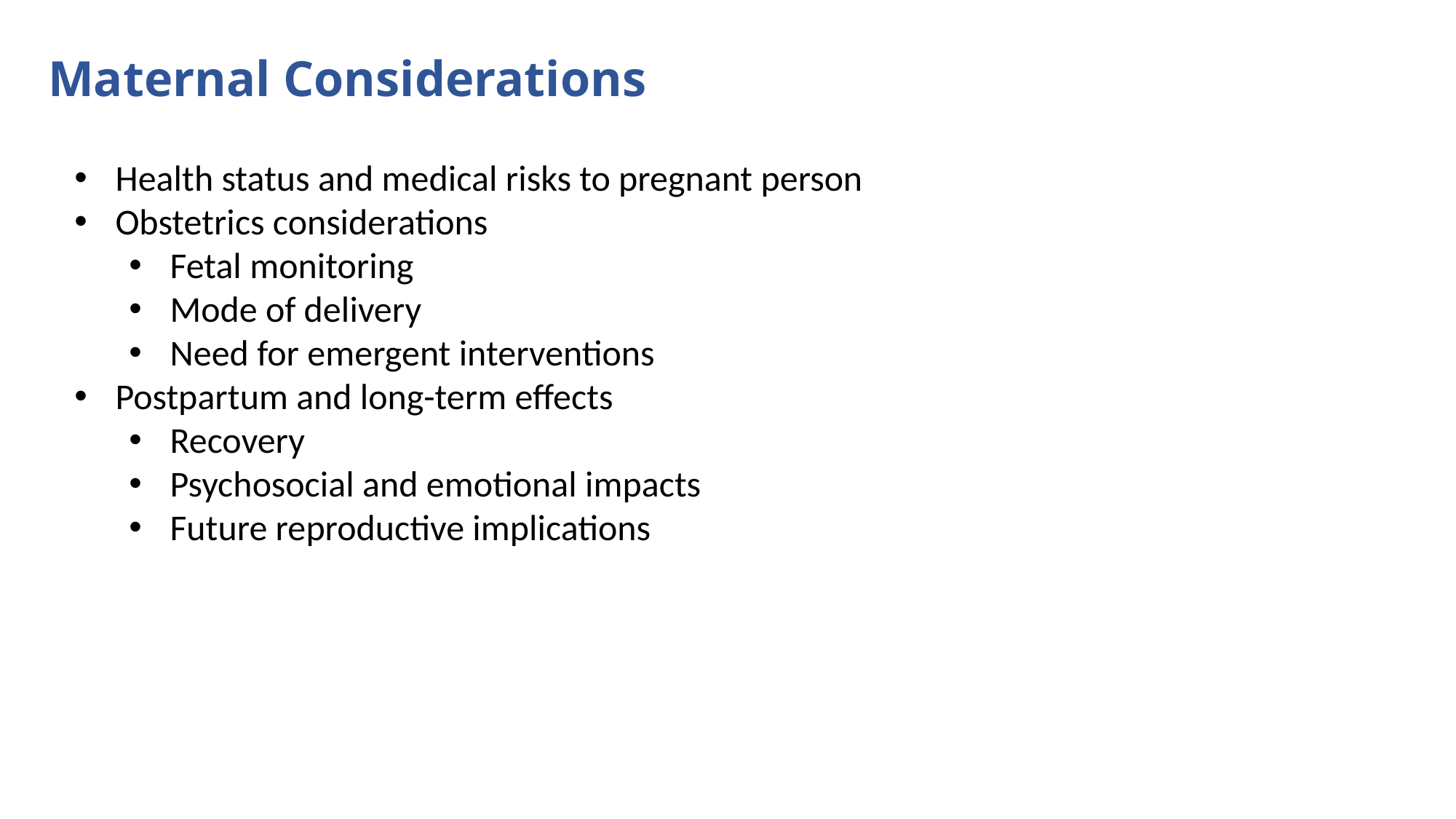

# Maternal Considerations
Health status and medical risks to pregnant person
Obstetrics considerations
Fetal monitoring
Mode of delivery
Need for emergent interventions
Postpartum and long-term effects
Recovery
Psychosocial and emotional impacts
Future reproductive implications

## Slide 9
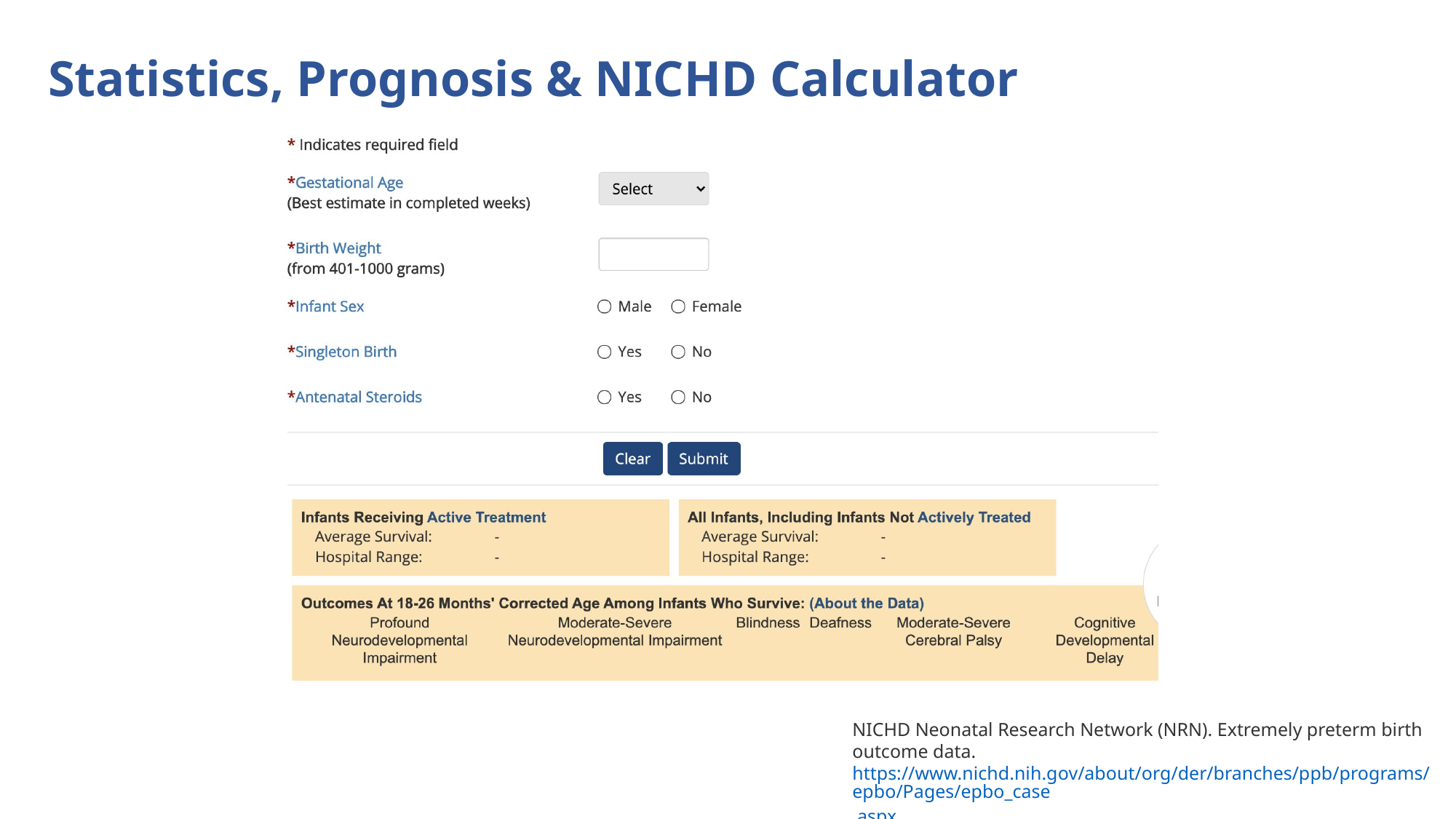

# Statistics, Prognosis & NICHD Calculator
NICHD Neonatal Research Network (NRN). Extremely preterm birth outcome data. https://www.nichd.nih.gov/about/org/der/branches/ppb/programs/epbo/Pages/epbo_case.aspx

## Slide 10
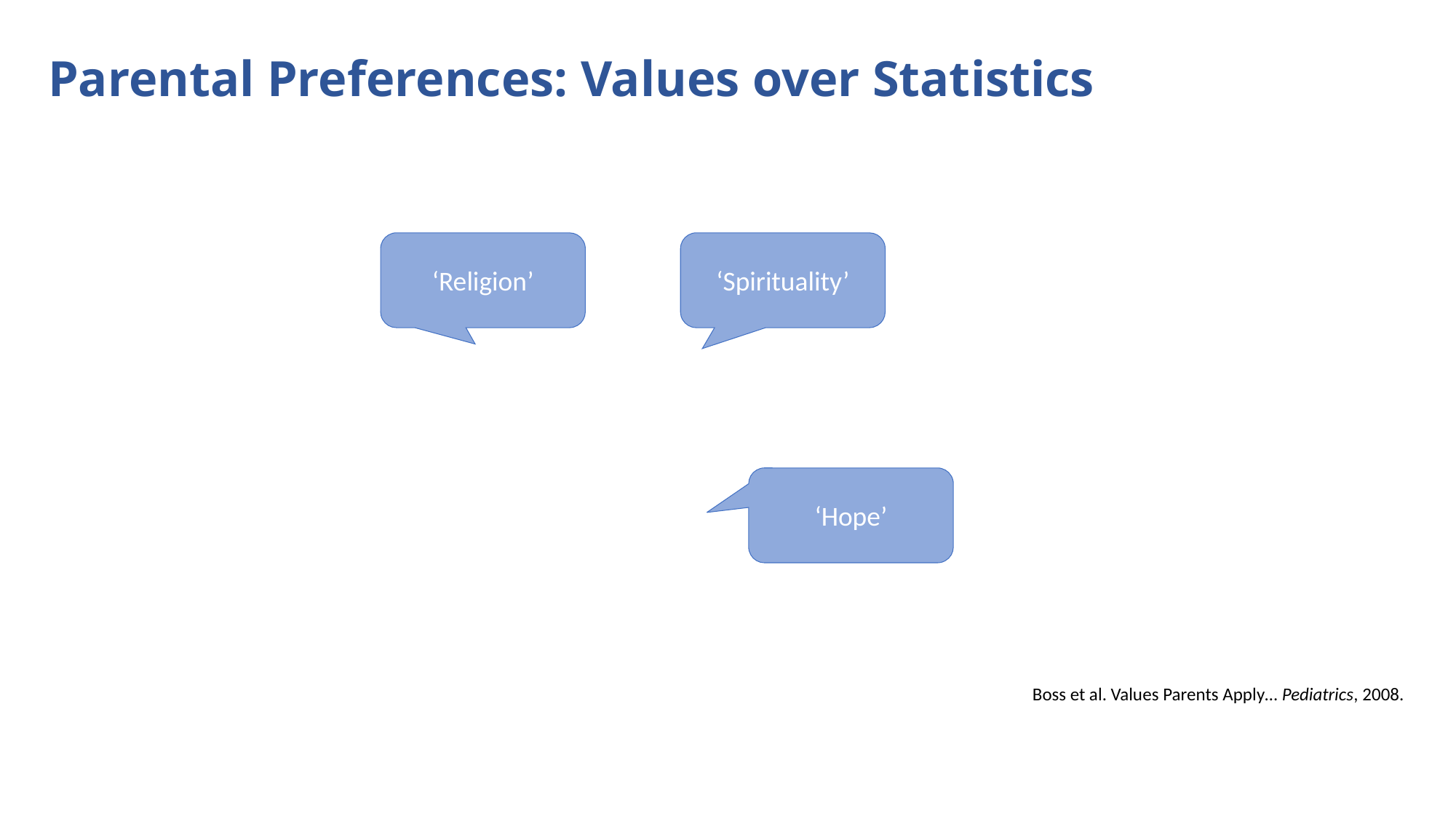

# Parental Preferences: Values over Statistics
‘Religion’
‘Spirituality’
‘Hope’
Boss et al. Values Parents Apply… Pediatrics, 2008.

## Slide 11
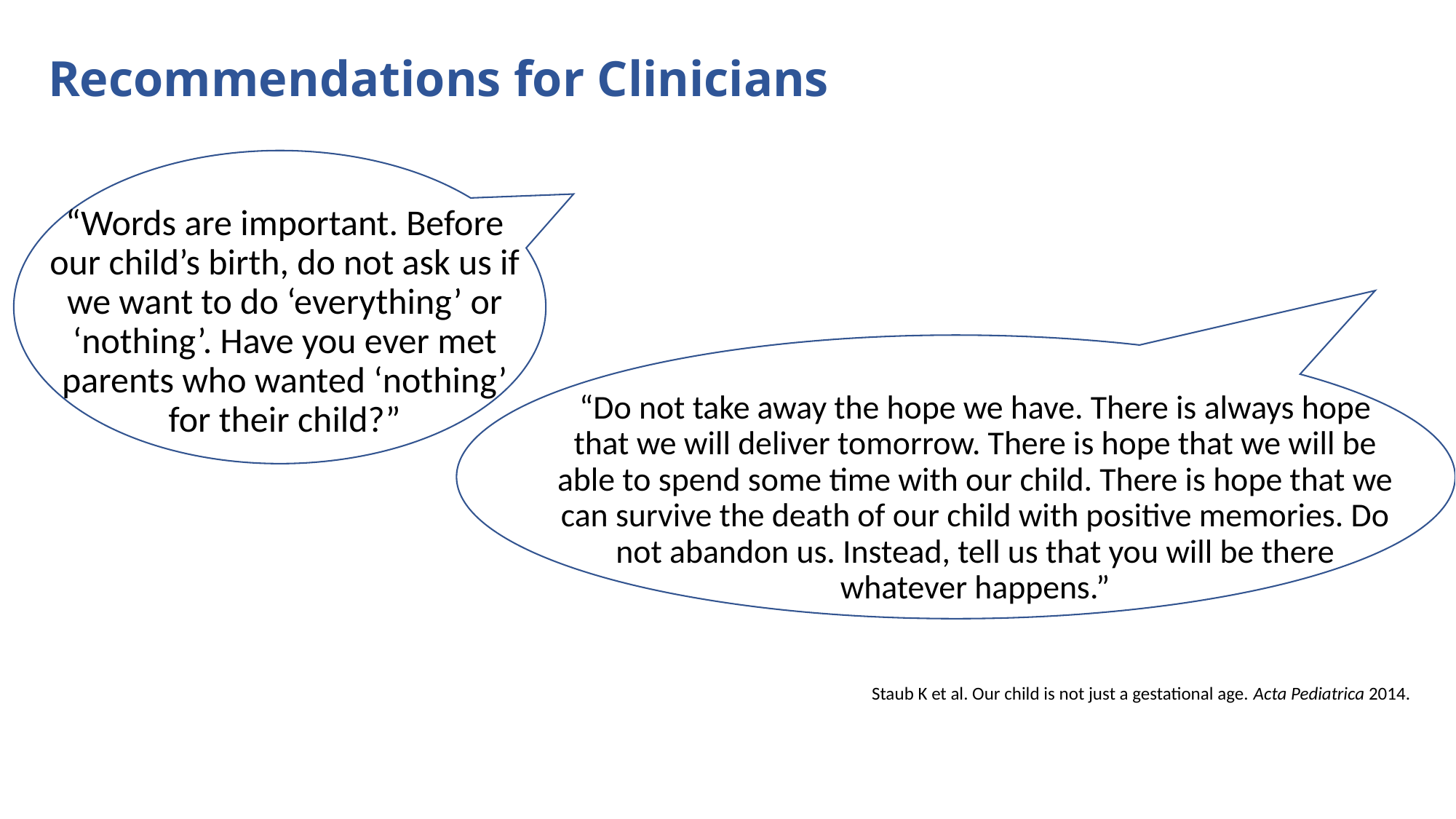

# Recommendations for Clinicians
“Words are important. Before our child’s birth, do not ask us if we want to do ‘everything’ or ‘nothing’. Have you ever met parents who wanted ‘nothing’ for their child?”
“Do not take away the hope we have. There is always hope that we will deliver tomorrow. There is hope that we will be able to spend some time with our child. There is hope that we can survive the death of our child with positive memories. Do not abandon us. Instead, tell us that you will be there whatever happens.”
Staub K et al. Our child is not just a gestational age. Acta Pediatrica 2014.

## Slide 12
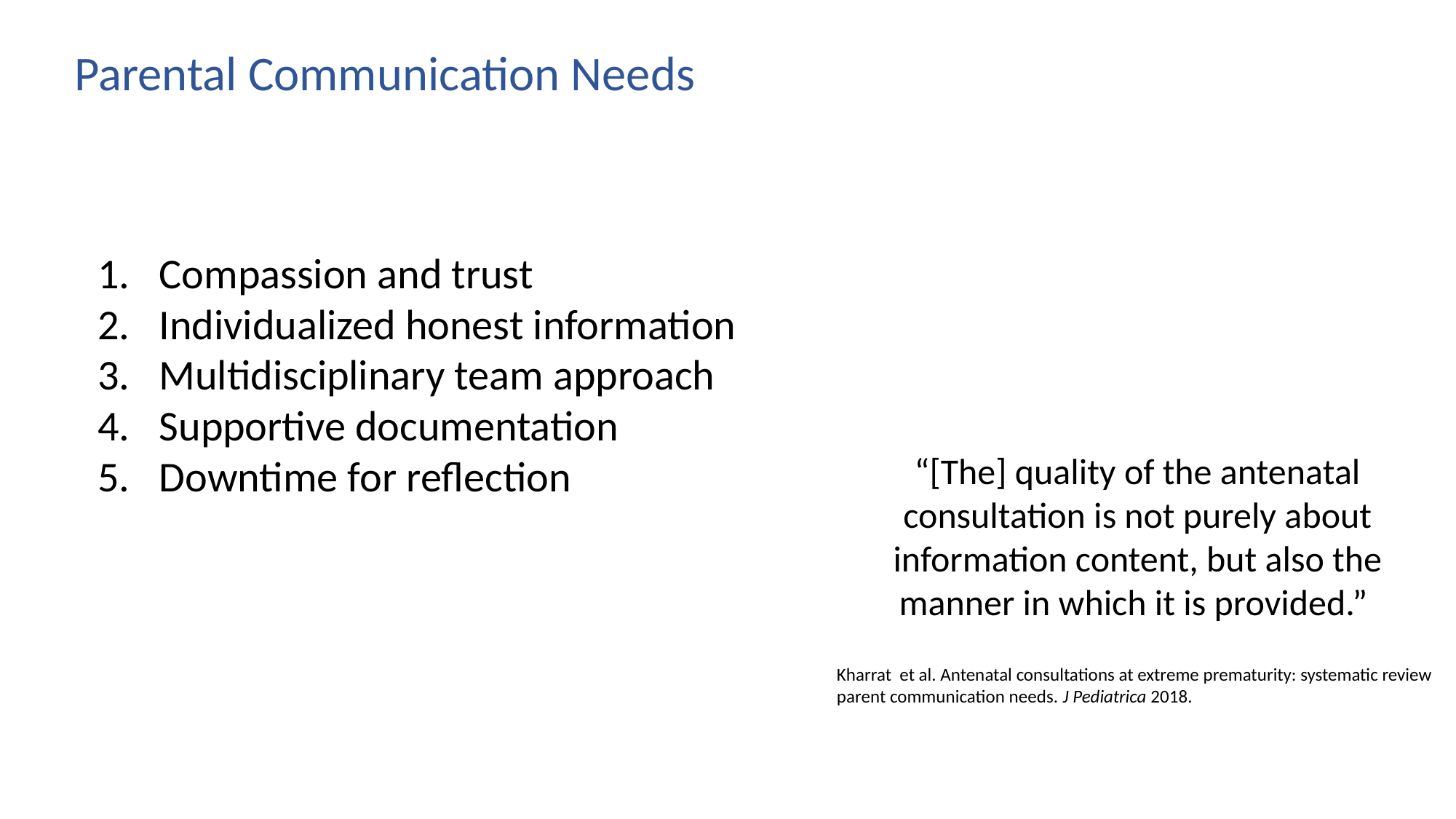

Parental Communication Needs
Compassion and trust
Individualized honest information
Multidisciplinary team approach
Supportive documentation
Downtime for reflection
“[The] quality of the antenatal consultation is not purely about information content, but also the manner in which it is provided.”
Kharrat et al. Antenatal consultations at extreme prematurity: systematic review parent communication needs. J Pediatrica 2018.

## Slide 13
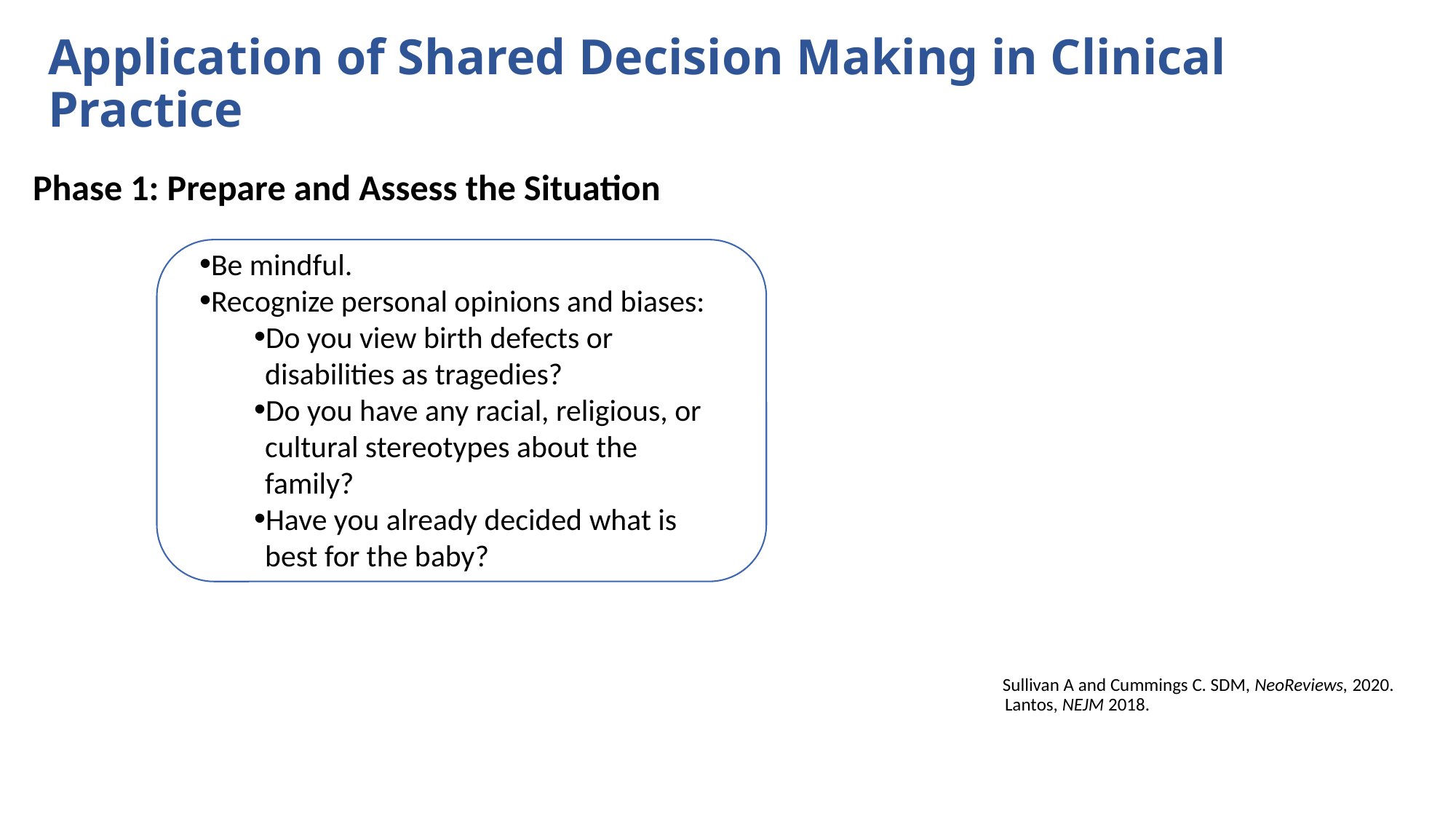

# Application of Shared Decision Making in Clinical Practice
Phase 1: Prepare and Assess the Situation
Be mindful.
Recognize personal opinions and biases:
Do you view birth defects or disabilities as tragedies?
Do you have any racial, religious, or cultural stereotypes about the family?
Have you already decided what is best for the baby?
Sullivan A and Cummings C. SDM, NeoReviews, 2020.
Lantos, NEJM 2018.

## Slide 14
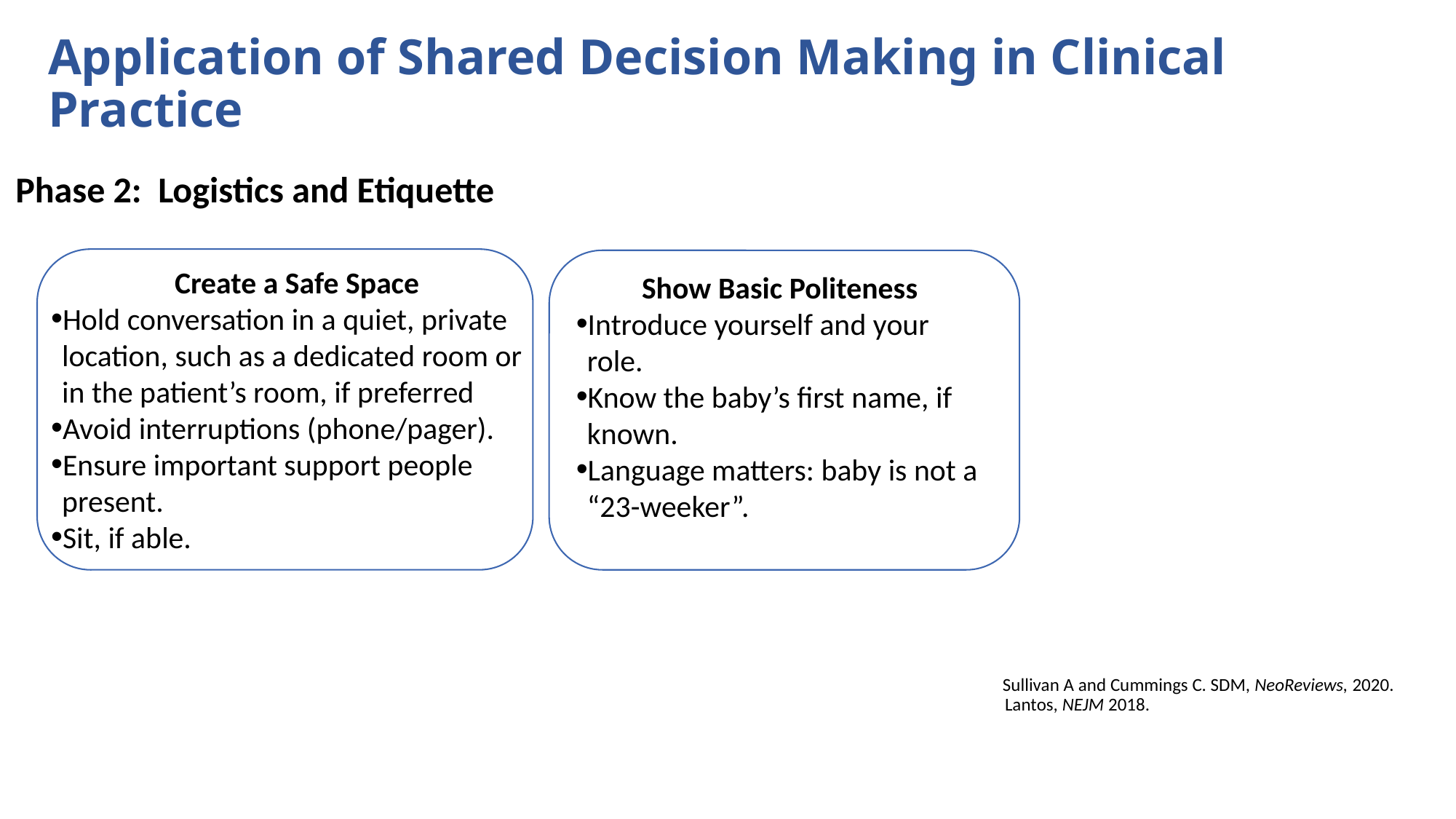

# Application of Shared Decision Making in Clinical Practice
Phase 2: Logistics and Etiquette
Create a Safe Space
Hold conversation in a quiet, private location, such as a dedicated room or in the patient’s room, if preferred
Avoid interruptions (phone/pager).
Ensure important support people present.
Sit, if able.
Show Basic Politeness
Introduce yourself and your role.
Know the baby’s first name, if known.
Language matters: baby is not a “23-weeker”.
Sullivan A and Cummings C. SDM, NeoReviews, 2020.
Lantos, NEJM 2018.

## Slide 15
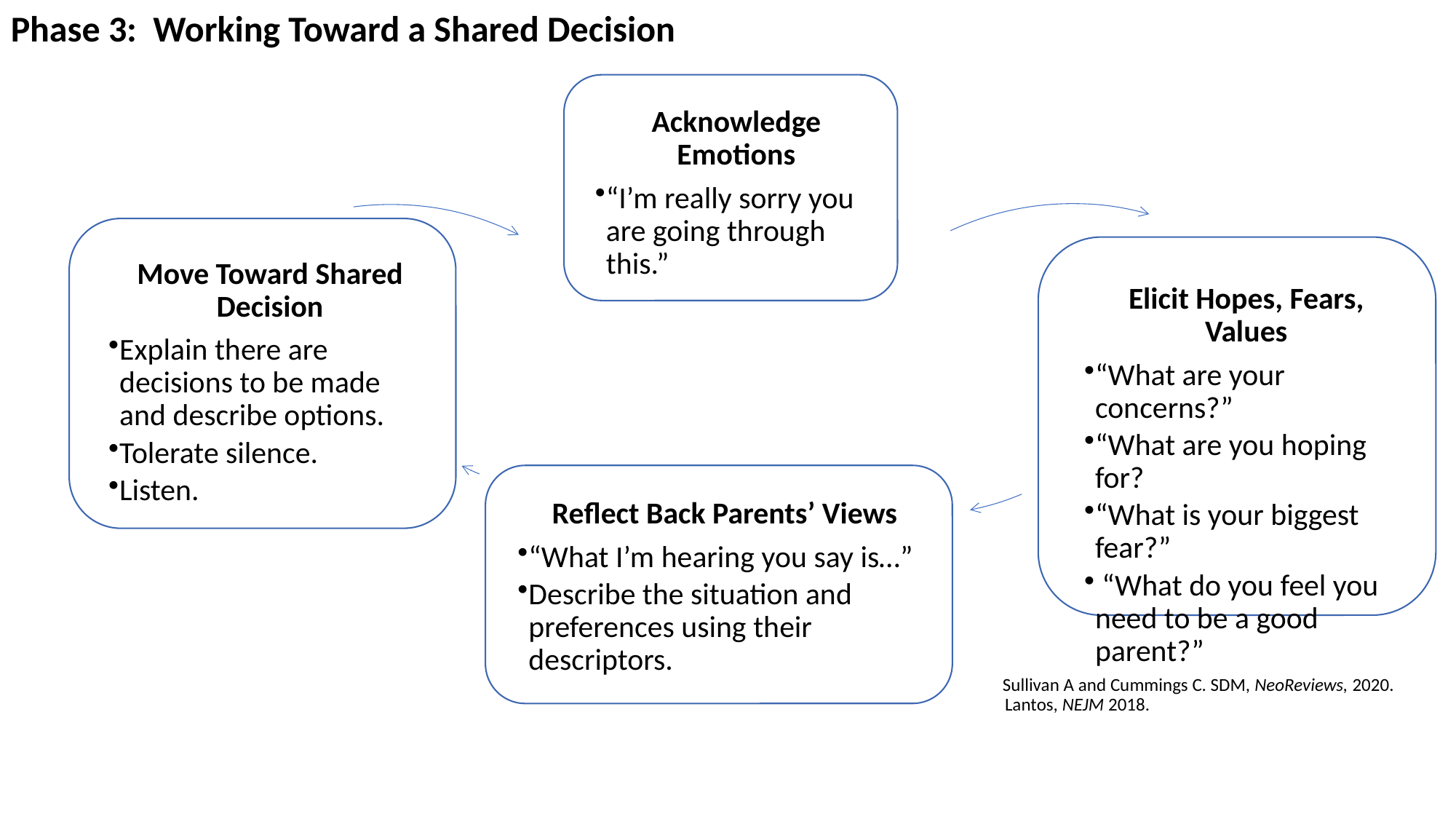

Phase 3: Working Toward a Shared Decision
Sullivan A and Cummings C. SDM, NeoReviews, 2020.
Lantos, NEJM 2018.

## Slide 16
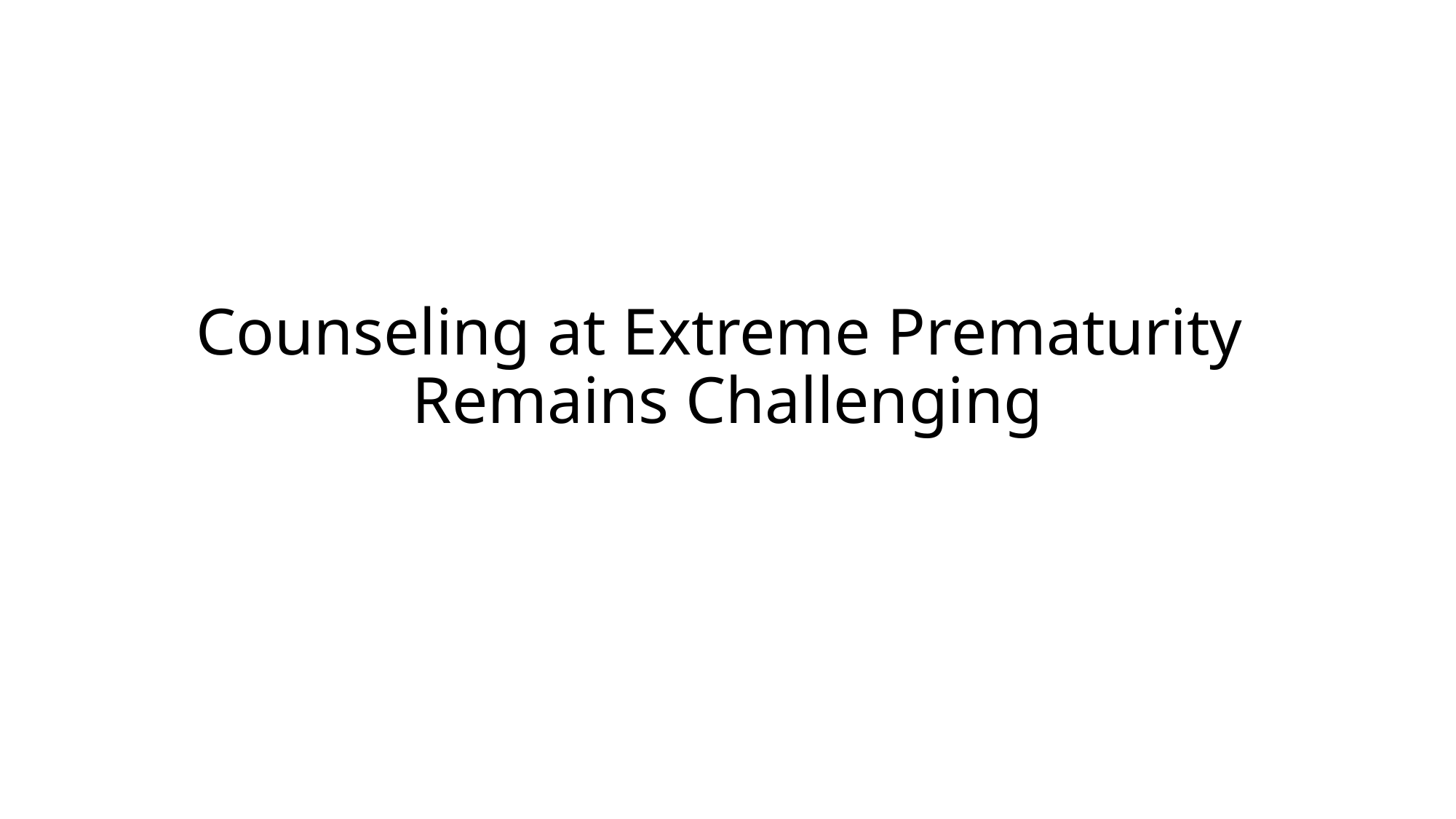

# Counseling at Extreme Prematurity Remains Challenging

## Slide 17
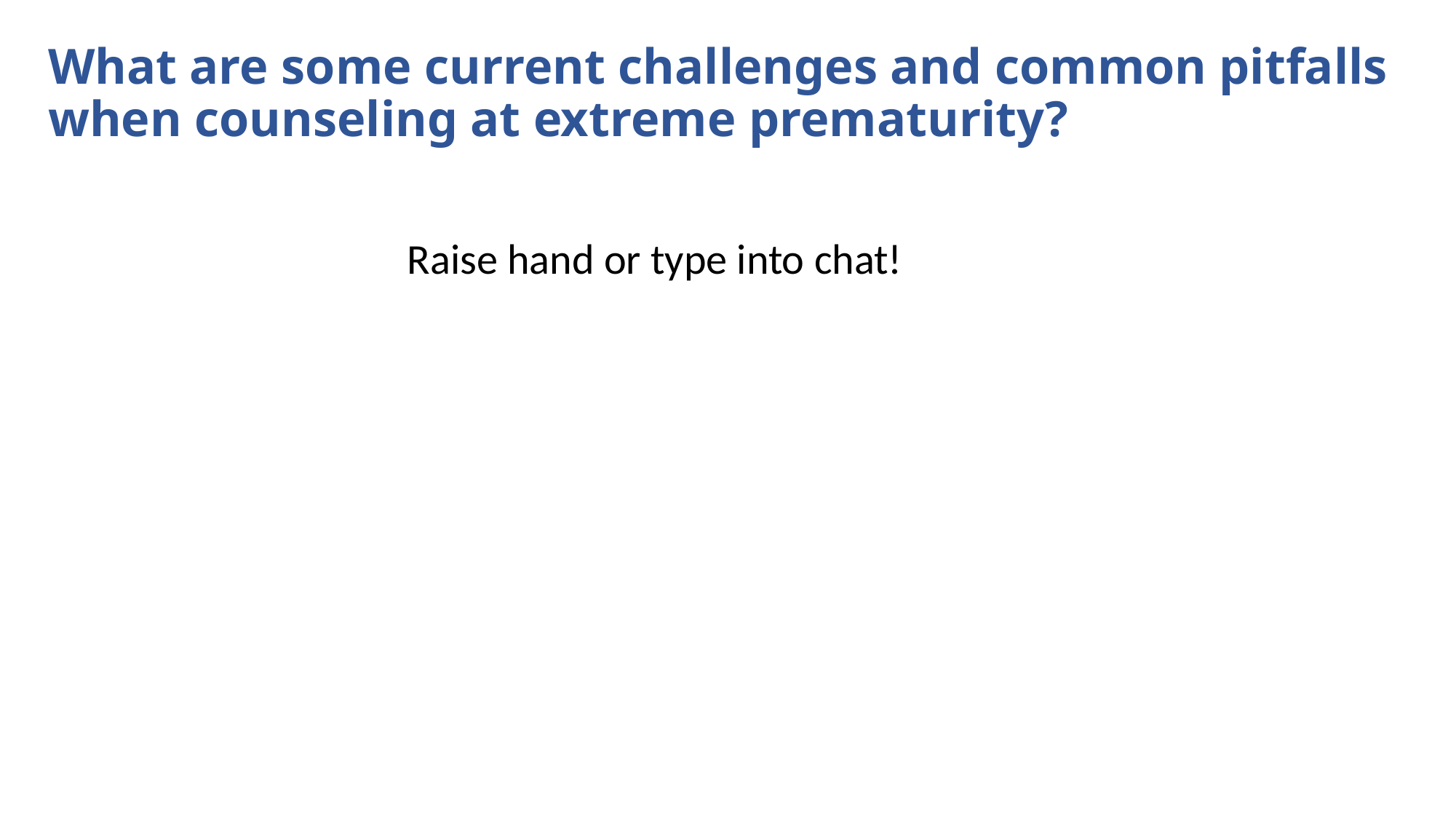

# What are some current challenges and common pitfalls when counseling at extreme prematurity?
Raise hand or type into chat!

## Slide 18
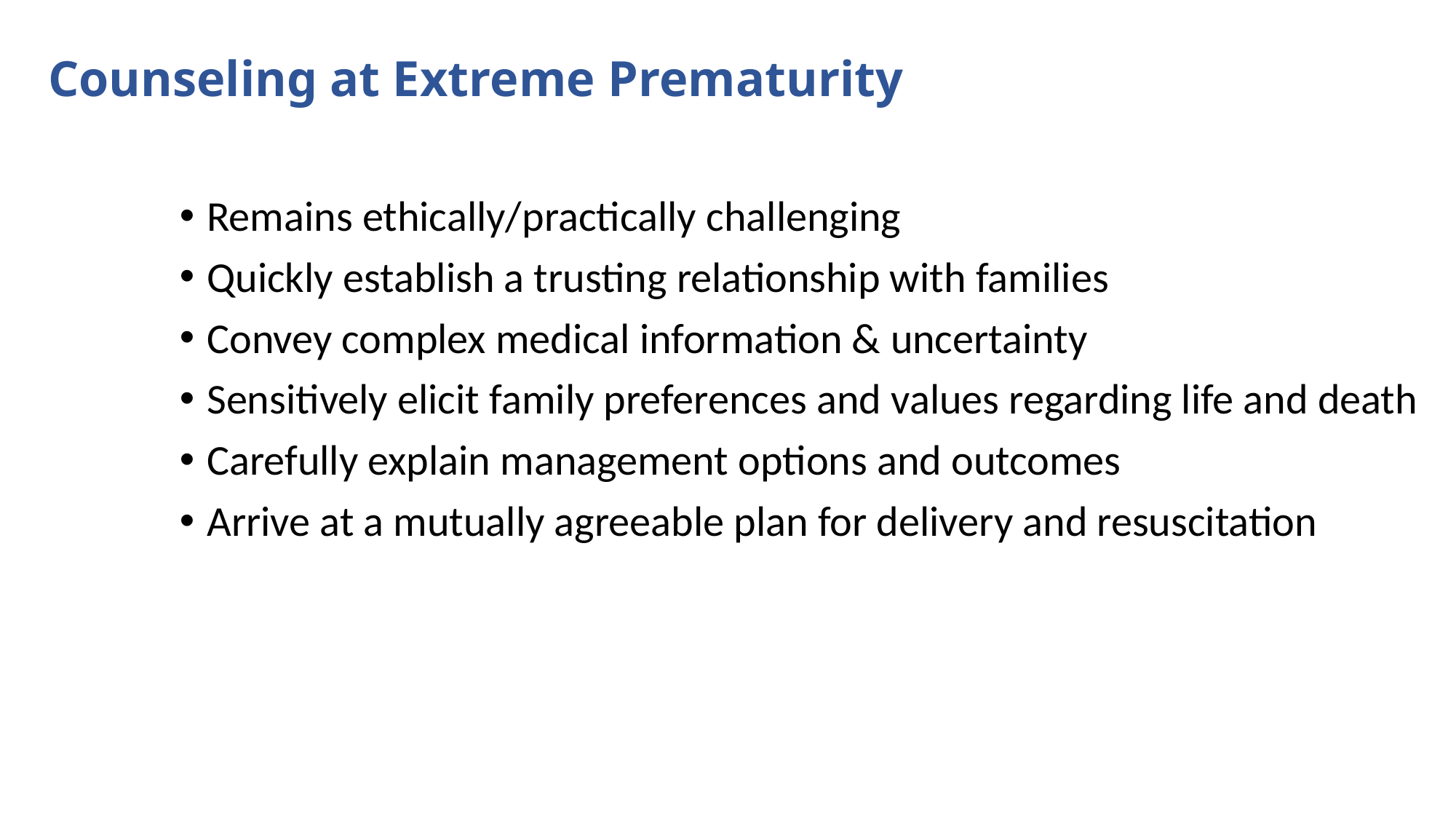

# Counseling at Extreme Prematurity
Remains ethically/practically challenging
Quickly establish a trusting relationship with families
Convey complex medical information & uncertainty
Sensitively elicit family preferences and values regarding life and death
Carefully explain management options and outcomes
Arrive at a mutually agreeable plan for delivery and resuscitation

## Slide 19
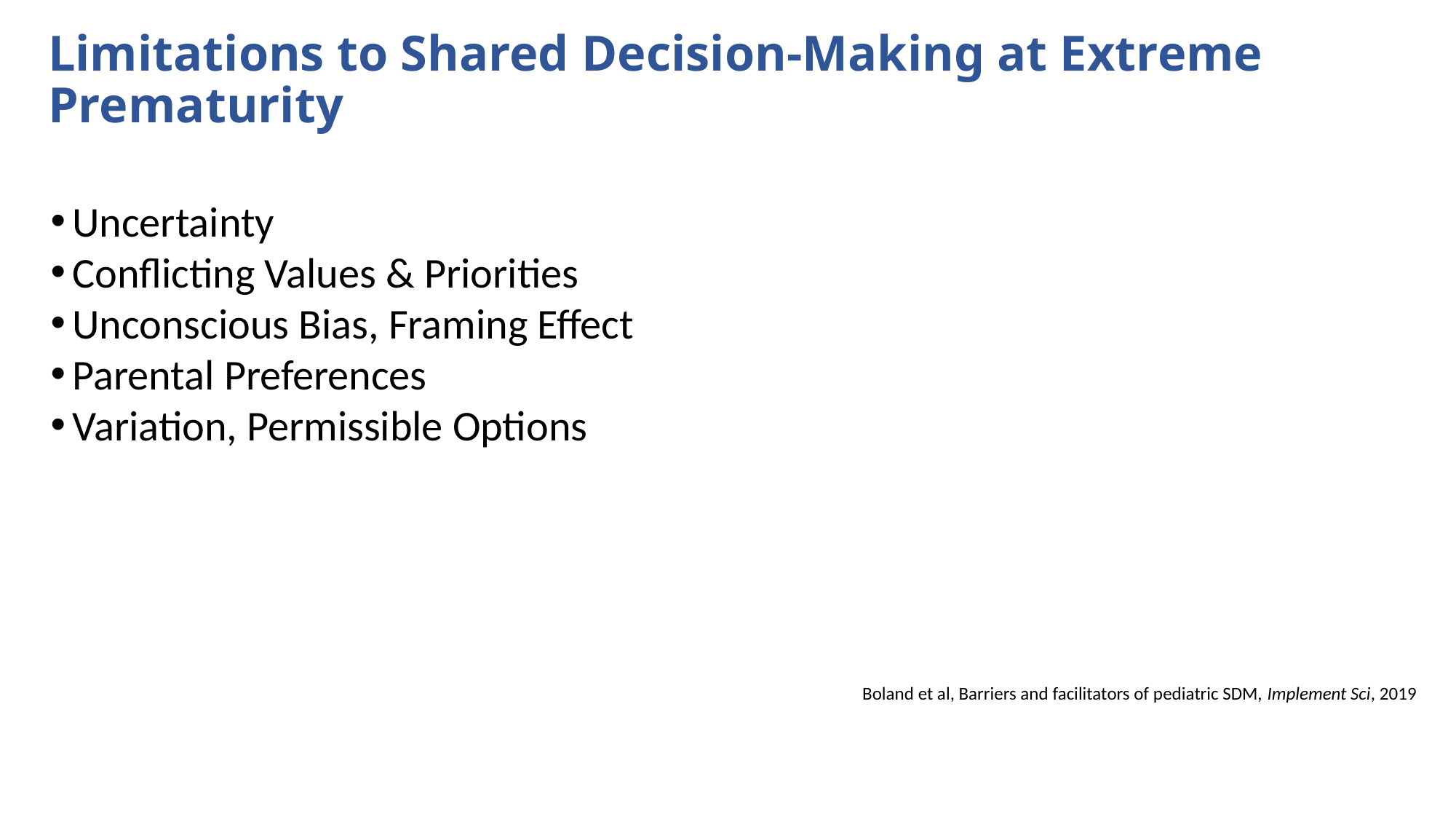

# Limitations to Shared Decision-Making at Extreme Prematurity
Uncertainty
Conflicting Values & Priorities
Unconscious Bias, Framing Effect
Parental Preferences
Variation, Permissible Options
Boland et al, Barriers and facilitators of pediatric SDM, Implement Sci, 2019

## Slide 20
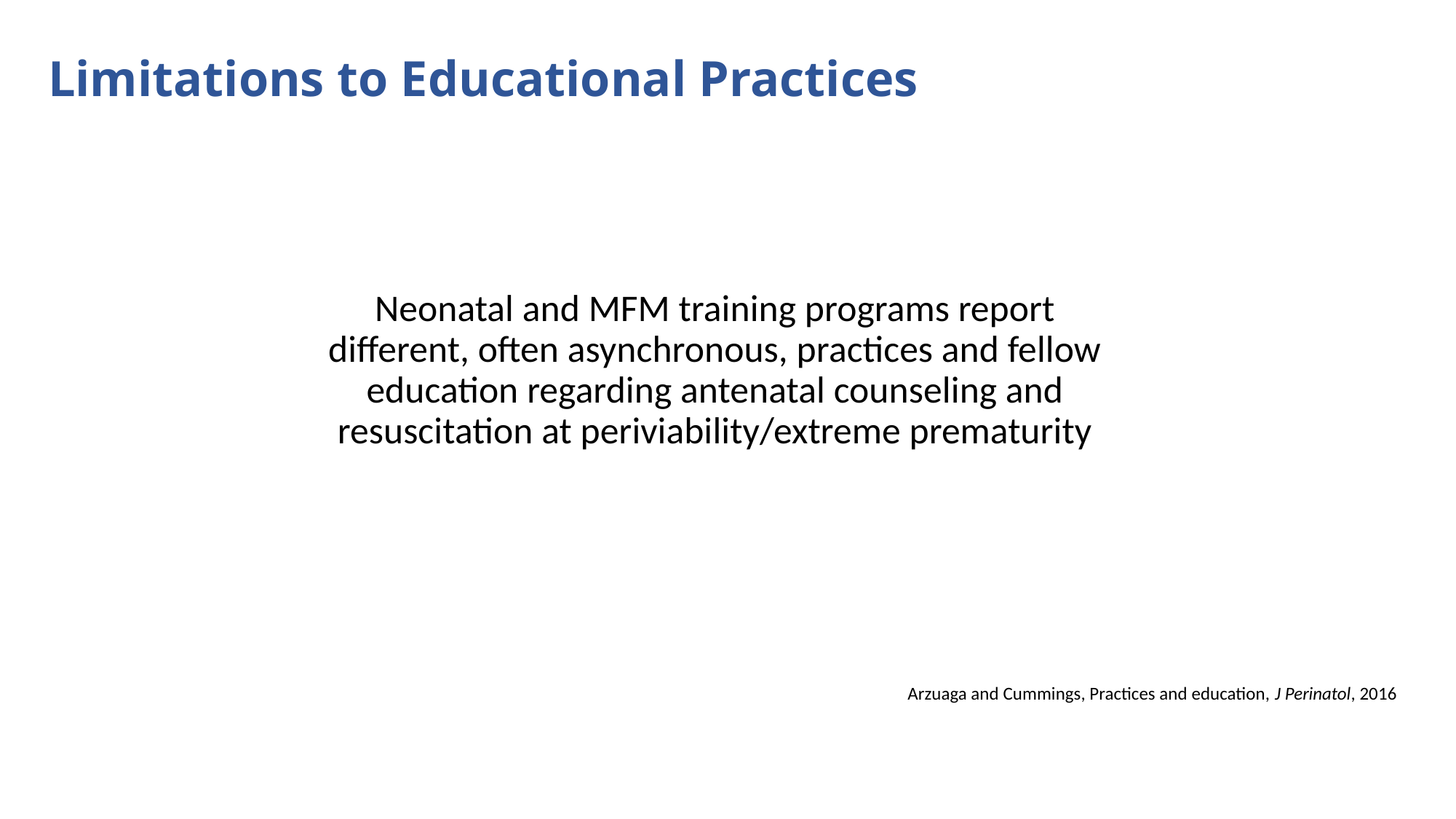

# Limitations to Educational Practices
Neonatal and MFM training programs report different, often asynchronous, practices and fellow education regarding antenatal counseling and resuscitation at periviability/extreme prematurity
Arzuaga and Cummings, Practices and education, J Perinatol, 2016

## Slide 21
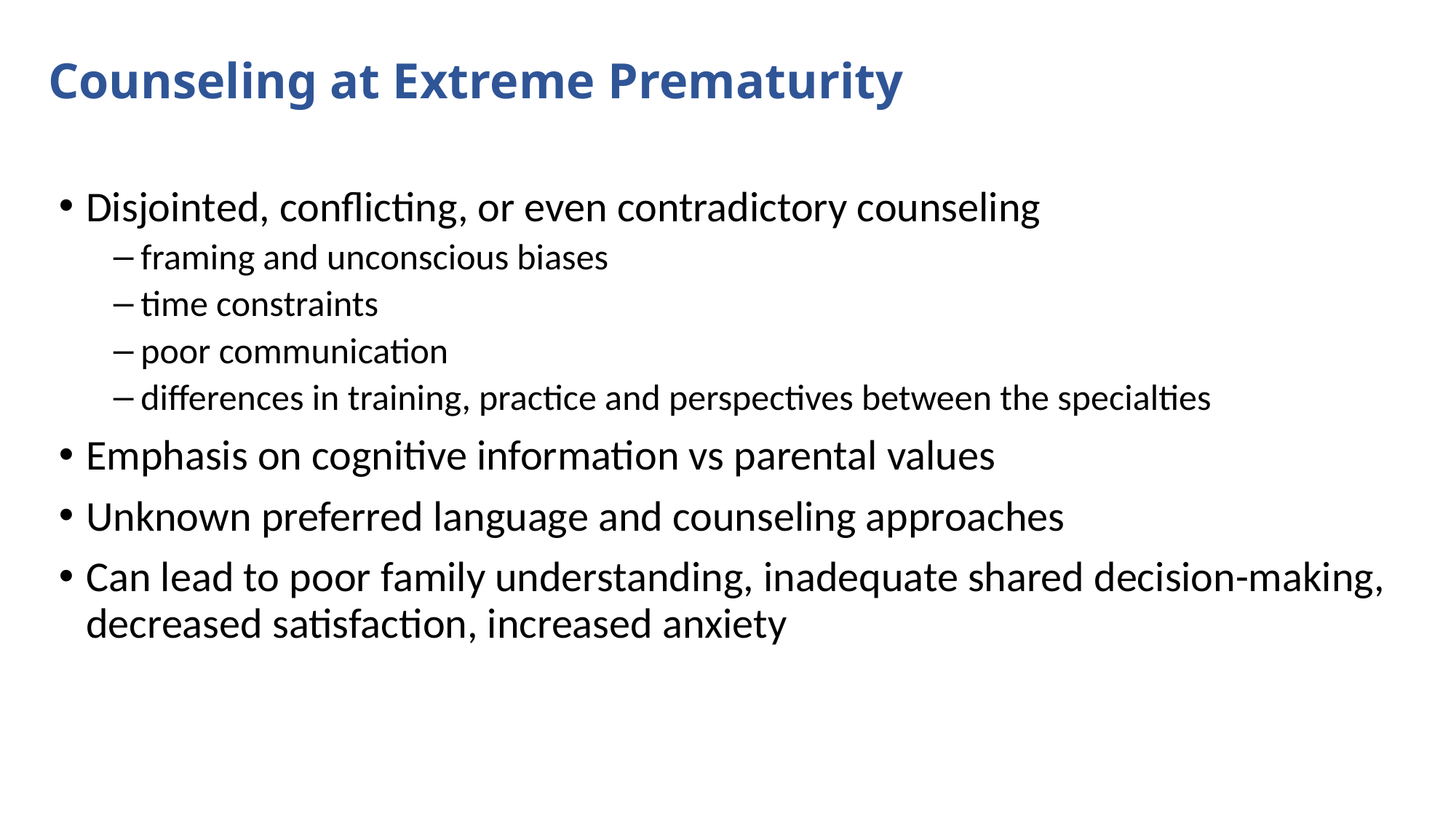

# Counseling at Extreme Prematurity
Disjointed, conflicting, or even contradictory counseling
framing and unconscious biases
time constraints
poor communication
differences in training, practice and perspectives between the specialties
Emphasis on cognitive information vs parental values
Unknown preferred language and counseling approaches
Can lead to poor family understanding, inadequate shared decision-making, decreased satisfaction, increased anxiety

## Slide 22
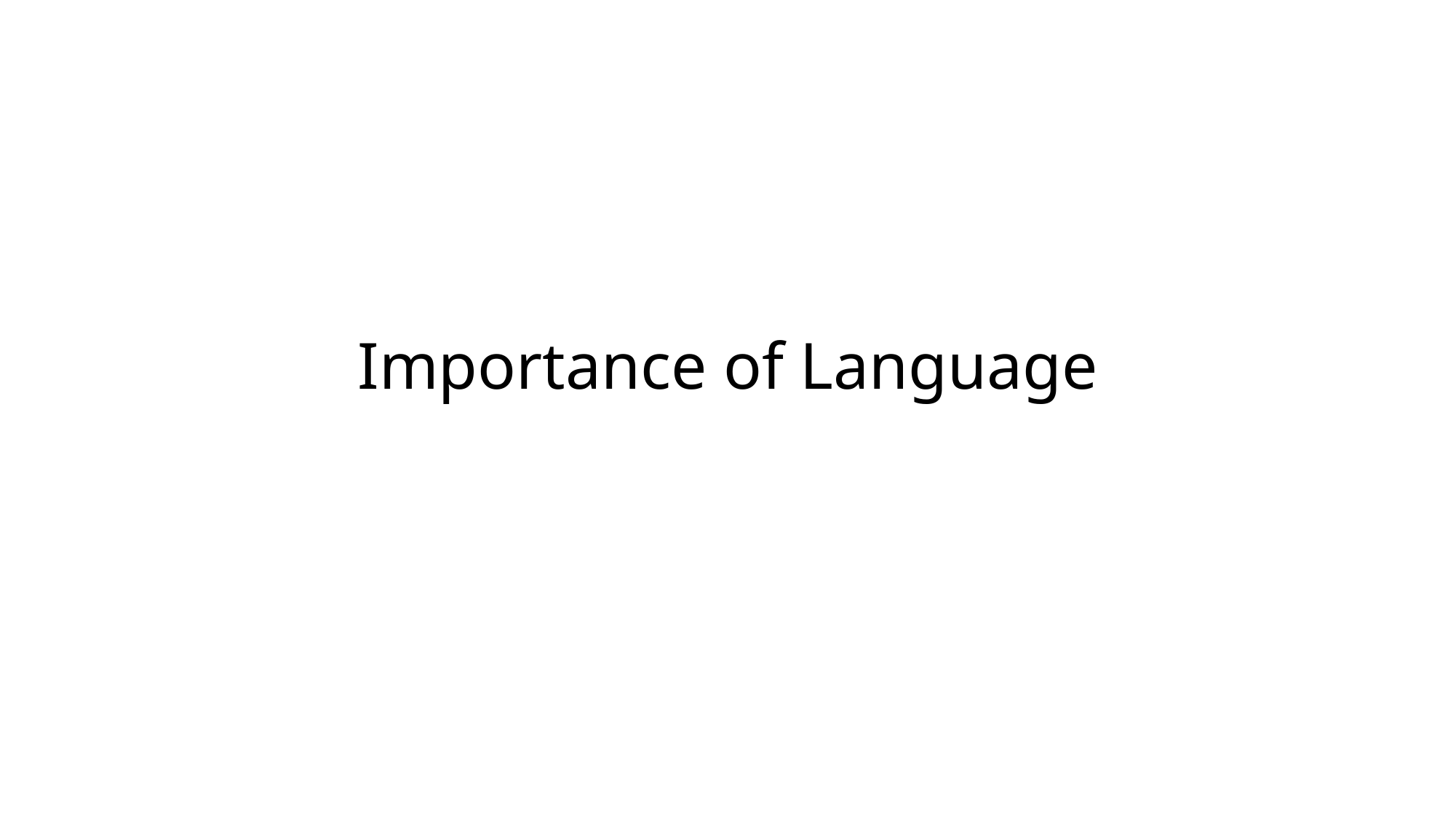

# Importance of Language

## Slide 23
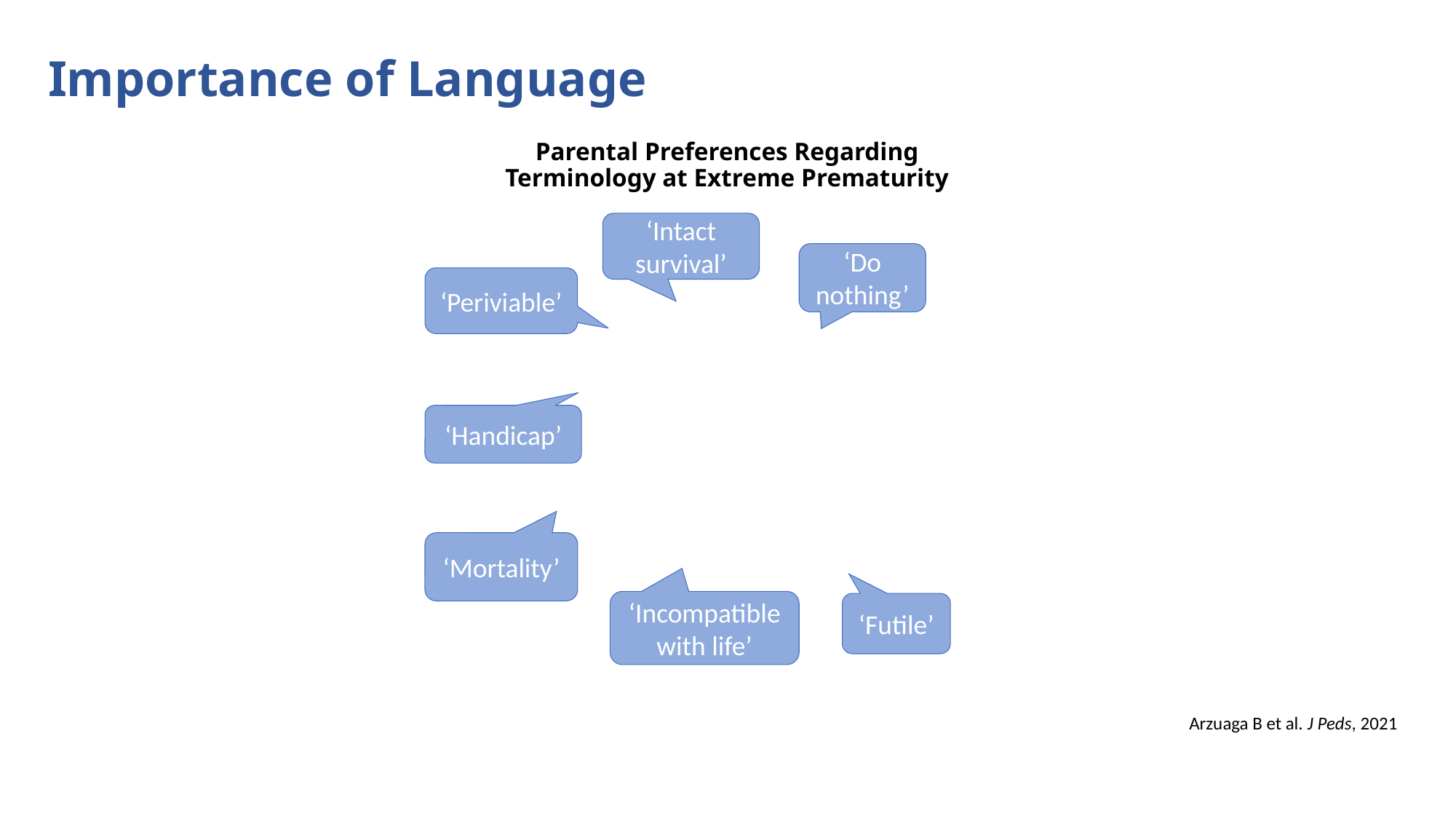

# Importance of Language
Parental Preferences Regarding Terminology at Extreme Prematurity
‘Intact survival’
‘Do nothing’
‘Periviable’
‘Handicap’
‘Mortality’
‘Incompatible with life’
‘Futile’
Arzuaga B et al. J Peds, 2021

## Slide 24
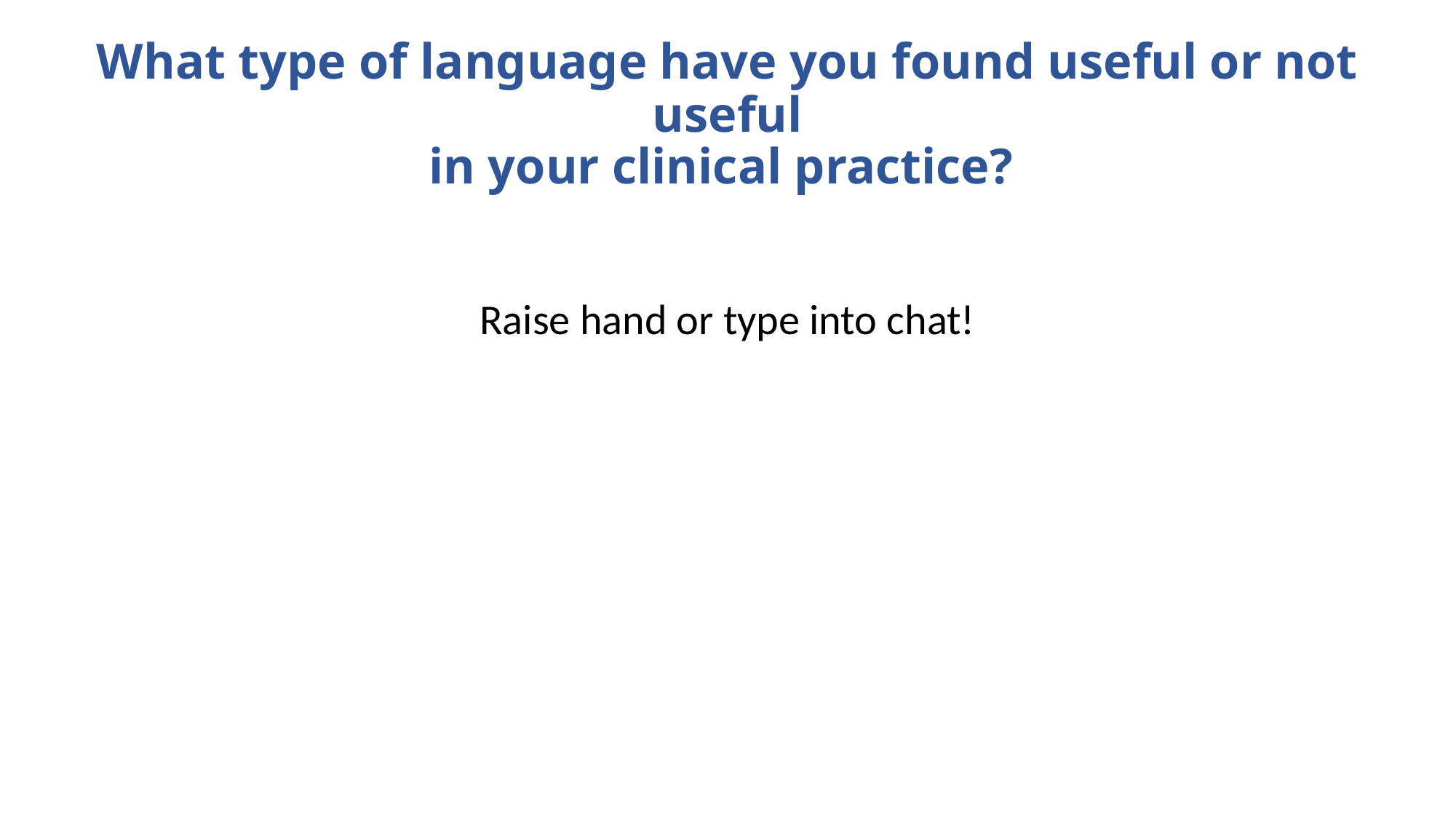

# What type of language have you found useful or not useful in your clinical practice?
Raise hand or type into chat!

## Slide 25
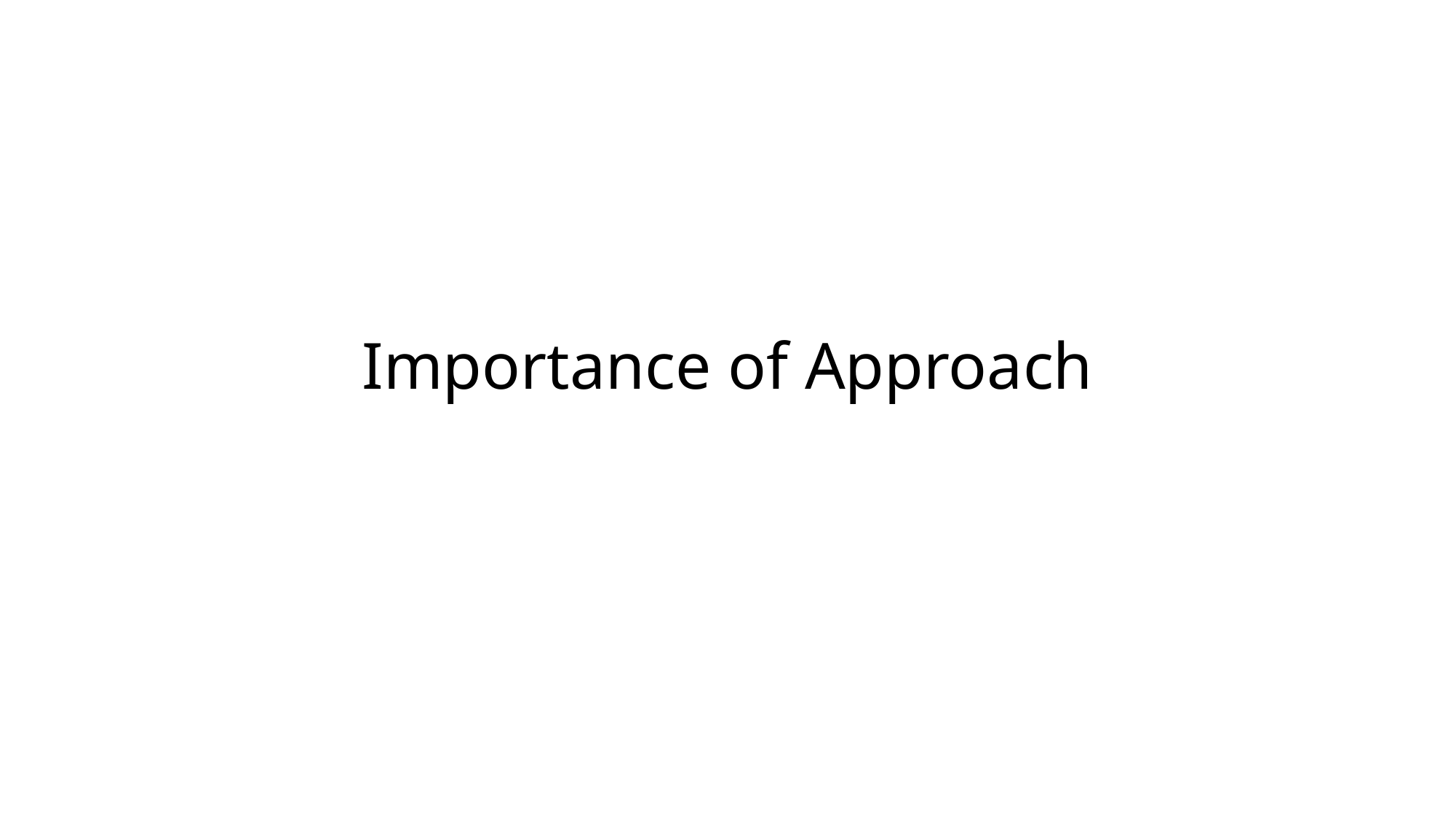

# Importance of Approach

## Slide 26
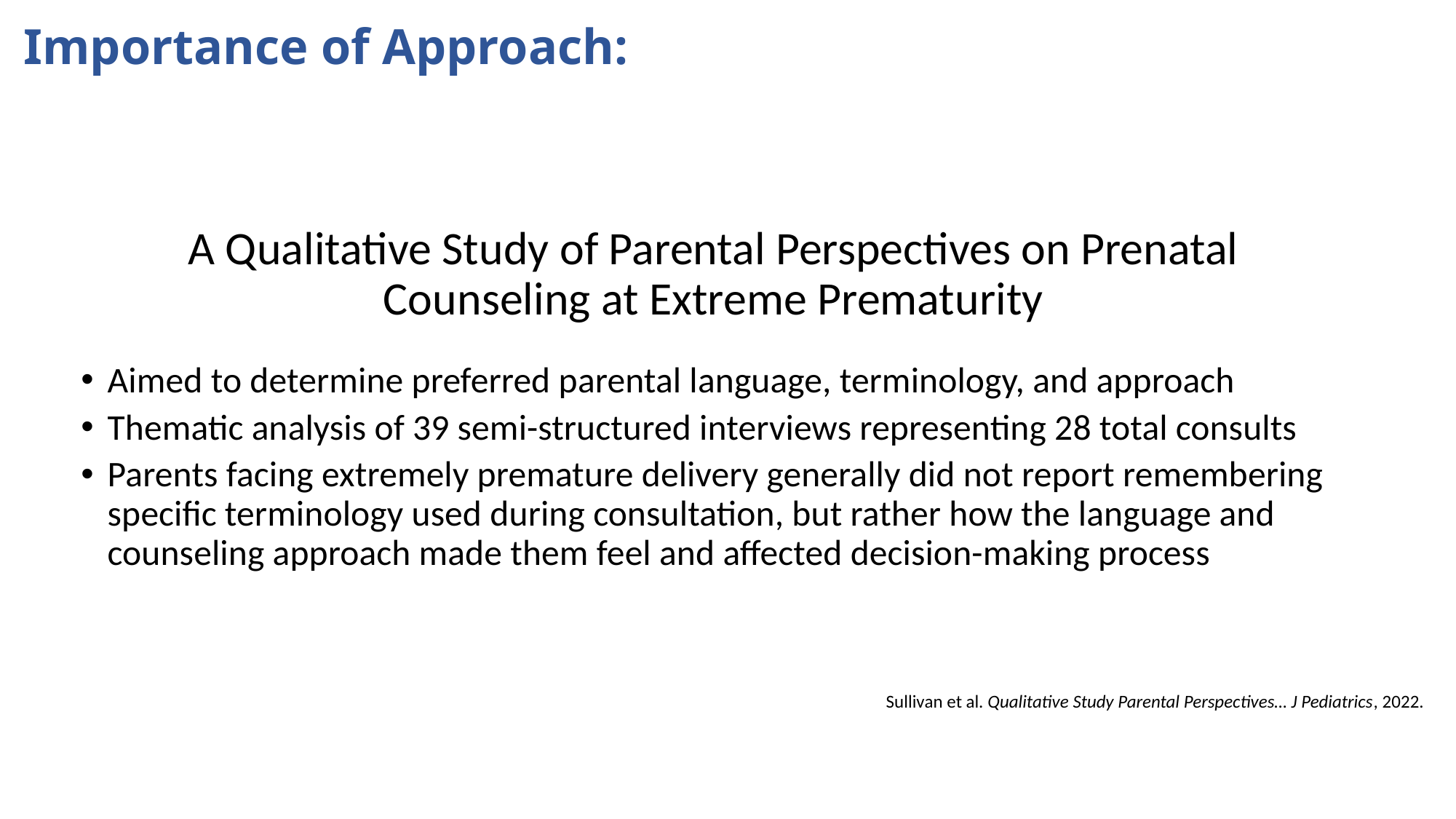

# Importance of Approach:
A Qualitative Study of Parental Perspectives on Prenatal Counseling at Extreme Prematurity
Aimed to determine preferred parental language, terminology, and approach
Thematic analysis of 39 semi-structured interviews representing 28 total consults
Parents facing extremely premature delivery generally did not report remembering specific terminology used during consultation, but rather how the language and counseling approach made them feel and affected decision-making process
Sullivan et al. Qualitative Study Parental Perspectives… J Pediatrics, 2022.

## Slide 27
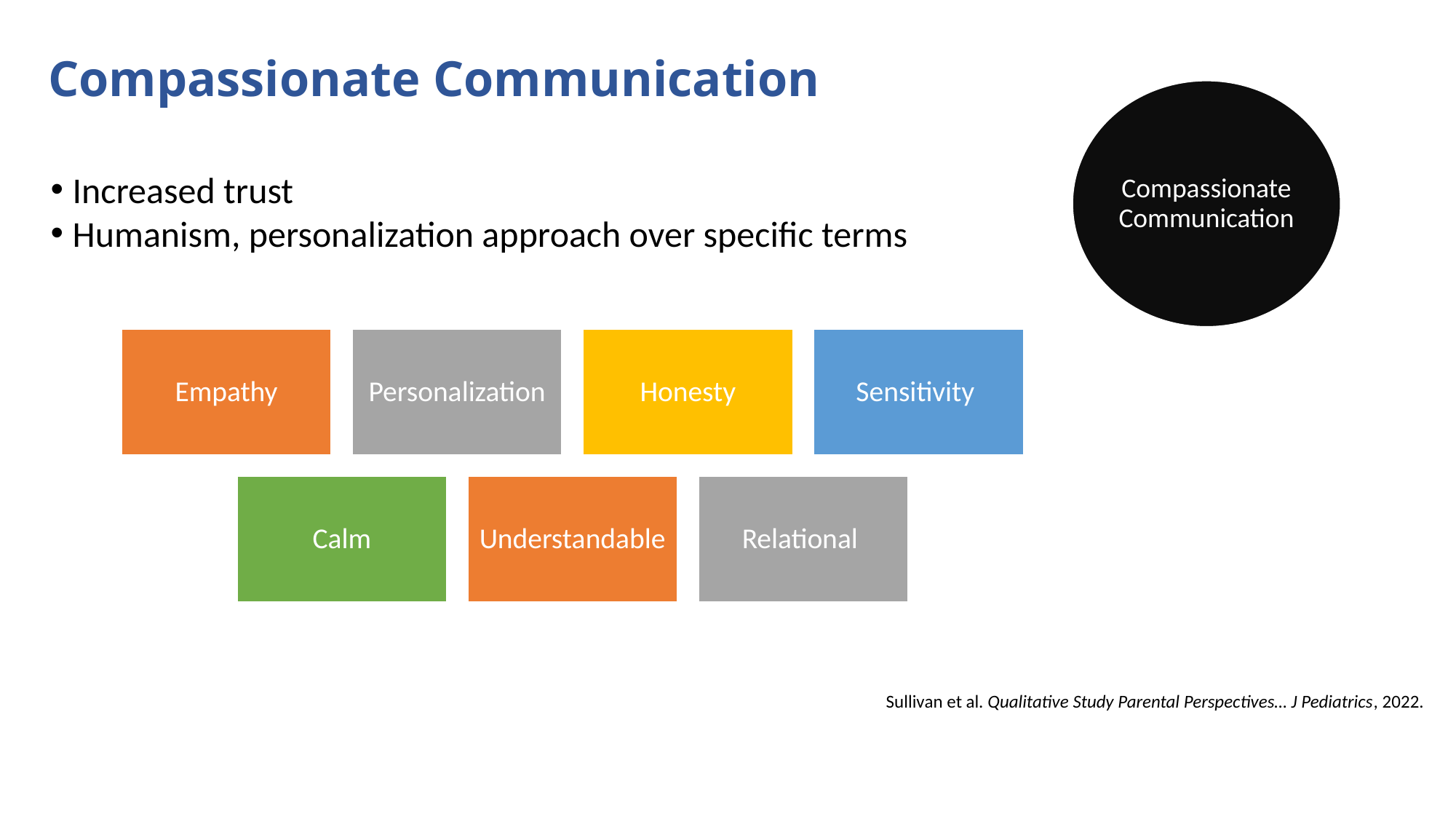

# Compassionate Communication
Compassionate Communication
Increased trust
Humanism, personalization approach over specific terms
Sullivan et al. Qualitative Study Parental Perspectives… J Pediatrics, 2022.

## Slide 28
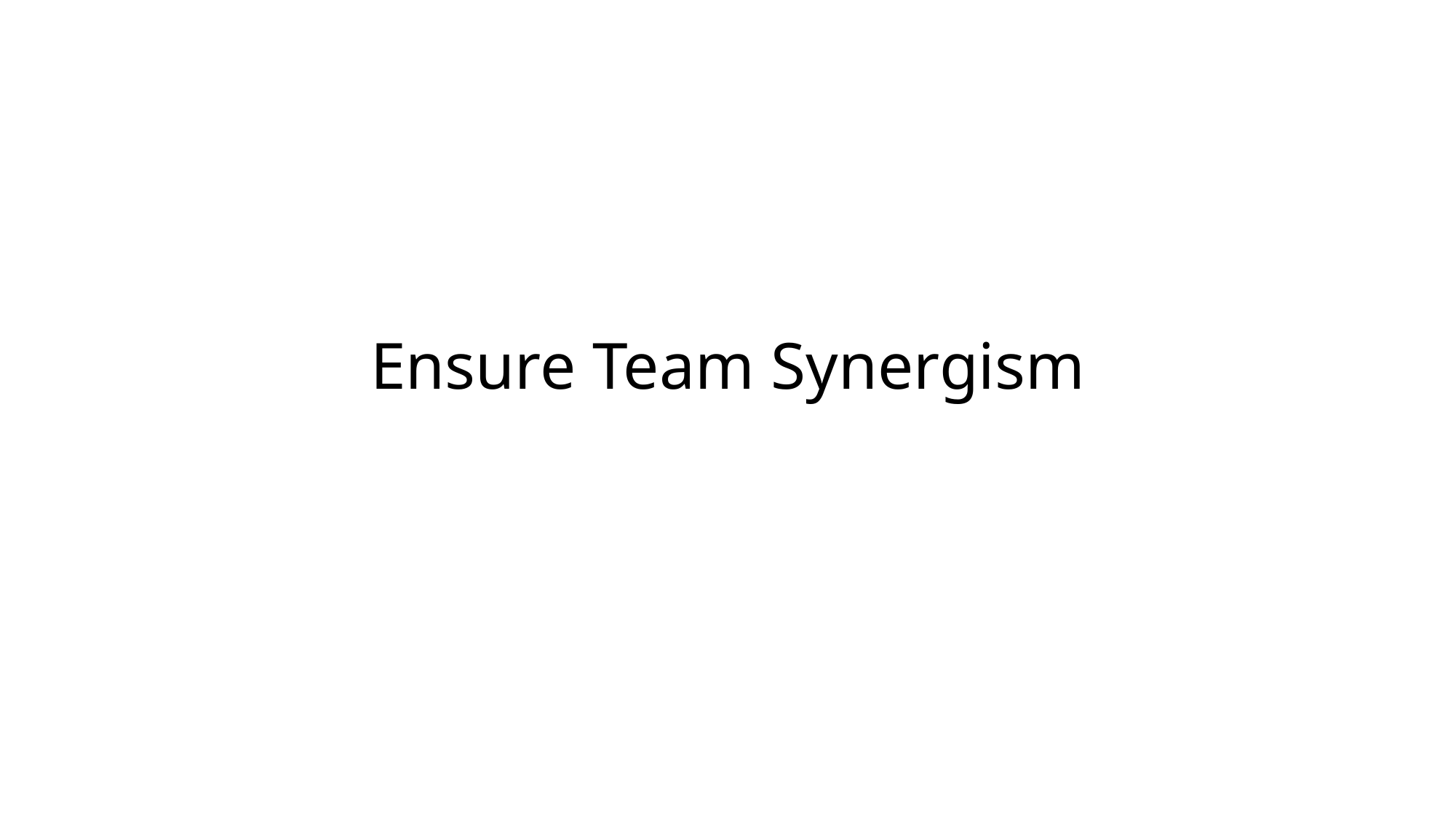

# Ensure Team Synergism

## Slide 29
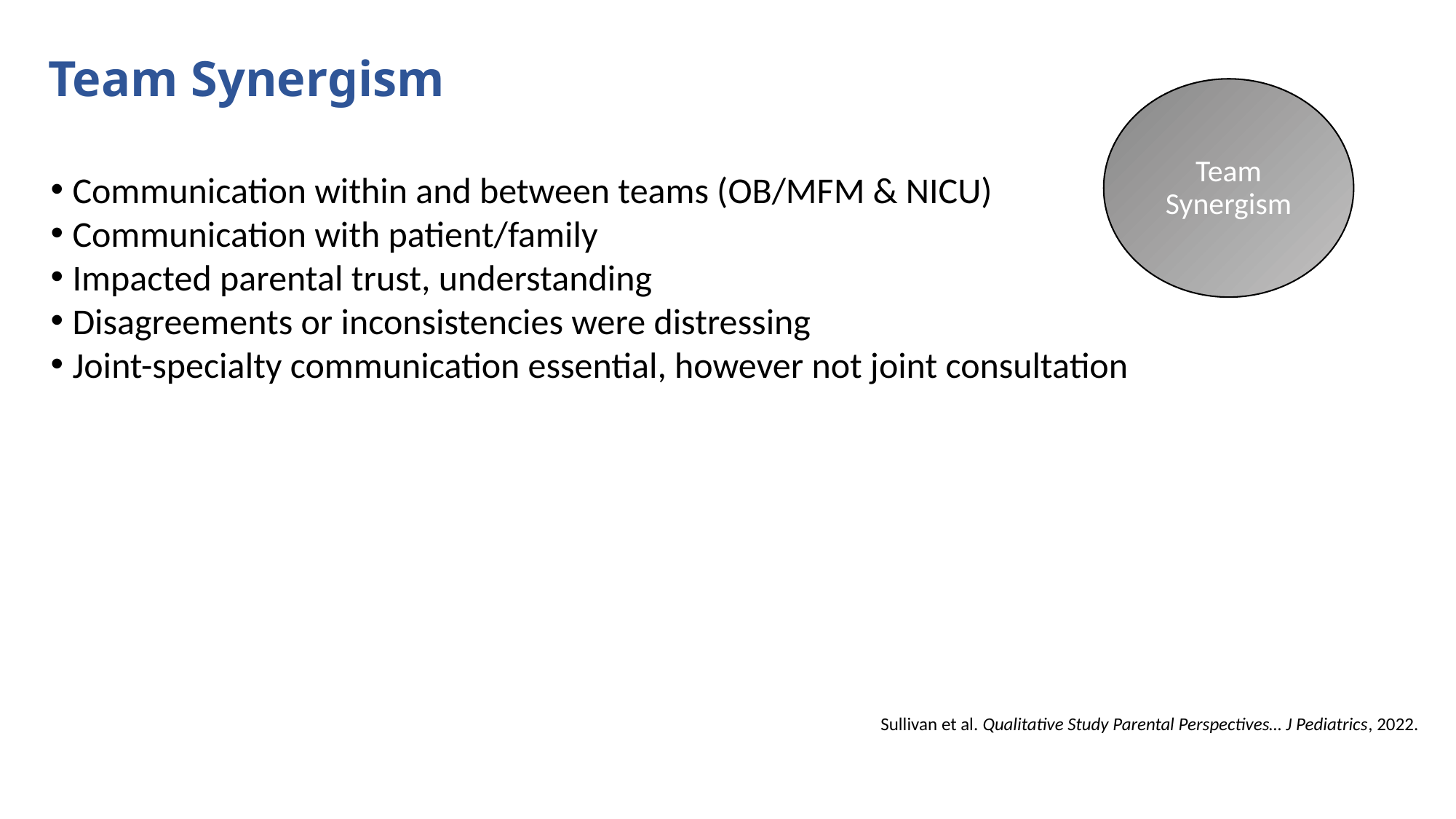

# Team Synergism
Team Synergism
Communication within and between teams (OB/MFM & NICU)
Communication with patient/family
Impacted parental trust, understanding
Disagreements or inconsistencies were distressing
Joint-specialty communication essential, however not joint consultation
Sullivan et al. Qualitative Study Parental Perspectives… J Pediatrics, 2022.

## Slide 30
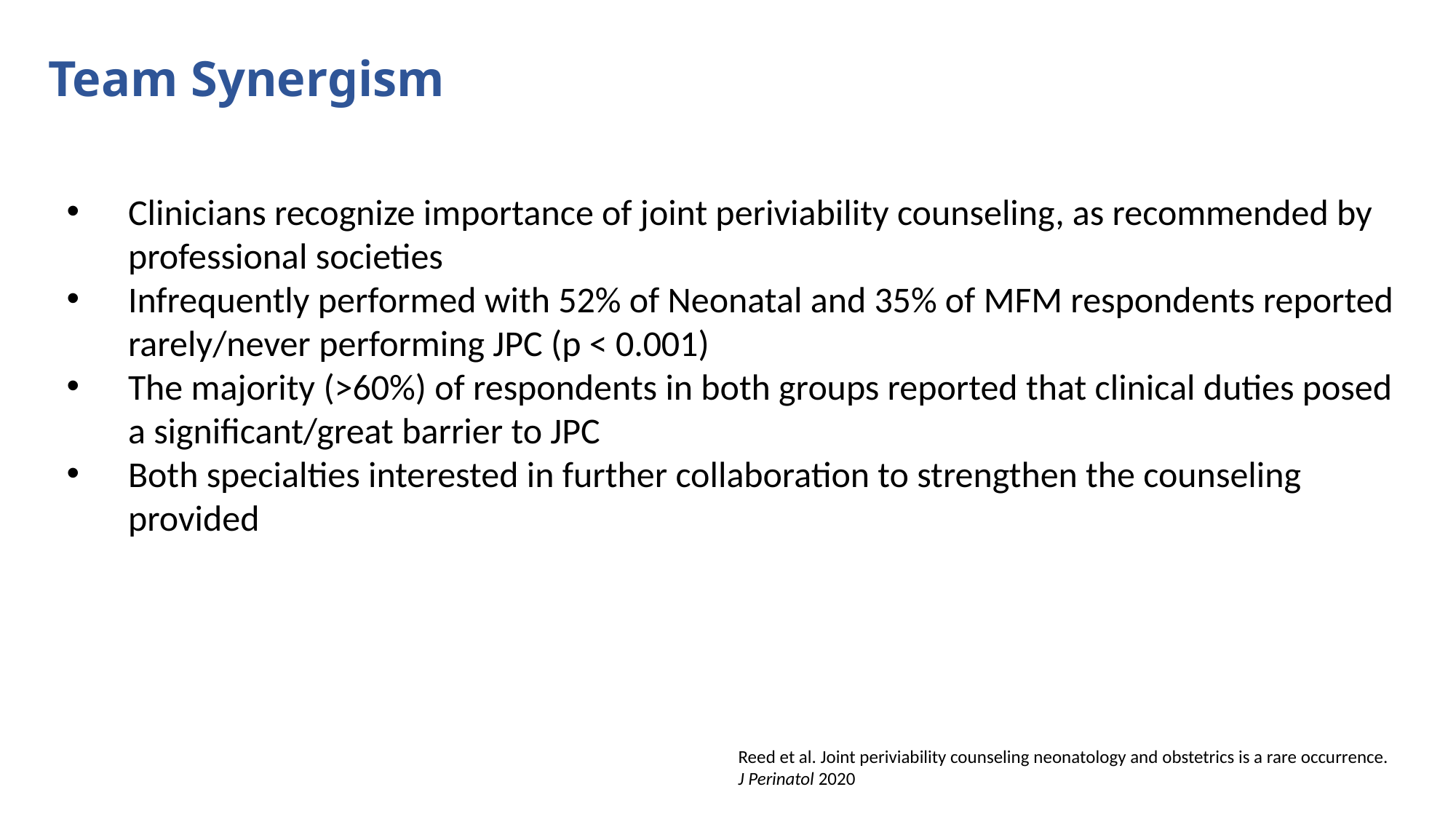

# Team Synergism
Clinicians recognize importance of joint periviability counseling, as recommended by professional societies
Infrequently performed with 52% of Neonatal and 35% of MFM respondents reported rarely/never performing JPC (p < 0.001)
The majority (>60%) of respondents in both groups reported that clinical duties posed a significant/great barrier to JPC
Both specialties interested in further collaboration to strengthen the counseling provided
Reed et al. Joint periviability counseling neonatology and obstetrics is a rare occurrence.
J Perinatol 2020

## Slide 31
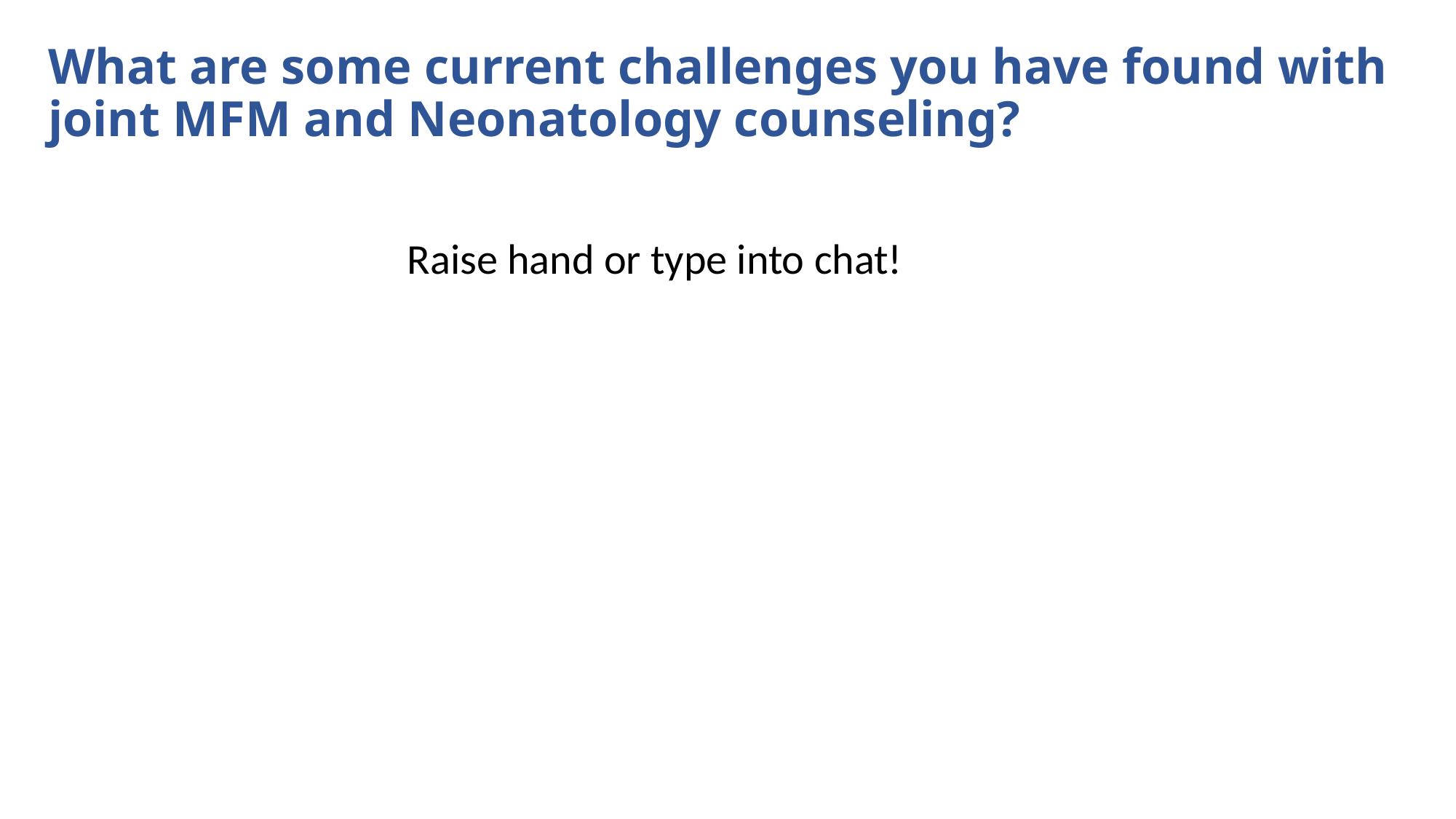

# What are some current challenges you have found with joint MFM and Neonatology counseling?
Raise hand or type into chat!

## Slide 32
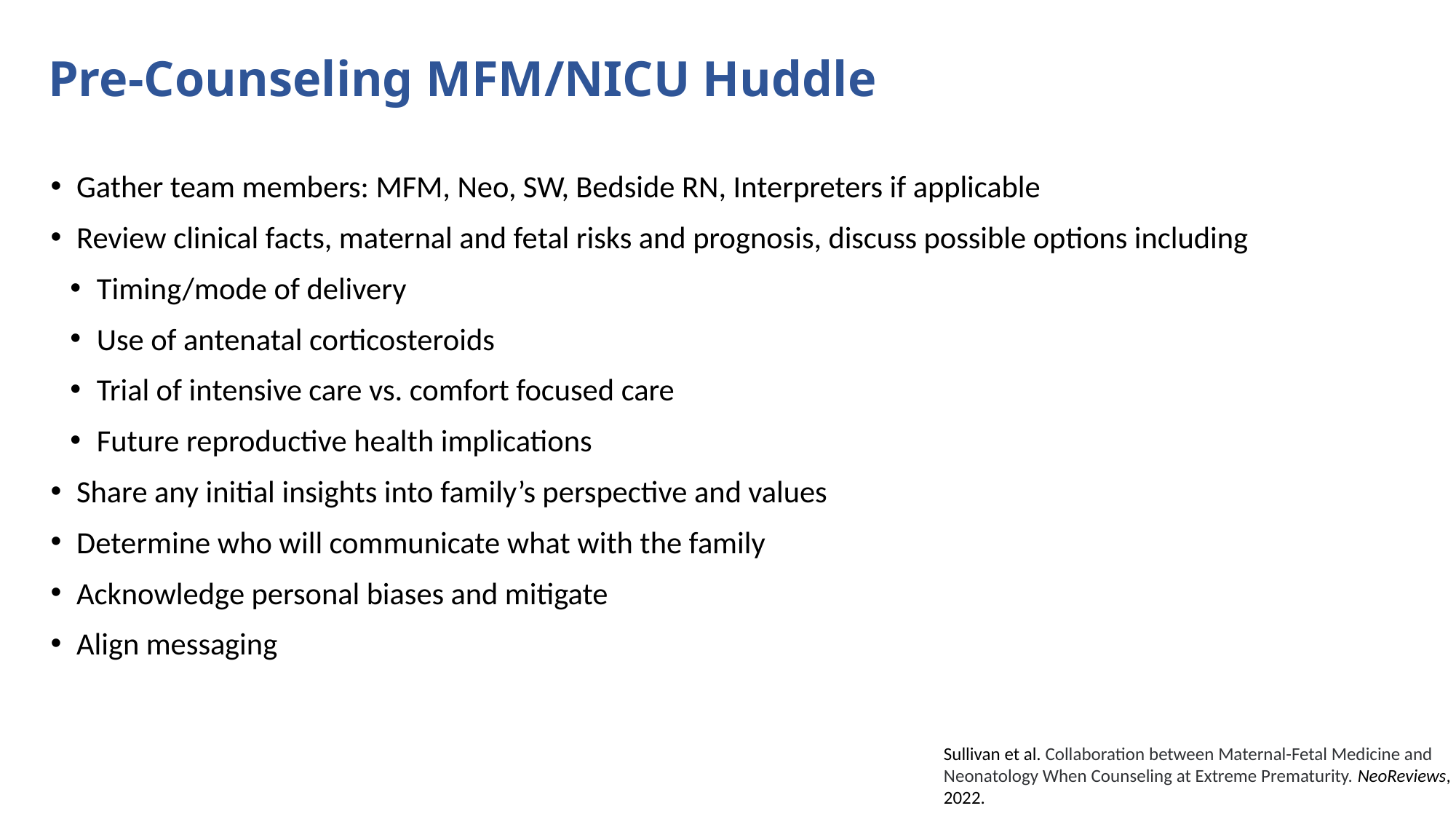

# Pre-Counseling MFM/NICU Huddle
Gather team members: MFM, Neo, SW, Bedside RN, Interpreters if applicable
Review clinical facts, maternal and fetal risks and prognosis, discuss possible options including
Timing/mode of delivery
Use of antenatal corticosteroids
Trial of intensive care vs. comfort focused care
Future reproductive health implications
Share any initial insights into family’s perspective and values
Determine who will communicate what with the family
Acknowledge personal biases and mitigate
Align messaging
Sullivan et al. Collaboration between Maternal-Fetal Medicine and Neonatology When Counseling at Extreme Prematurity. NeoReviews, 2022.

## Slide 33
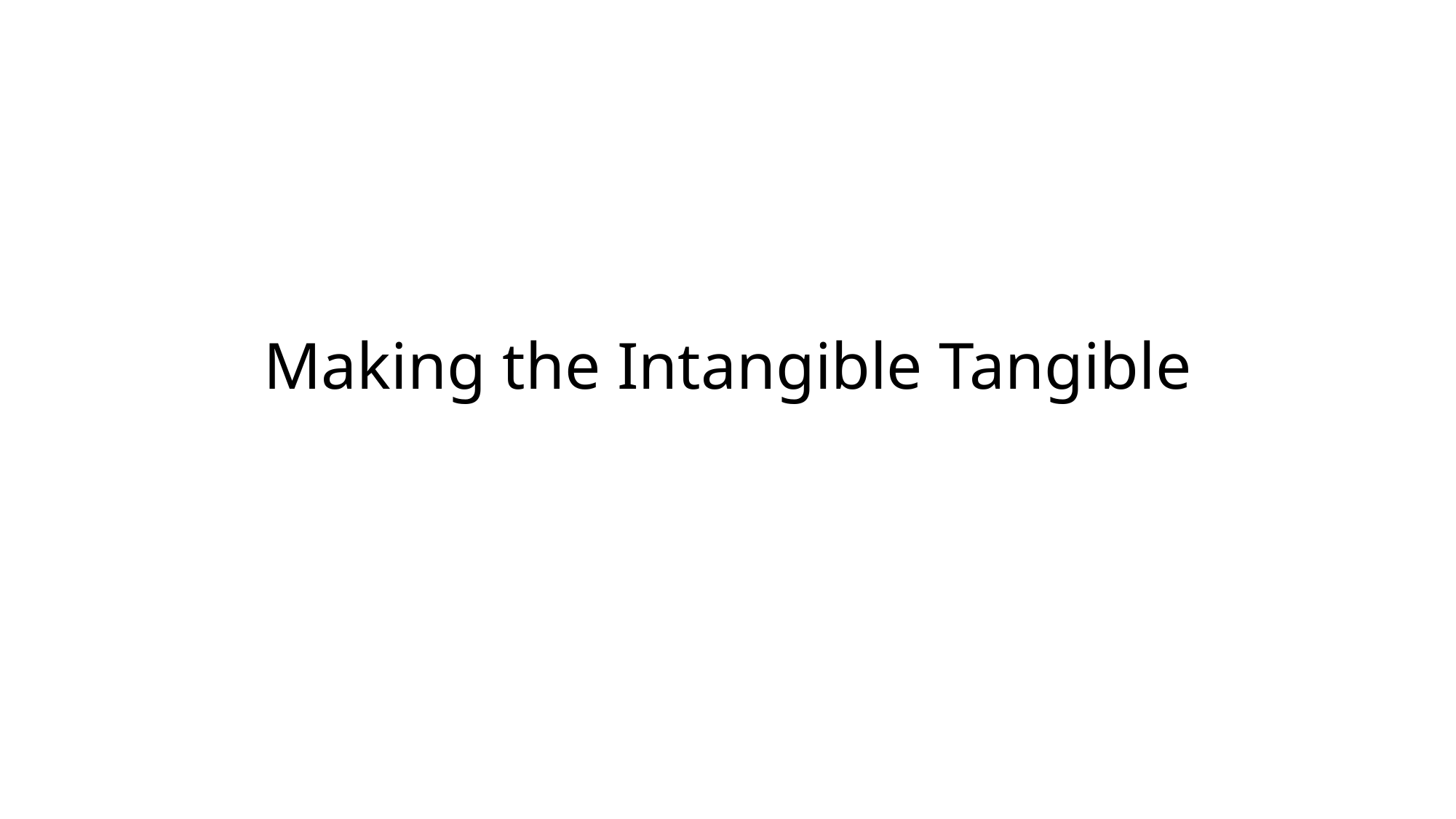

# Making the Intangible Tangible

## Slide 34
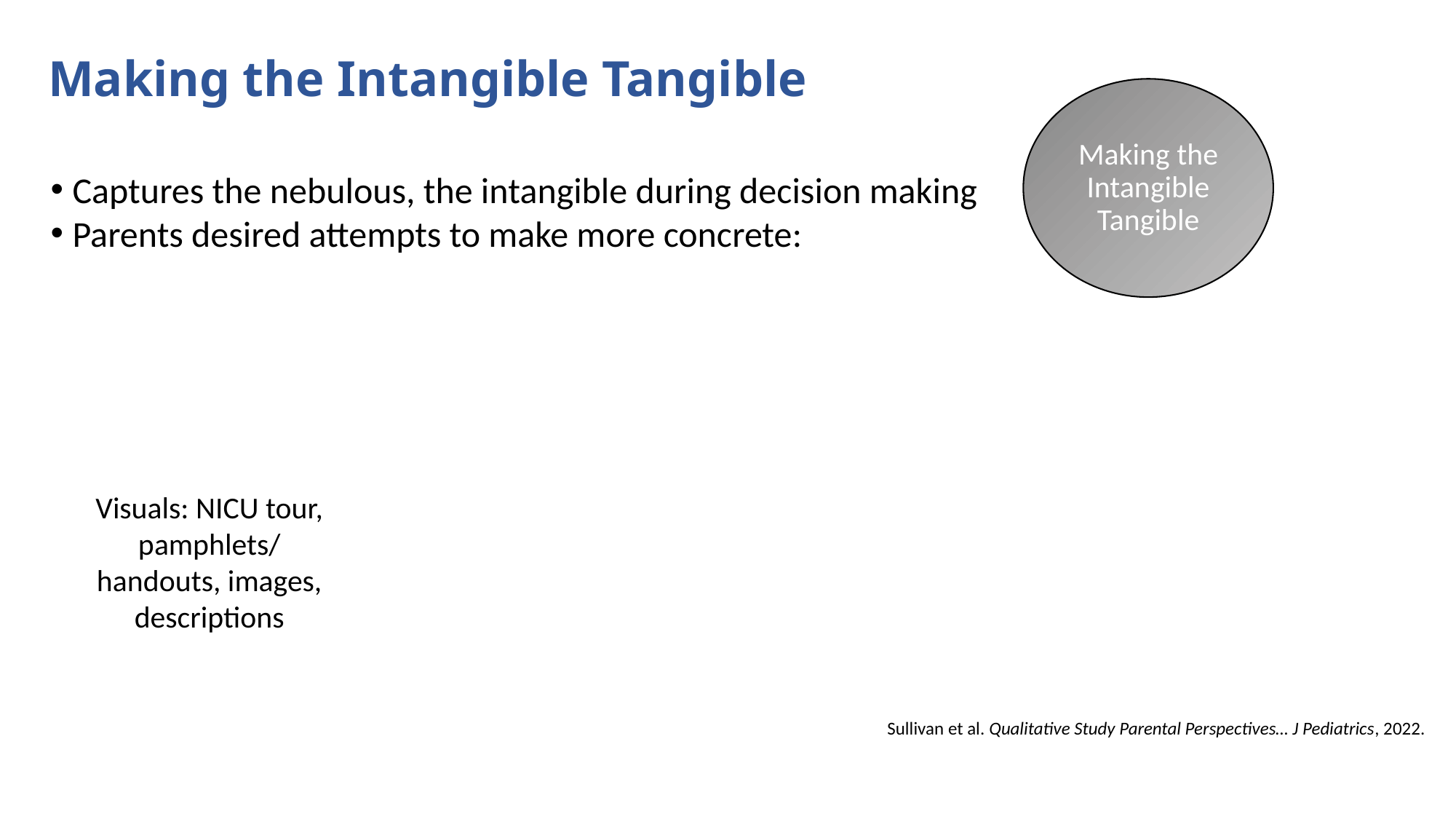

# Making the Intangible Tangible
Making the Intangible Tangible
Captures the nebulous, the intangible during decision making
Parents desired attempts to make more concrete:
Sullivan et al. Qualitative Study Parental Perspectives… J Pediatrics, 2022.

## Slide 35
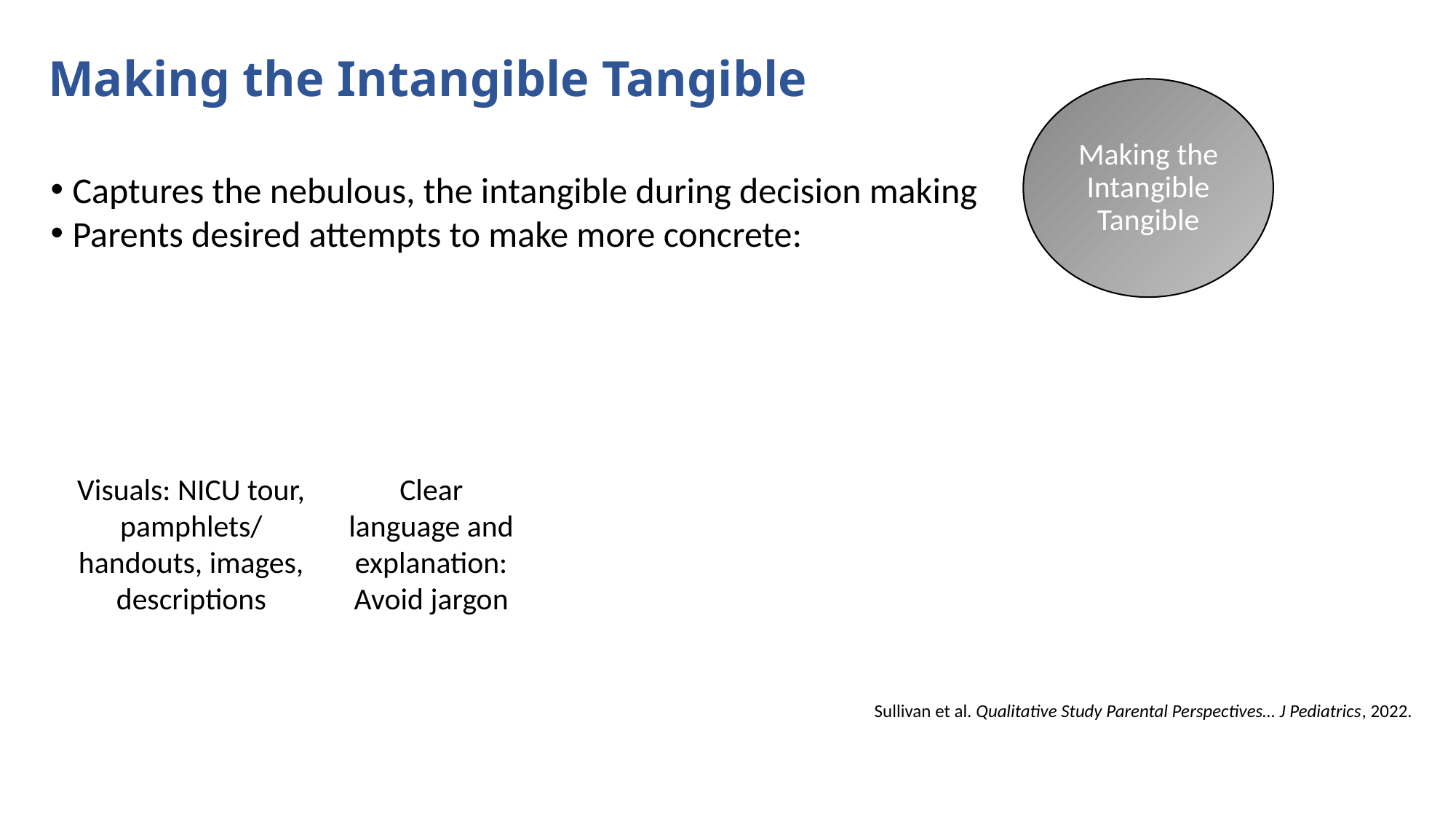

# Making the Intangible Tangible
Making the Intangible Tangible
Captures the nebulous, the intangible during decision making
Parents desired attempts to make more concrete:
Sullivan et al. Qualitative Study Parental Perspectives… J Pediatrics, 2022.

## Slide 36
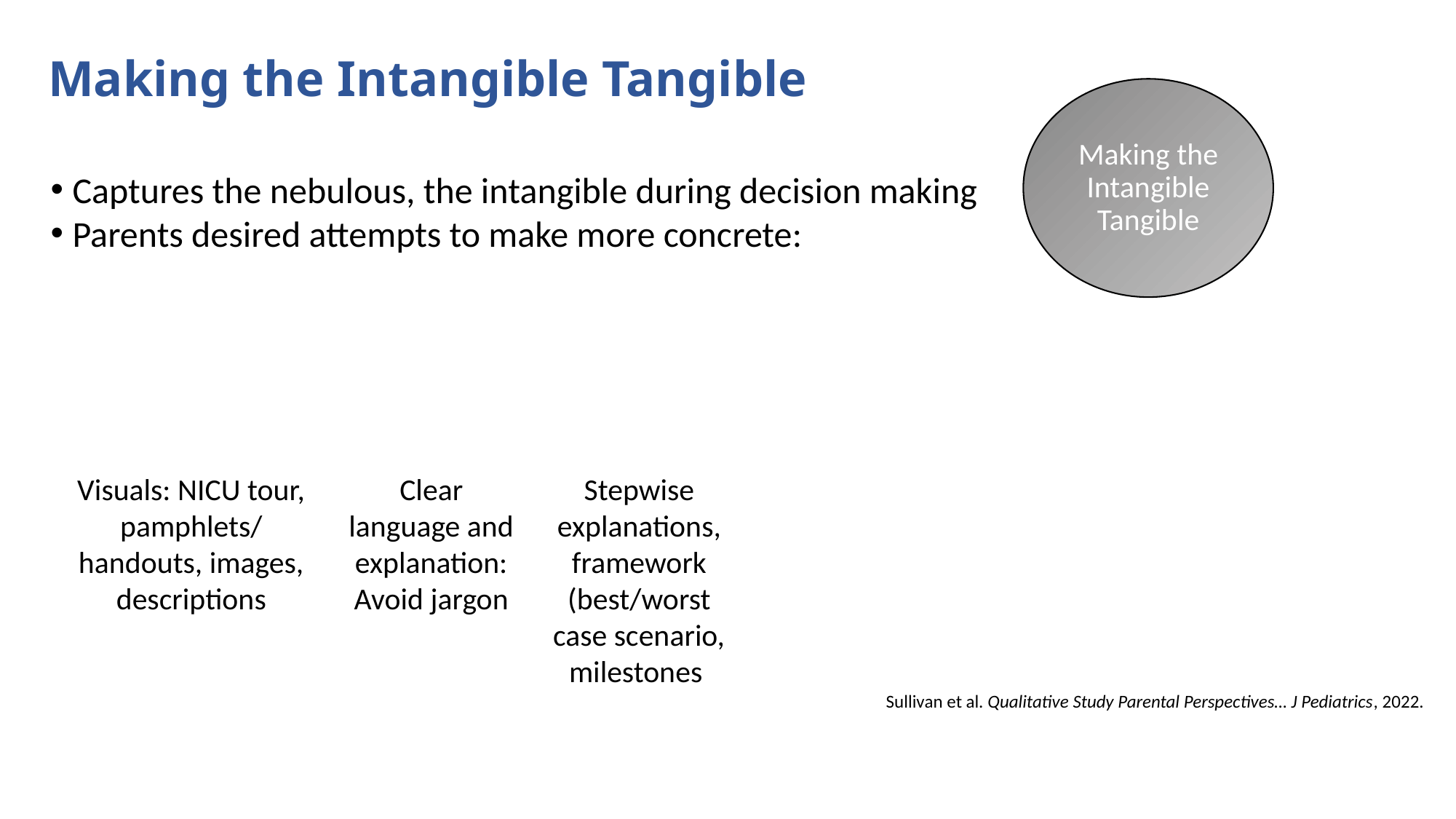

# Making the Intangible Tangible
Making the Intangible Tangible
Captures the nebulous, the intangible during decision making
Parents desired attempts to make more concrete:
Sullivan et al. Qualitative Study Parental Perspectives… J Pediatrics, 2022.

## Slide 37
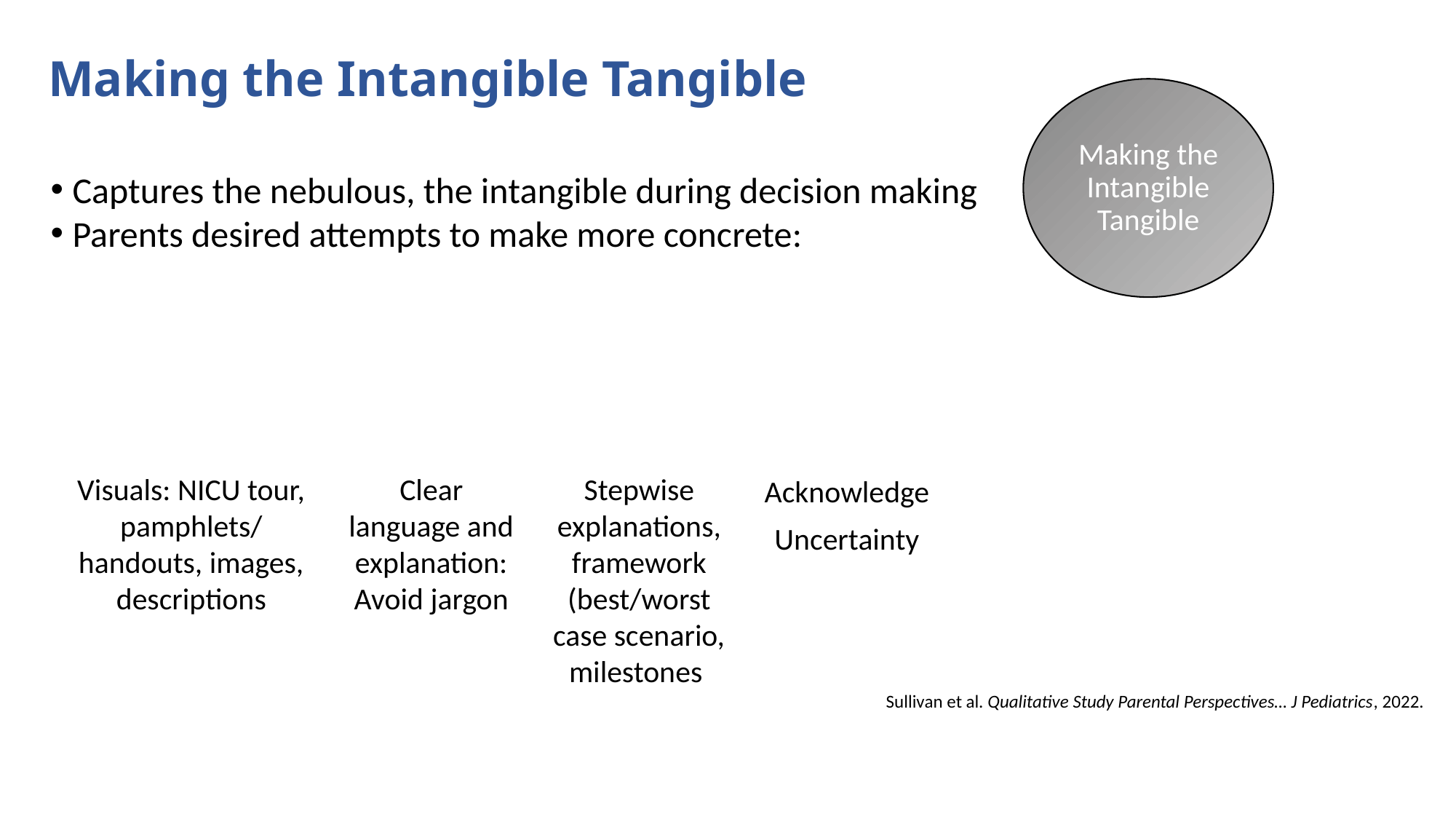

# Making the Intangible Tangible
Making the Intangible Tangible
Captures the nebulous, the intangible during decision making
Parents desired attempts to make more concrete:
Sullivan et al. Qualitative Study Parental Perspectives… J Pediatrics, 2022.

## Slide 38
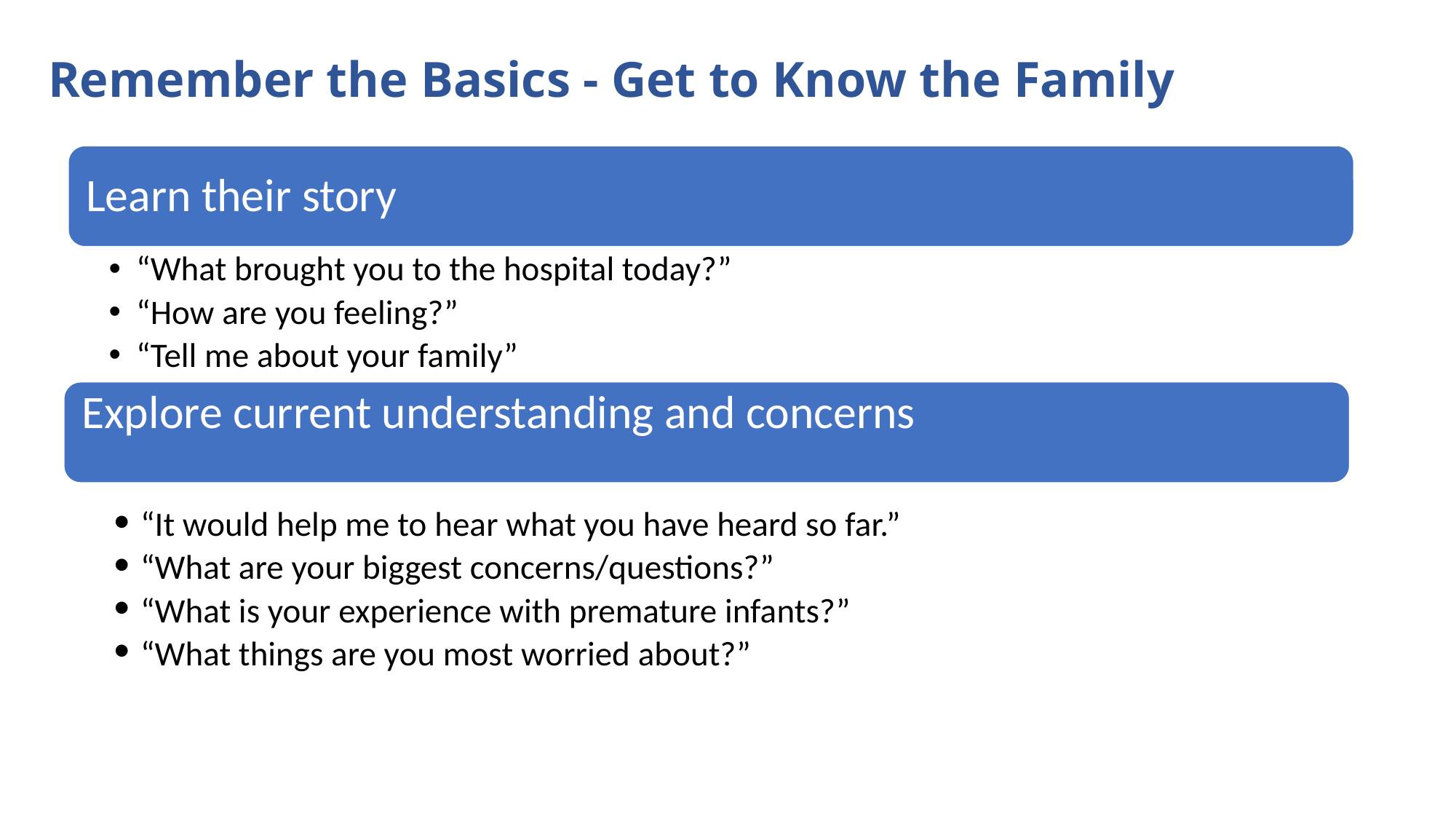

# Remember the Basics - Get to Know the Family
Learn their story
“What brought you to the hospital today?”
“How are you feeling?”
“Tell me about your family”
Explore current understanding and concerns
“It would help me to hear what you have heard so far.”
“What are your biggest concerns/questions?”
“What is your experience with premature infants?”
“What things are you most worried about?”

## Slide 39
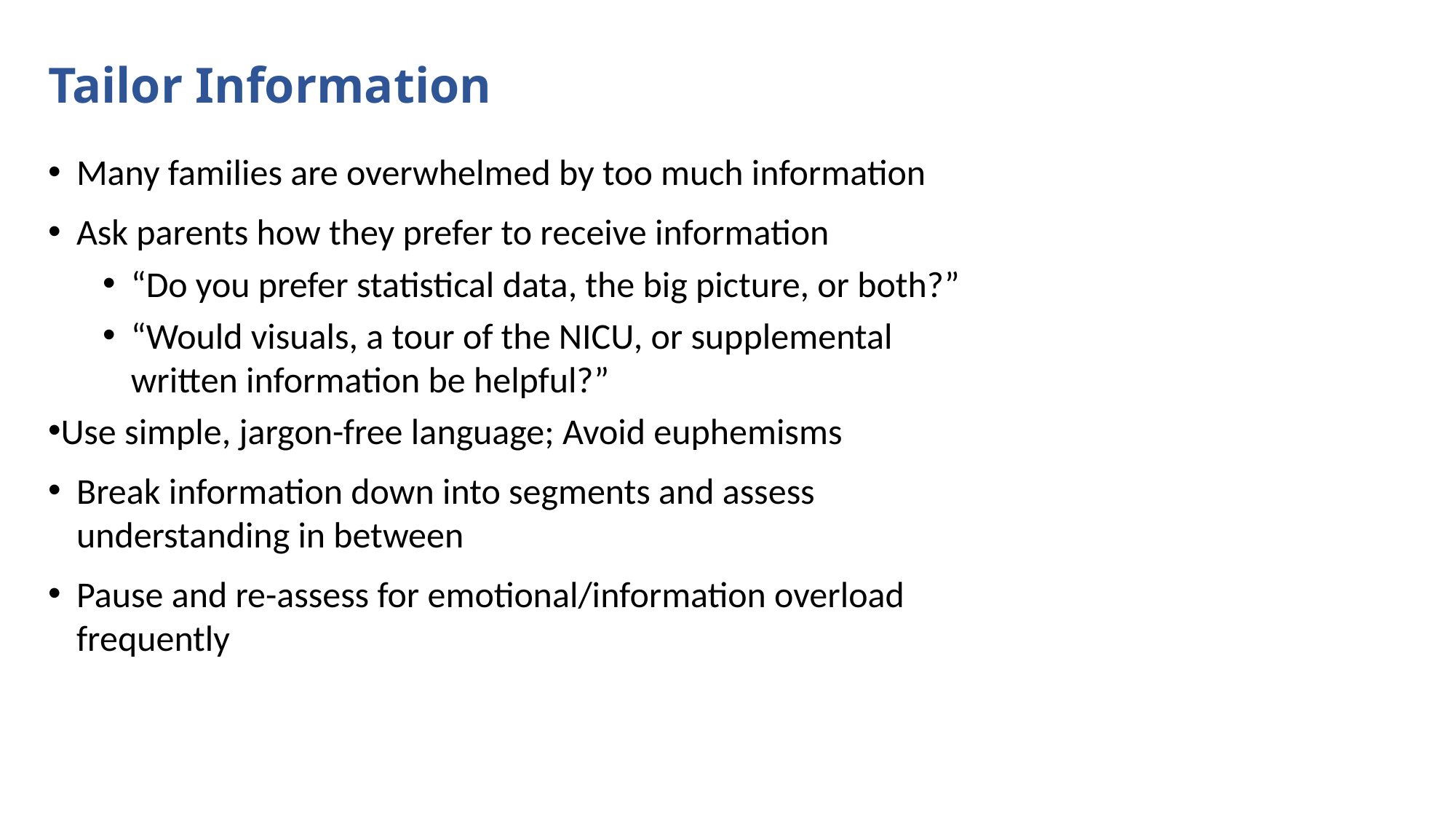

# Tailor Information
Many families are overwhelmed by too much information
Ask parents how they prefer to receive information
“Do you prefer statistical data, the big picture, or both?”
“Would visuals, a tour of the NICU, or supplemental written information be helpful?”
Use simple, jargon-free language; Avoid euphemisms
Break information down into segments and assess understanding in between
Pause and re-assess for emotional/information overload frequently

## Slide 40
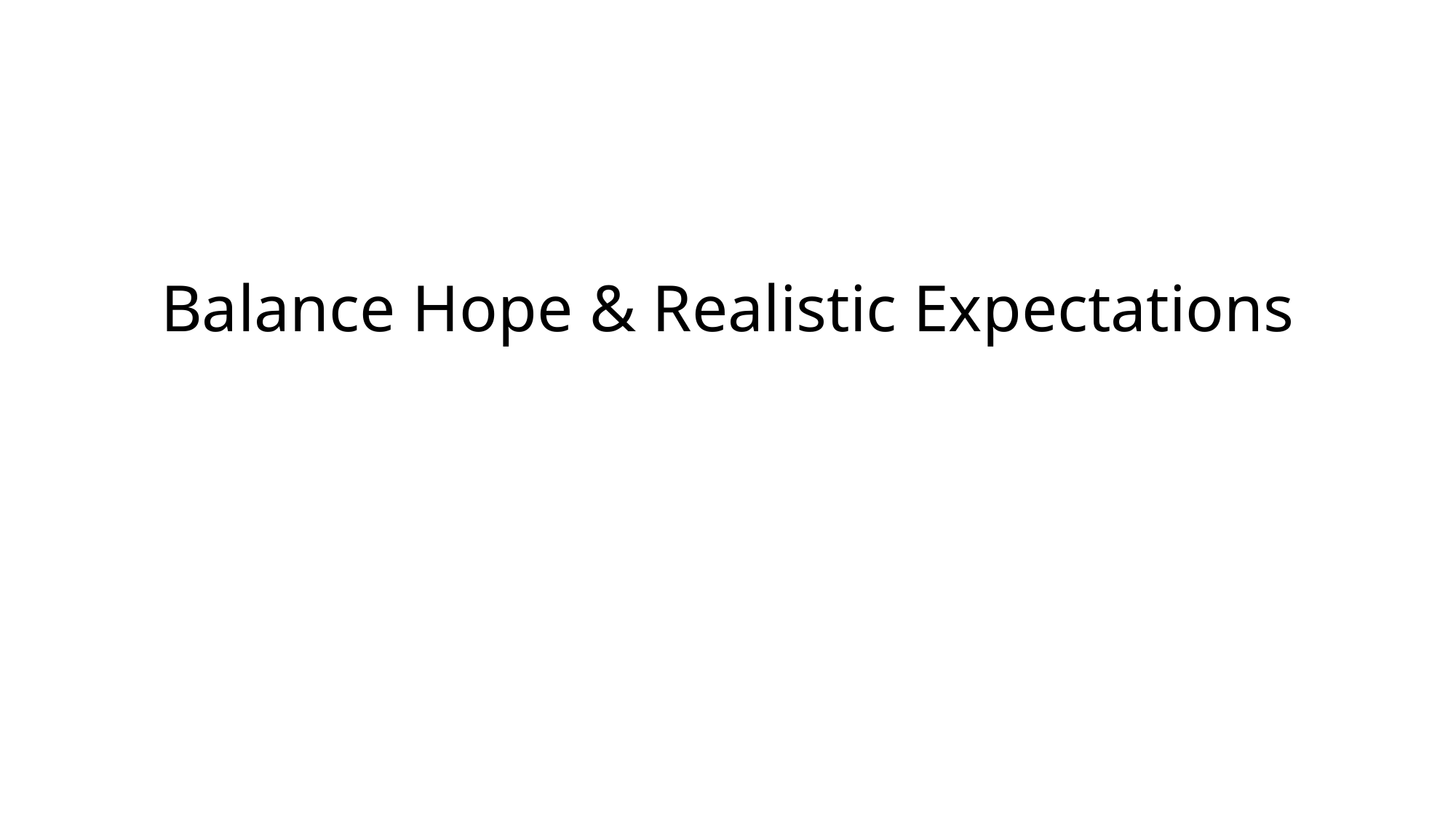

# Balance Hope & Realistic Expectations

## Slide 41
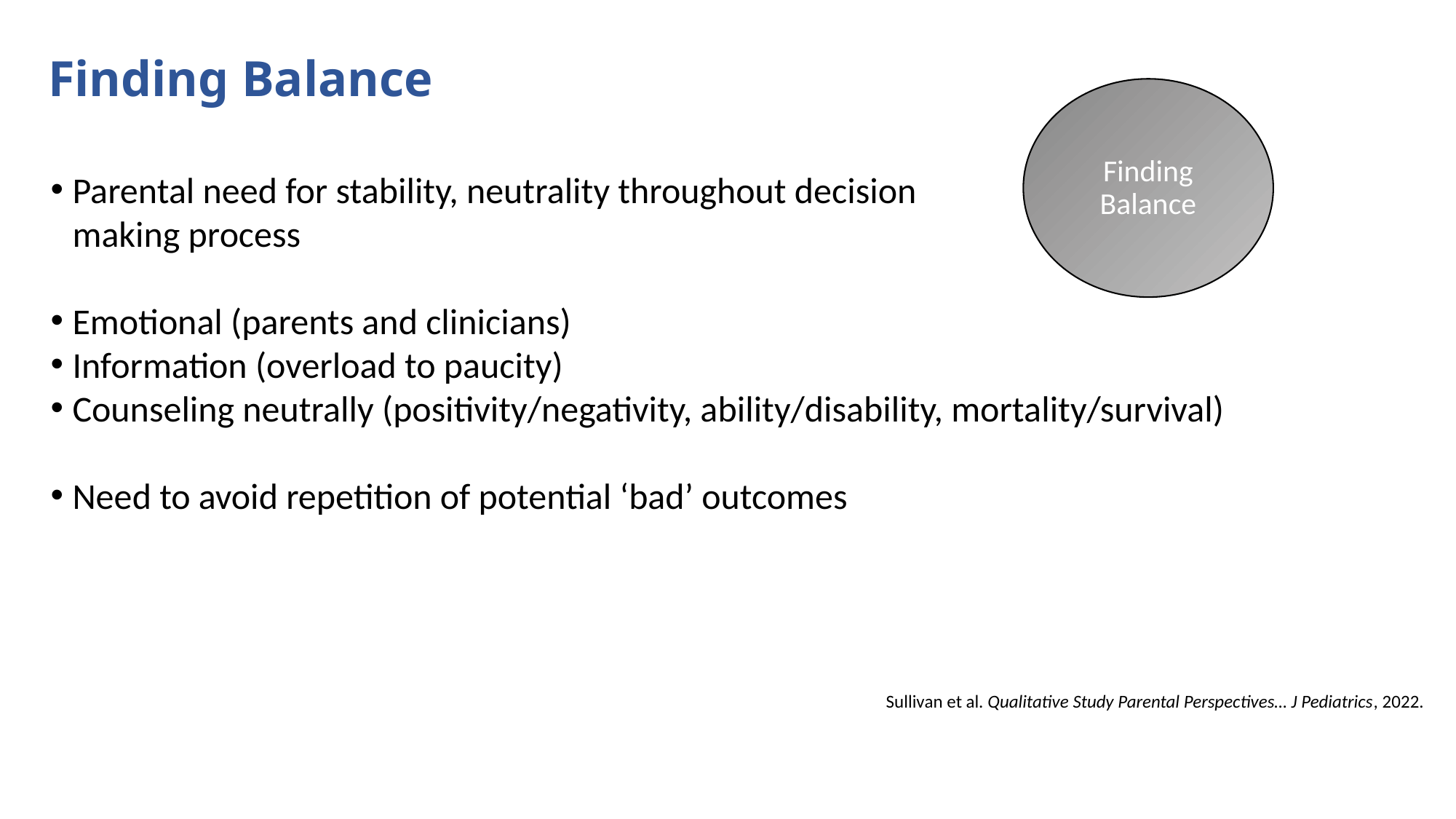

# Finding Balance
Finding Balance
Parental need for stability, neutrality throughout decision
making process
Emotional (parents and clinicians)
Information (overload to paucity)
Counseling neutrally (positivity/negativity, ability/disability, mortality/survival)
Need to avoid repetition of potential ‘bad’ outcomes
Sullivan et al. Qualitative Study Parental Perspectives… J Pediatrics, 2022.

## Slide 42
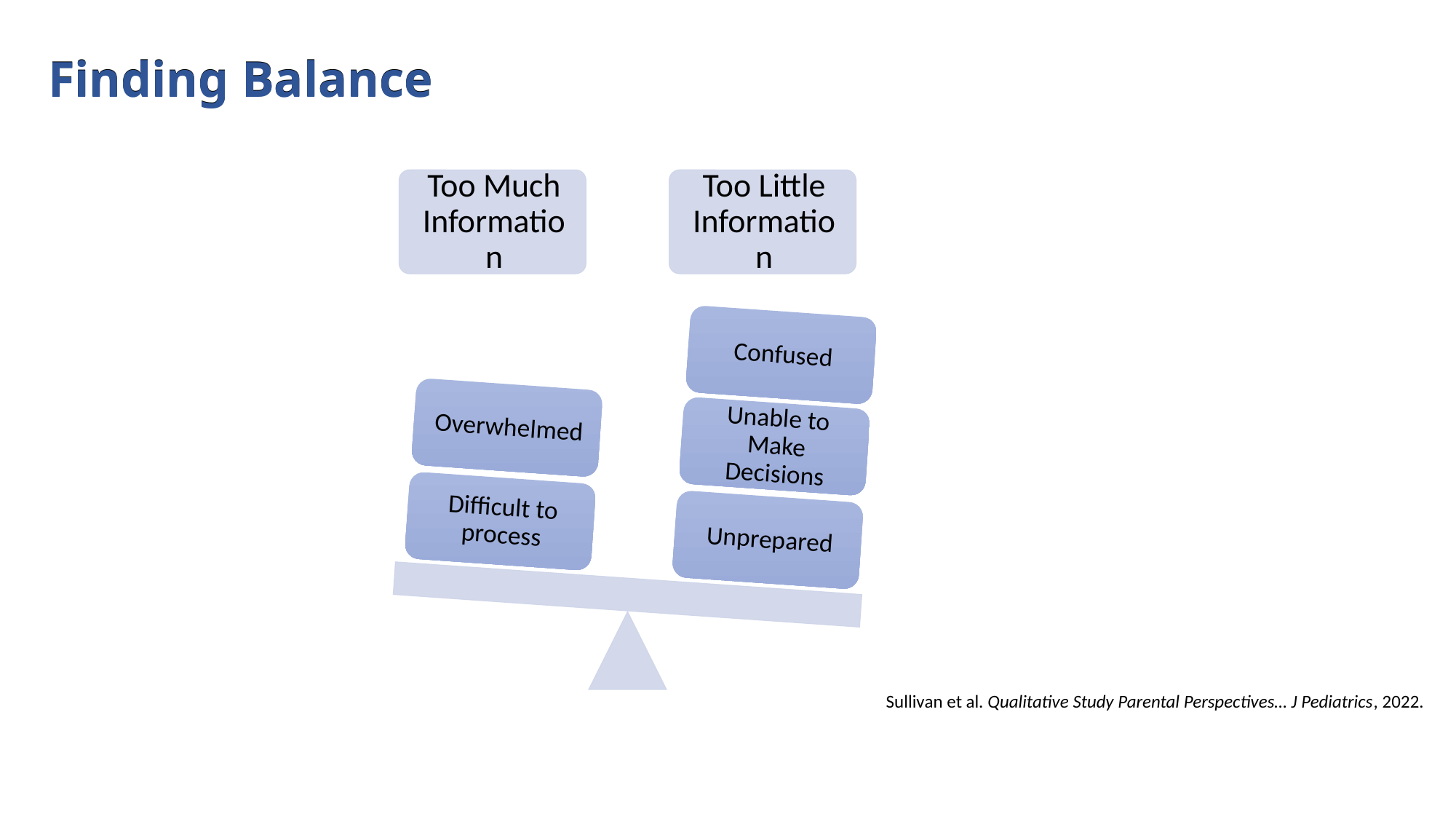

# Finding Balance
Finding Balance
Sullivan et al. Qualitative Study Parental Perspectives… J Pediatrics, 2022.

## Slide 43
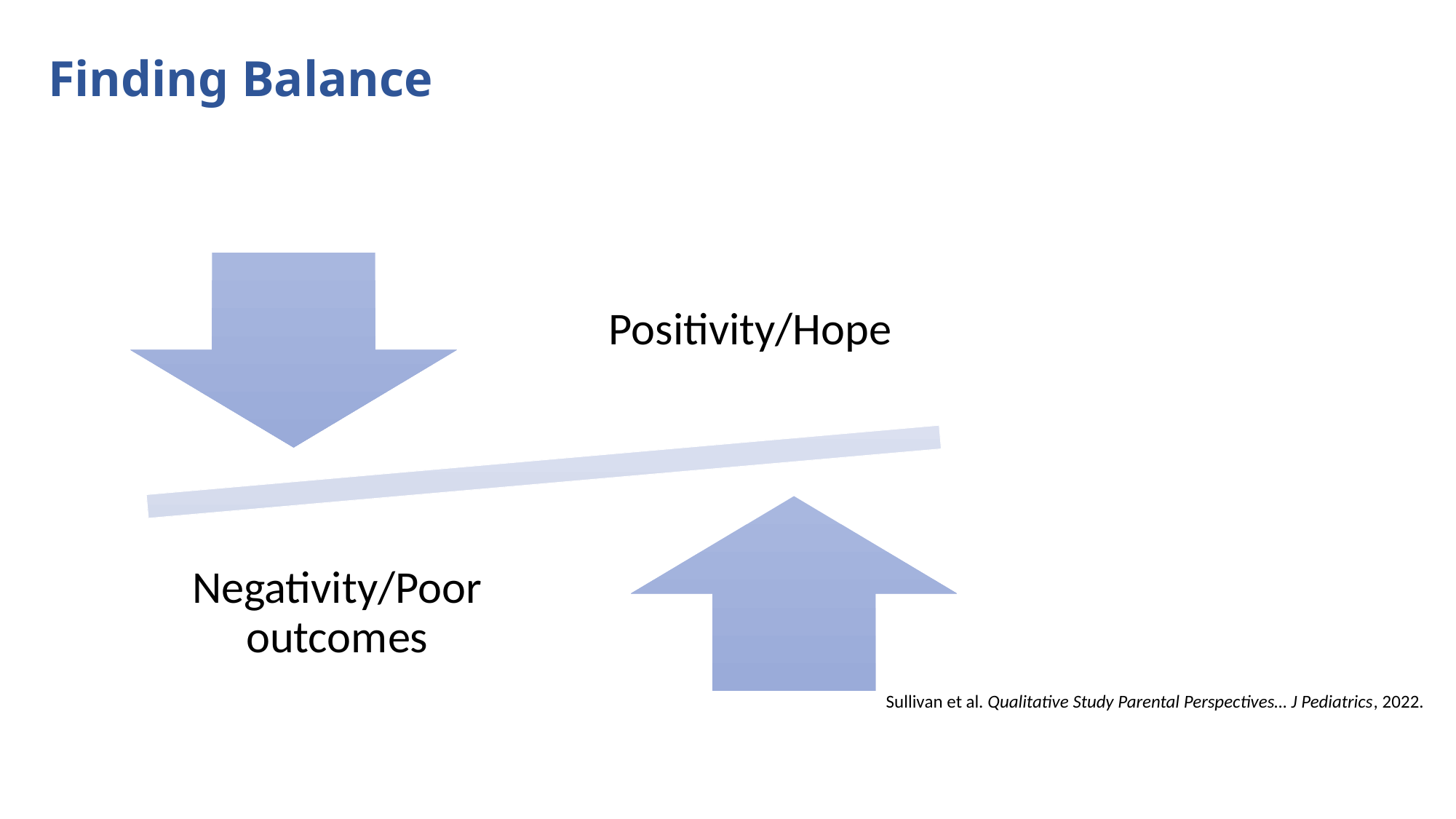

# Finding Balance
Sullivan et al. Qualitative Study Parental Perspectives… J Pediatrics, 2022.

## Slide 44
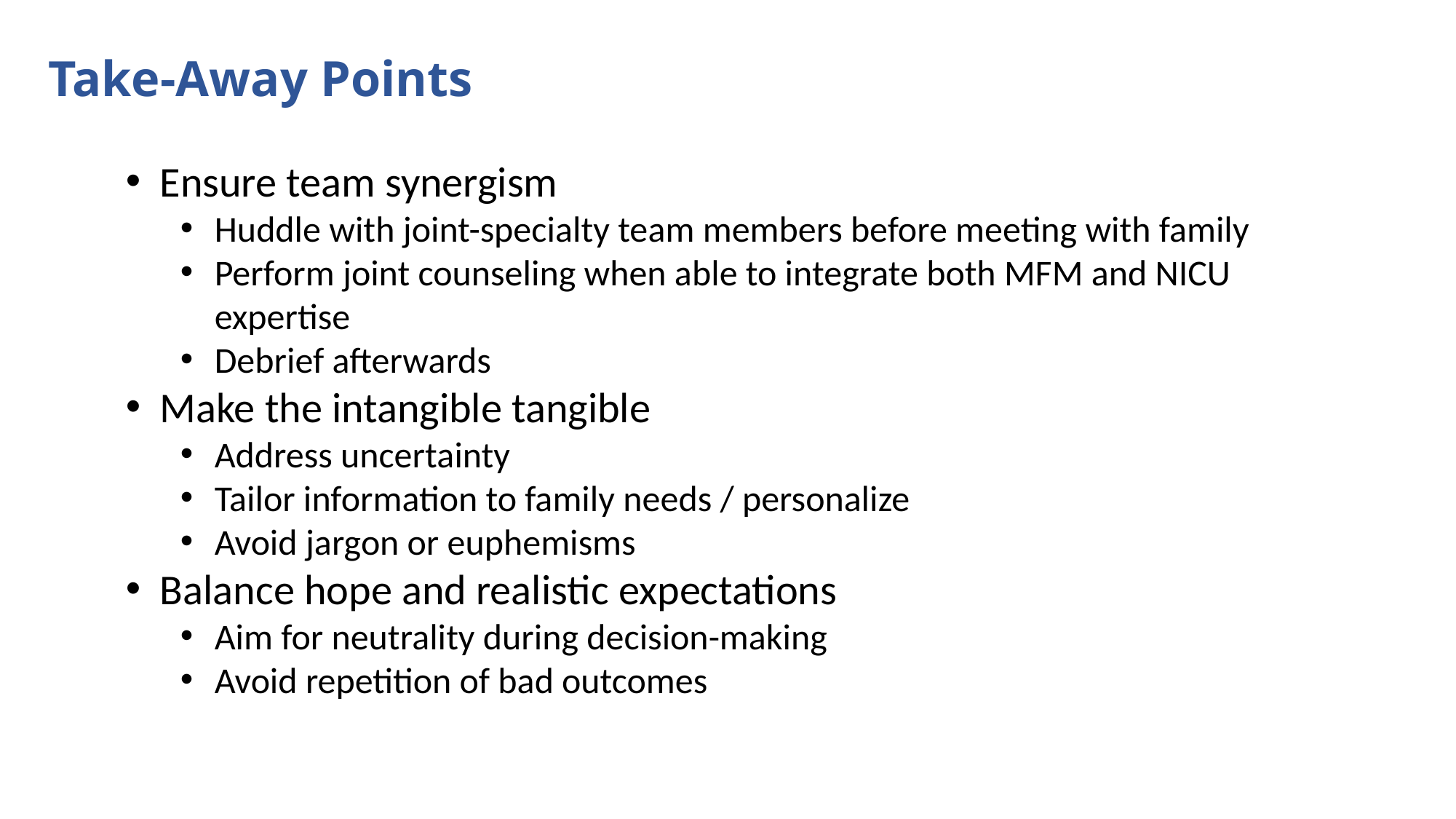

# Take-Away Points
Ensure team synergism
Huddle with joint-specialty team members before meeting with family
Perform joint counseling when able to integrate both MFM and NICU expertise
Debrief afterwards
Make the intangible tangible
Address uncertainty
Tailor information to family needs / personalize
Avoid jargon or euphemisms
Balance hope and realistic expectations
Aim for neutrality during decision-making
Avoid repetition of bad outcomes

## Slide 45
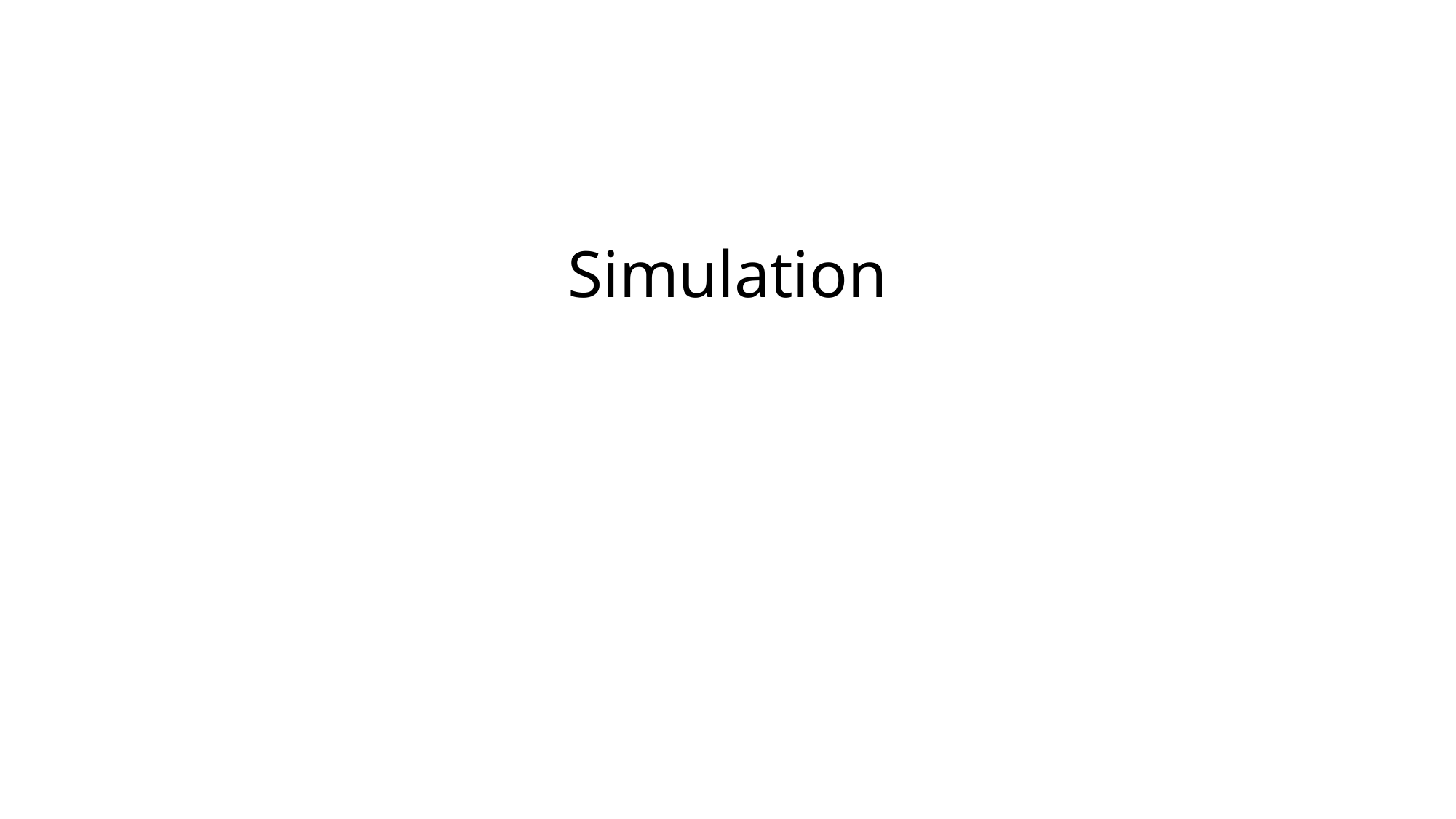

# Simulation

## Slide 46
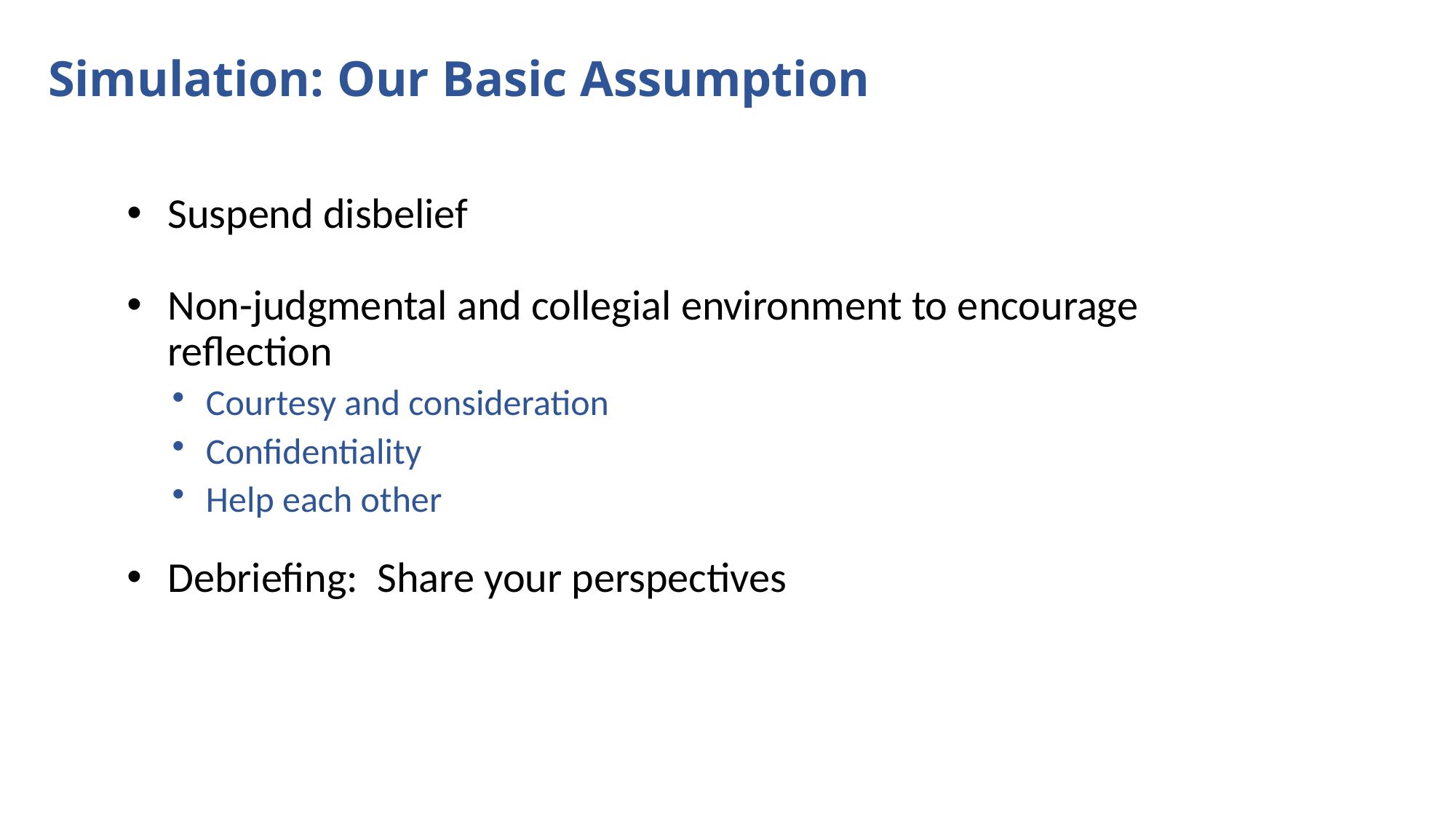

# Simulation: Our Basic Assumption
Suspend disbelief
Non-judgmental and collegial environment to encourage reflection
Courtesy and consideration
Confidentiality
Help each other
Debriefing: Share your perspectives

## Slide 47
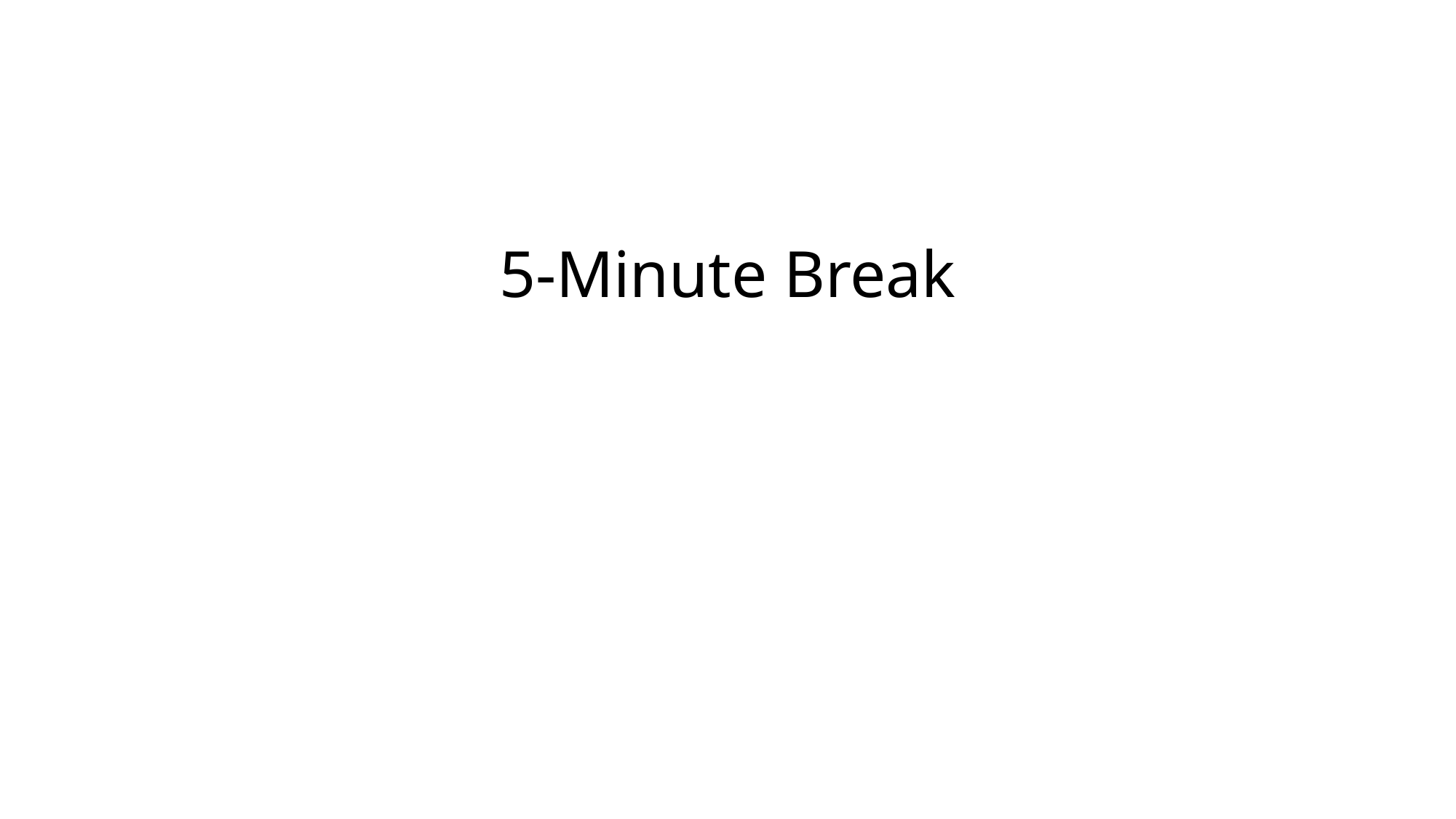

# 5-Minute Break

## Slide 48
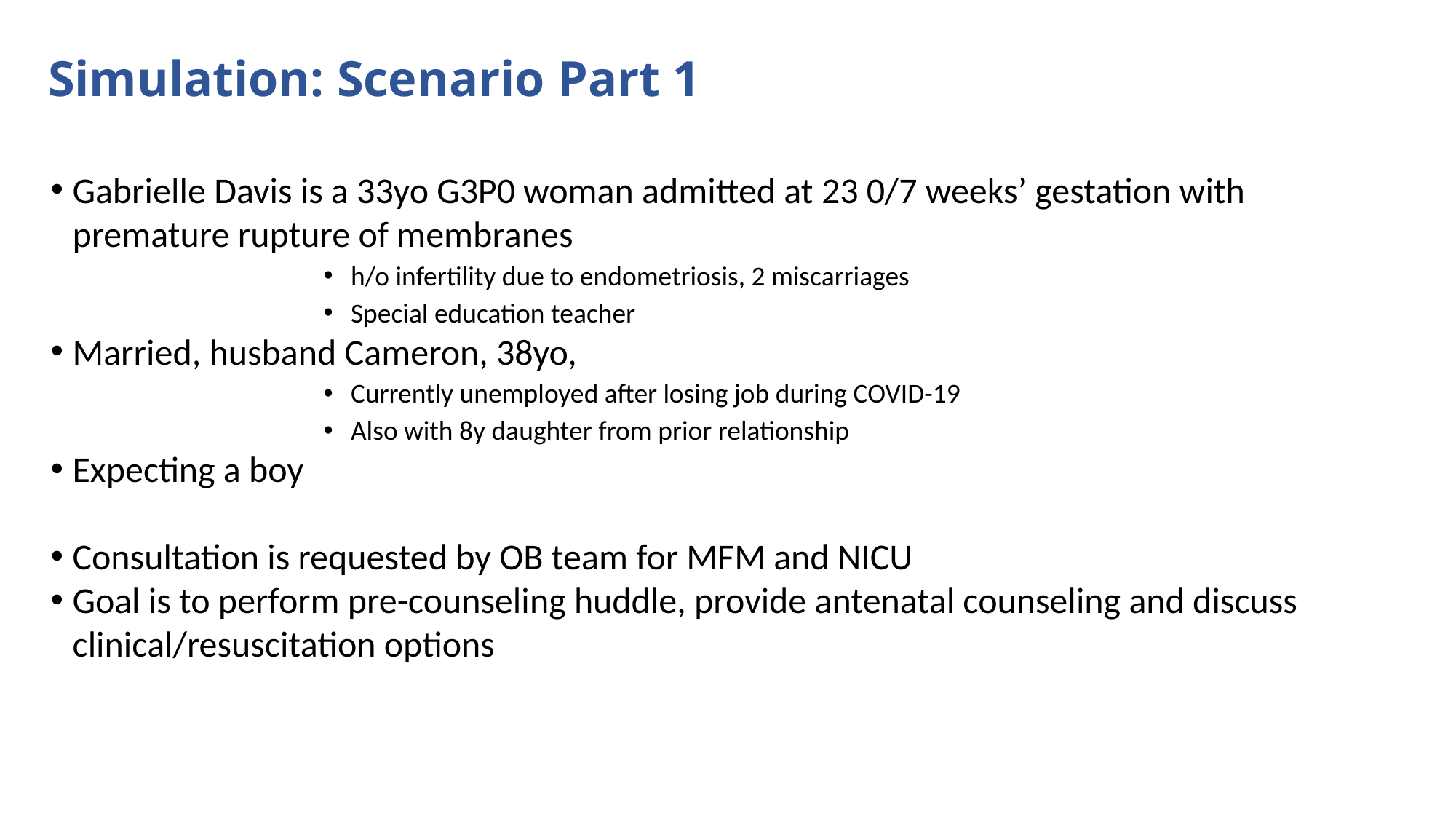

# Simulation: Scenario Part 1
Gabrielle Davis is a 33yo G3P0 woman admitted at 23 0/7 weeks’ gestation with premature rupture of membranes
h/o infertility due to endometriosis, 2 miscarriages
Special education teacher
Married, husband Cameron, 38yo,
Currently unemployed after losing job during COVID-19
Also with 8y daughter from prior relationship
Expecting a boy
Consultation is requested by OB team for MFM and NICU
Goal is to perform pre-counseling huddle, provide antenatal counseling and discuss clinical/resuscitation options

## Slide 49
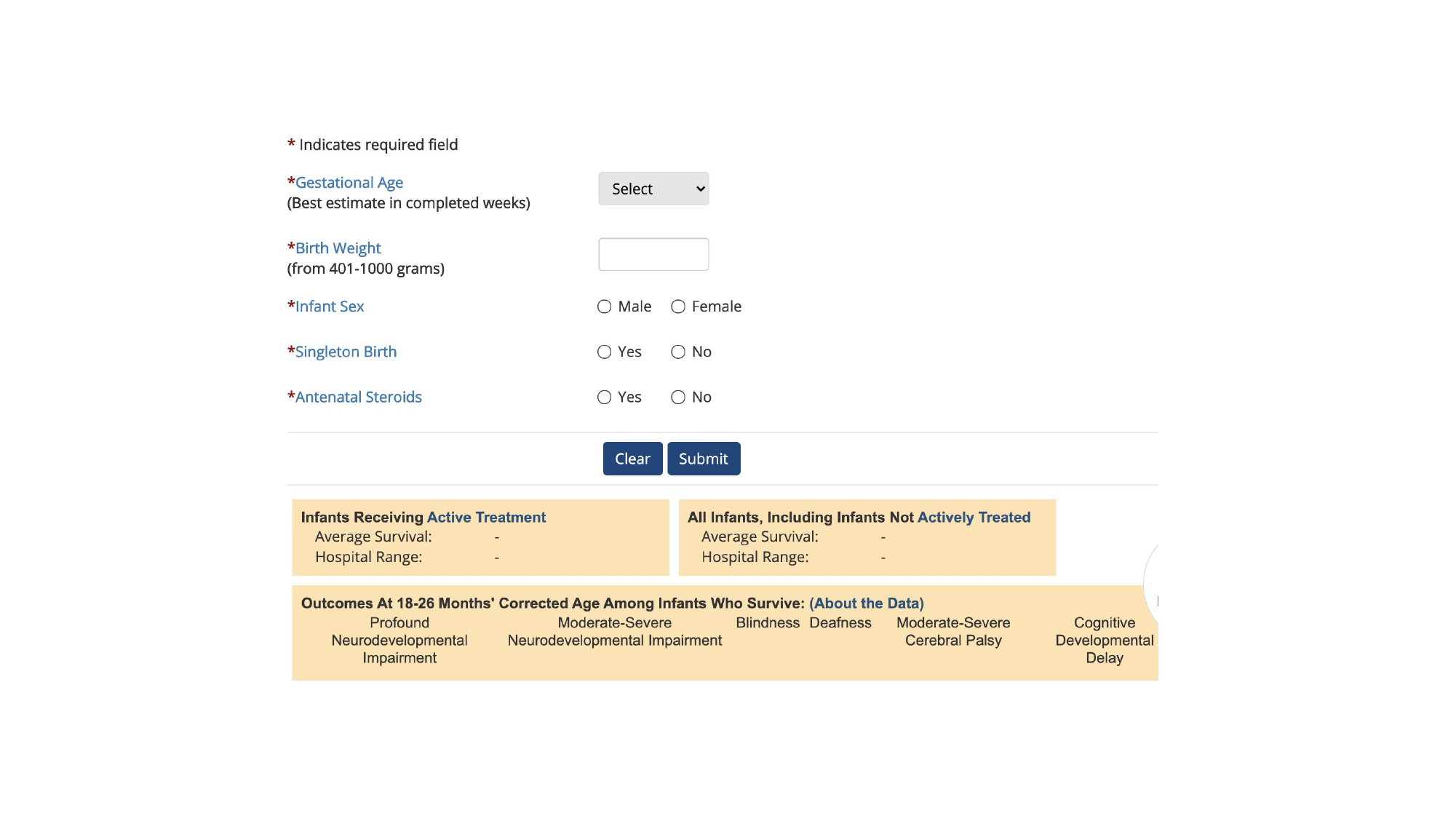

## Slide 50
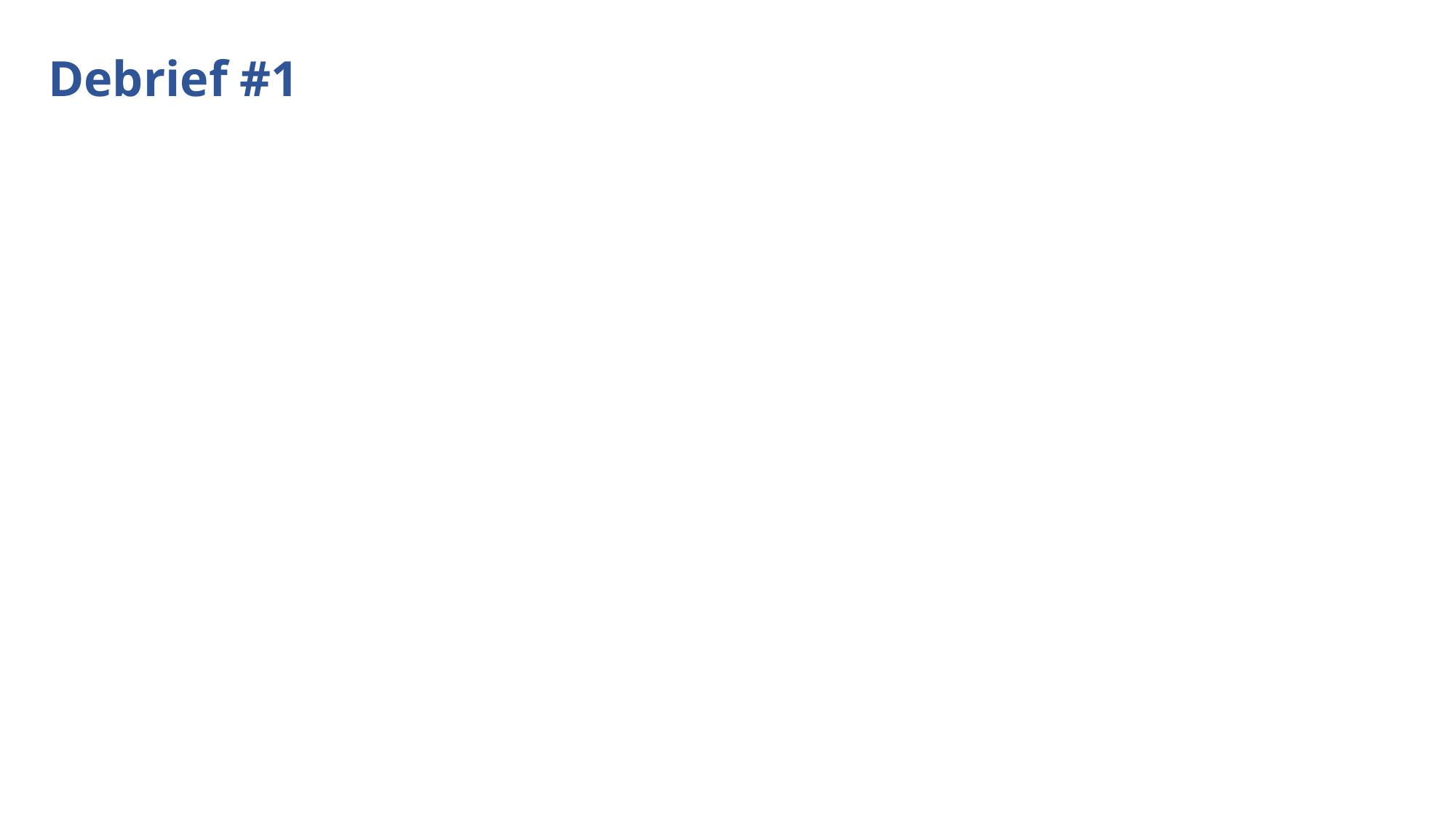

# Debrief #1

## Slide 51
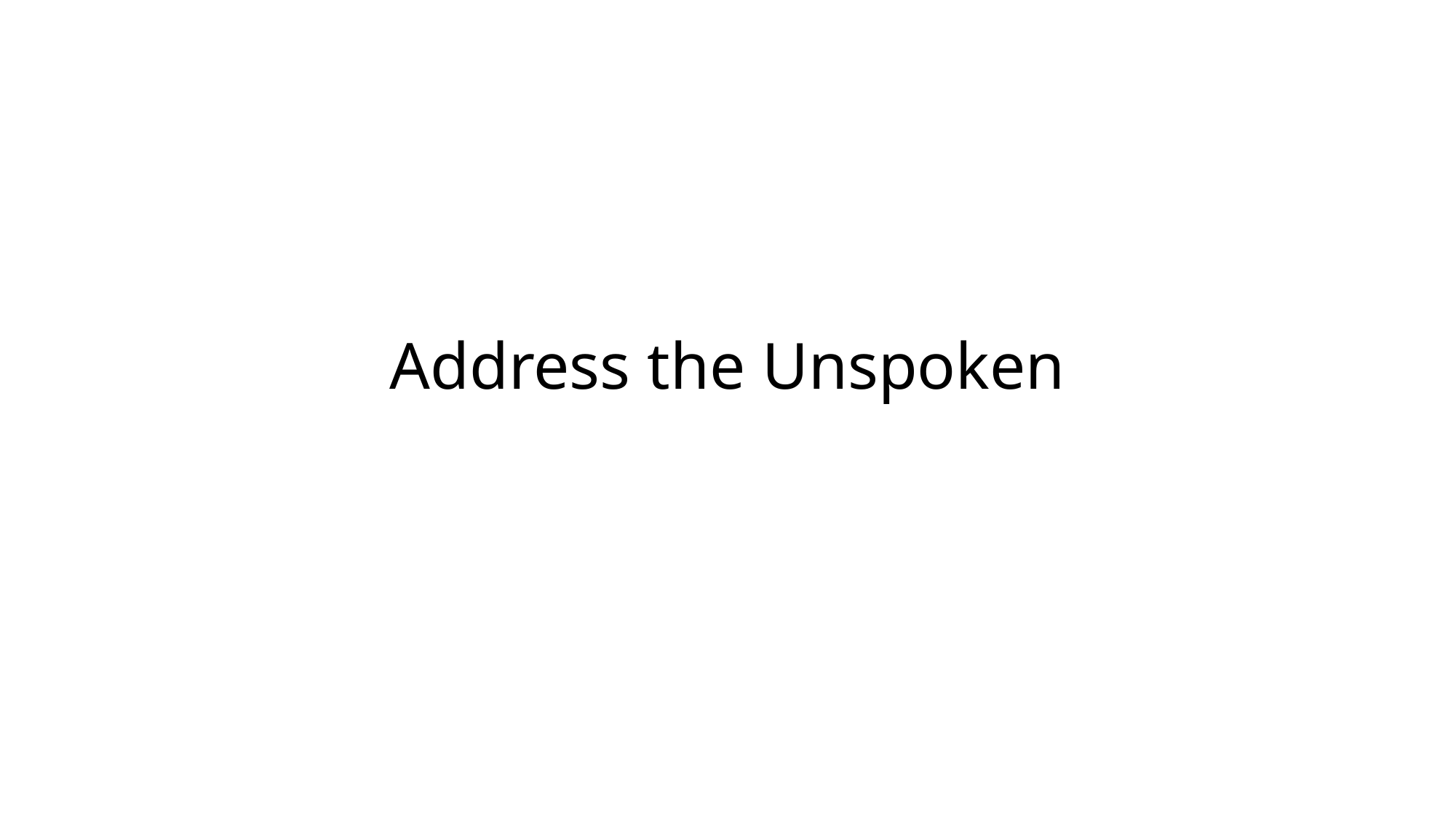

# Address the Unspoken

## Slide 52
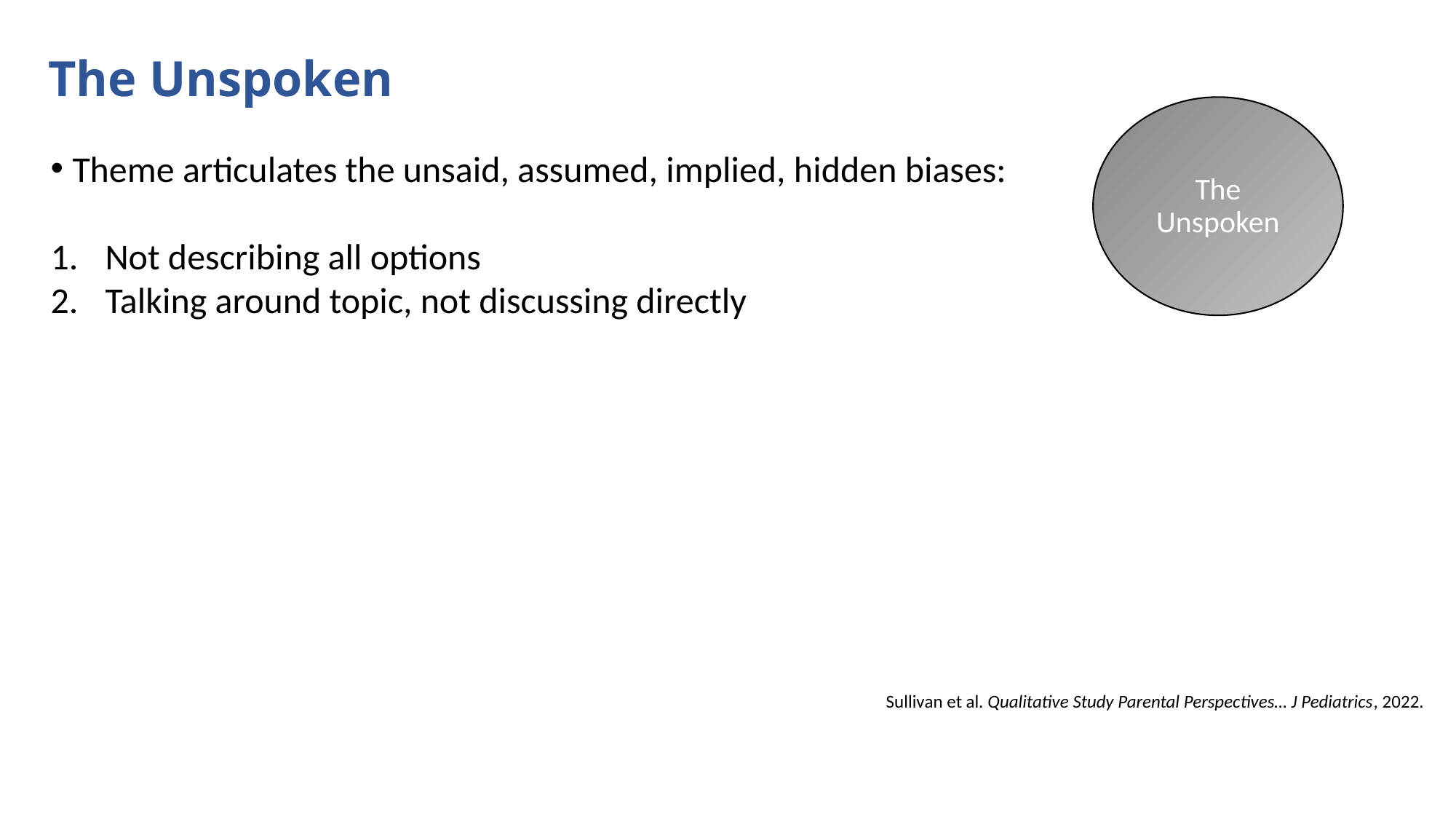

# The Unspoken
The Unspoken
Theme articulates the unsaid, assumed, implied, hidden biases:
Not describing all options
Talking around topic, not discussing directly
Sullivan et al. Qualitative Study Parental Perspectives… J Pediatrics, 2022.

## Slide 53
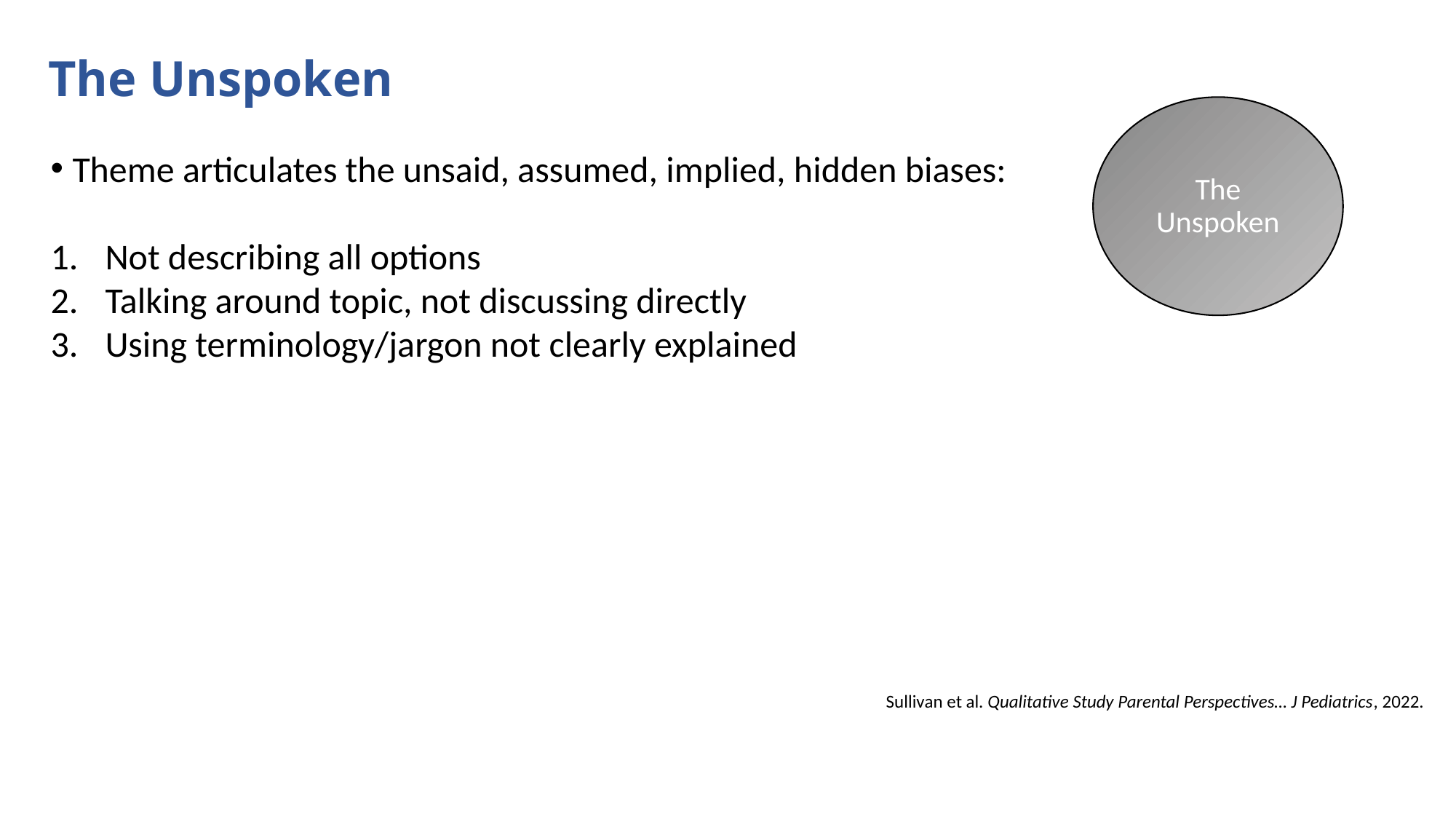

# The Unspoken
The Unspoken
Theme articulates the unsaid, assumed, implied, hidden biases:
Not describing all options
Talking around topic, not discussing directly
Using terminology/jargon not clearly explained
Sullivan et al. Qualitative Study Parental Perspectives… J Pediatrics, 2022.

## Slide 54
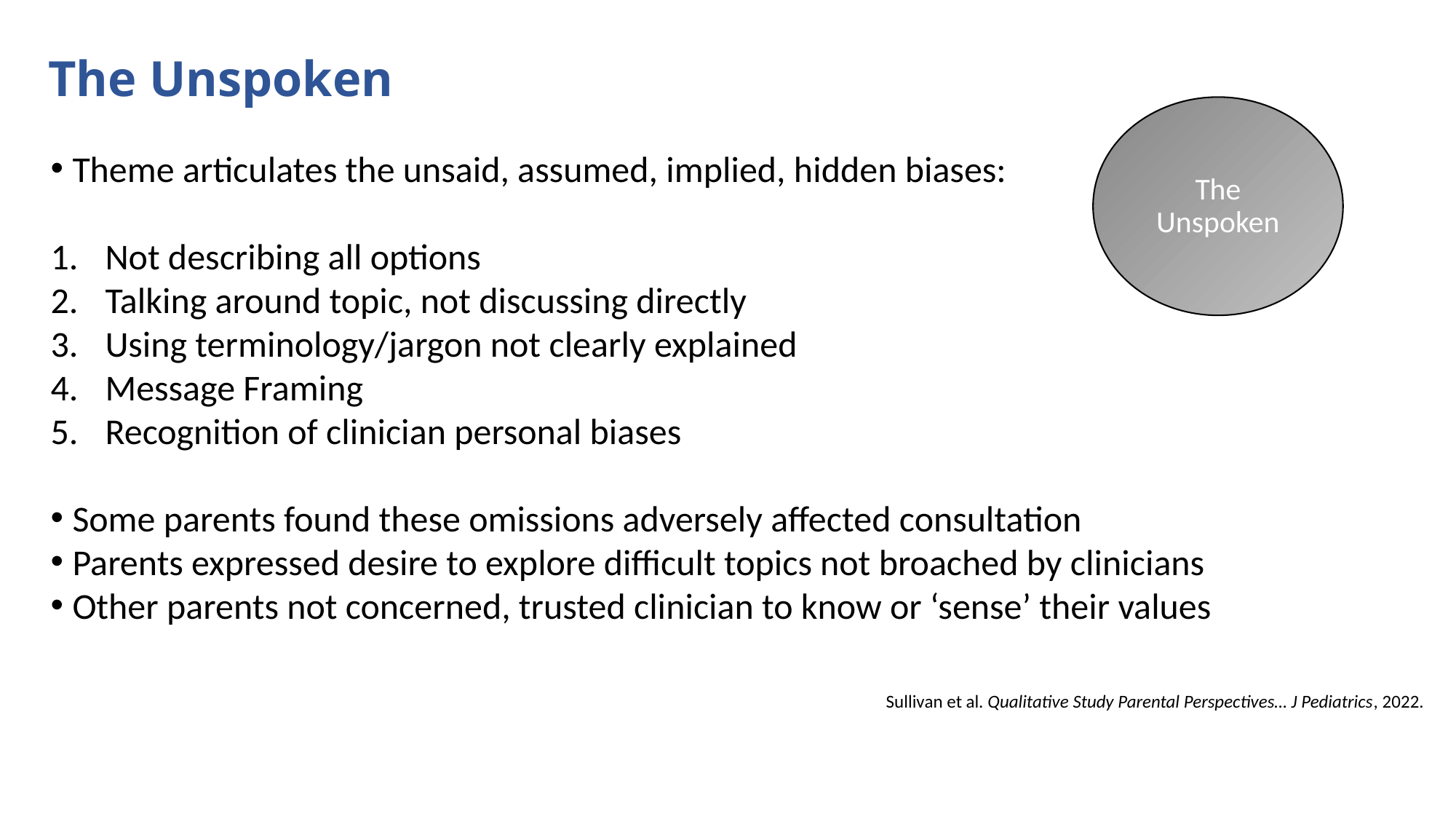

# The Unspoken
The Unspoken
Theme articulates the unsaid, assumed, implied, hidden biases:
Not describing all options
Talking around topic, not discussing directly
Using terminology/jargon not clearly explained
Message Framing
Recognition of clinician personal biases
Some parents found these omissions adversely affected consultation
Parents expressed desire to explore difficult topics not broached by clinicians
Other parents not concerned, trusted clinician to know or ‘sense’ their values
Sullivan et al. Qualitative Study Parental Perspectives… J Pediatrics, 2022.

## Slide 55
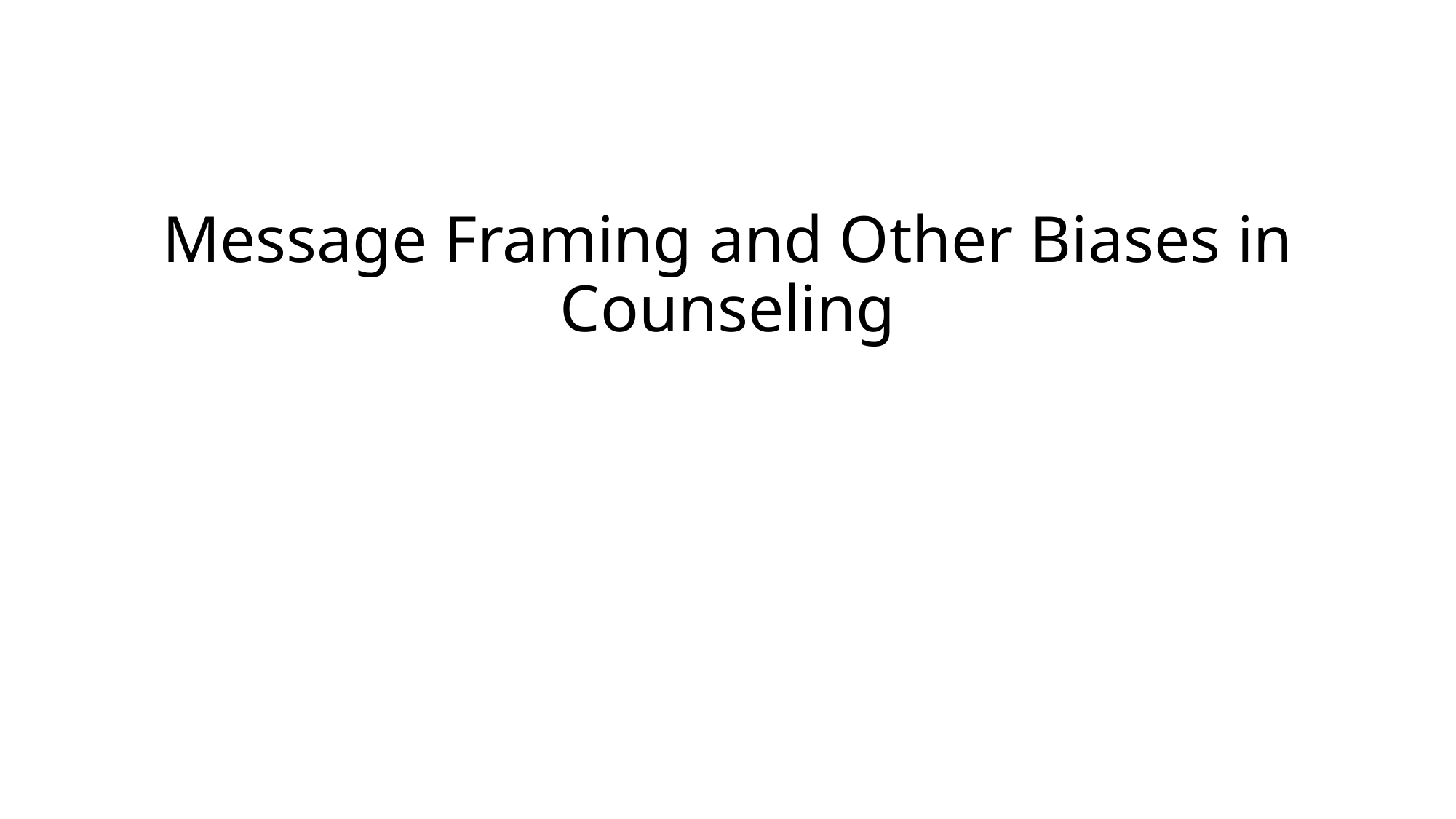

# Message Framing and Other Biases in Counseling

## Slide 56
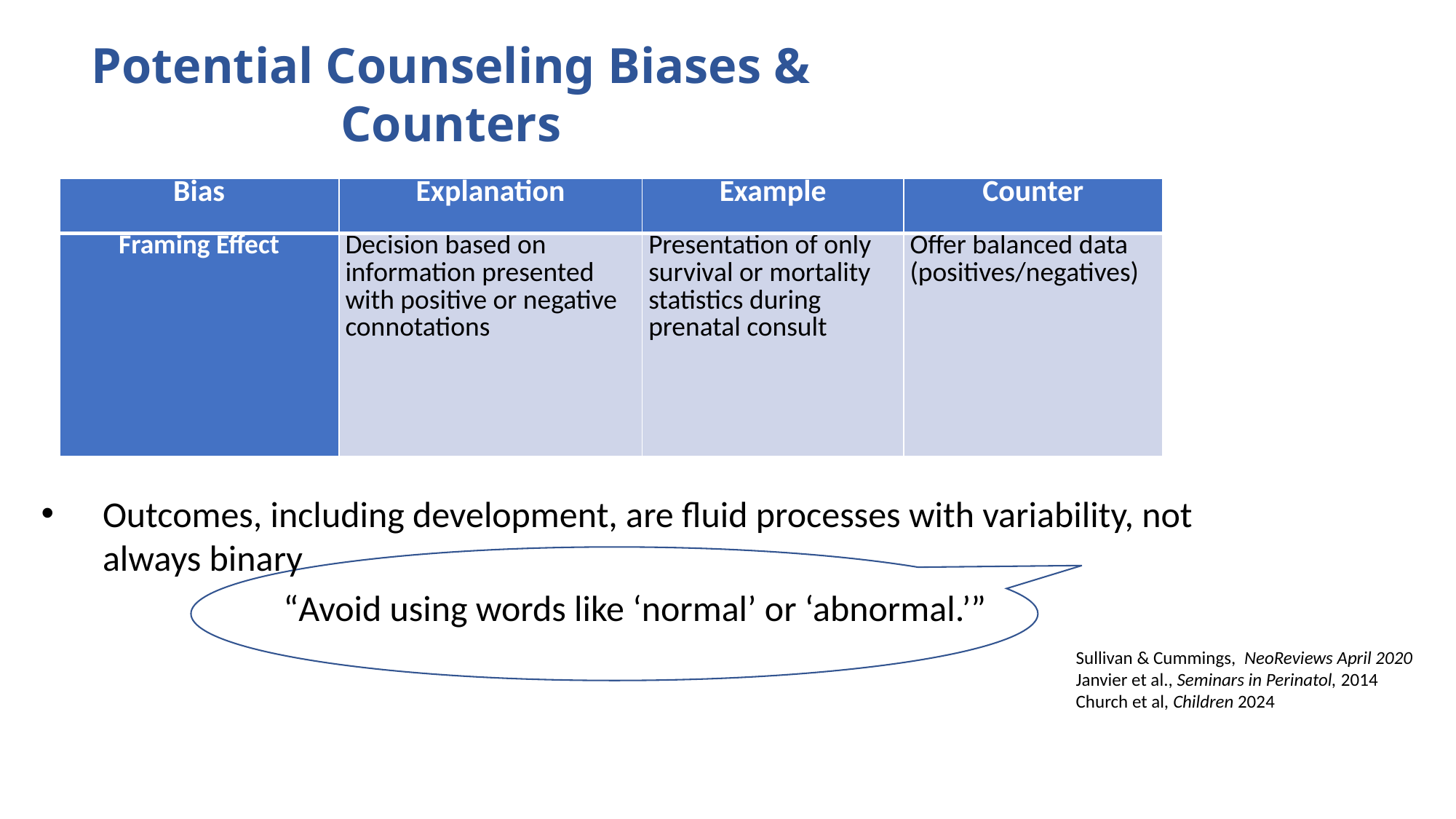

Potential Counseling Biases & Counters
| Bias | Explanation | Example | Counter |
| --- | --- | --- | --- |
| Framing Effect | Decision based on information presented with positive or negative connotations | Presentation of only survival or mortality statistics during prenatal consult | Offer balanced data (positives/negatives) |
Outcomes, including development, are fluid processes with variability, not always binary
“Avoid using words like ‘normal’ or ‘abnormal.’”
Sullivan & Cummings, NeoReviews April 2020
Janvier et al., Seminars in Perinatol, 2014
Church et al, Children 2024

## Slide 57
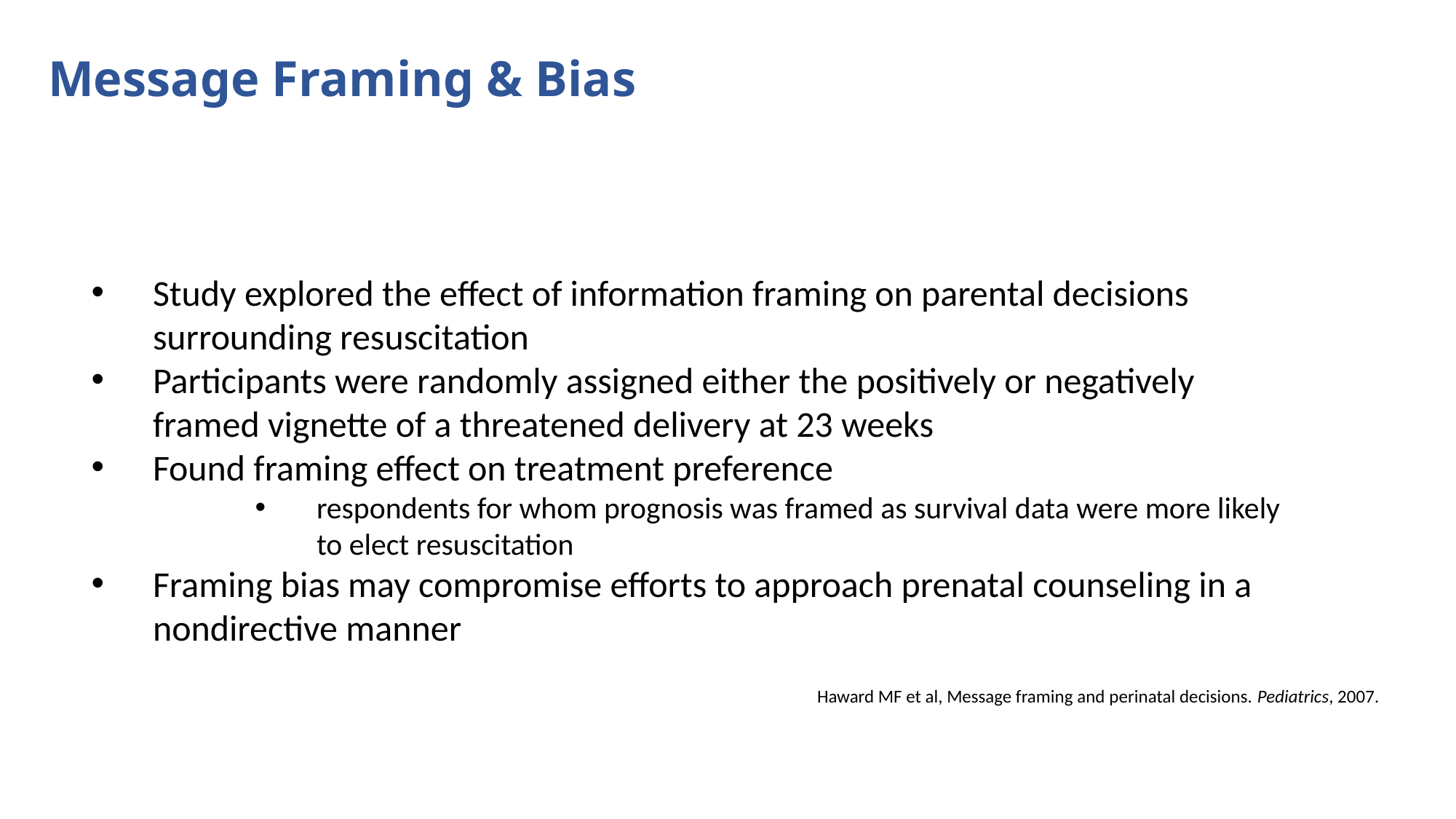

# Message Framing & Bias
Study explored the effect of information framing on parental decisions surrounding resuscitation
Participants were randomly assigned either the positively or negatively framed vignette of a threatened delivery at 23 weeks
Found framing effect on treatment preference
respondents for whom prognosis was framed as survival data were more likely to elect resuscitation
Framing bias may compromise efforts to approach prenatal counseling in a nondirective manner
Haward MF et al, Message framing and perinatal decisions. Pediatrics, 2007.

## Slide 58
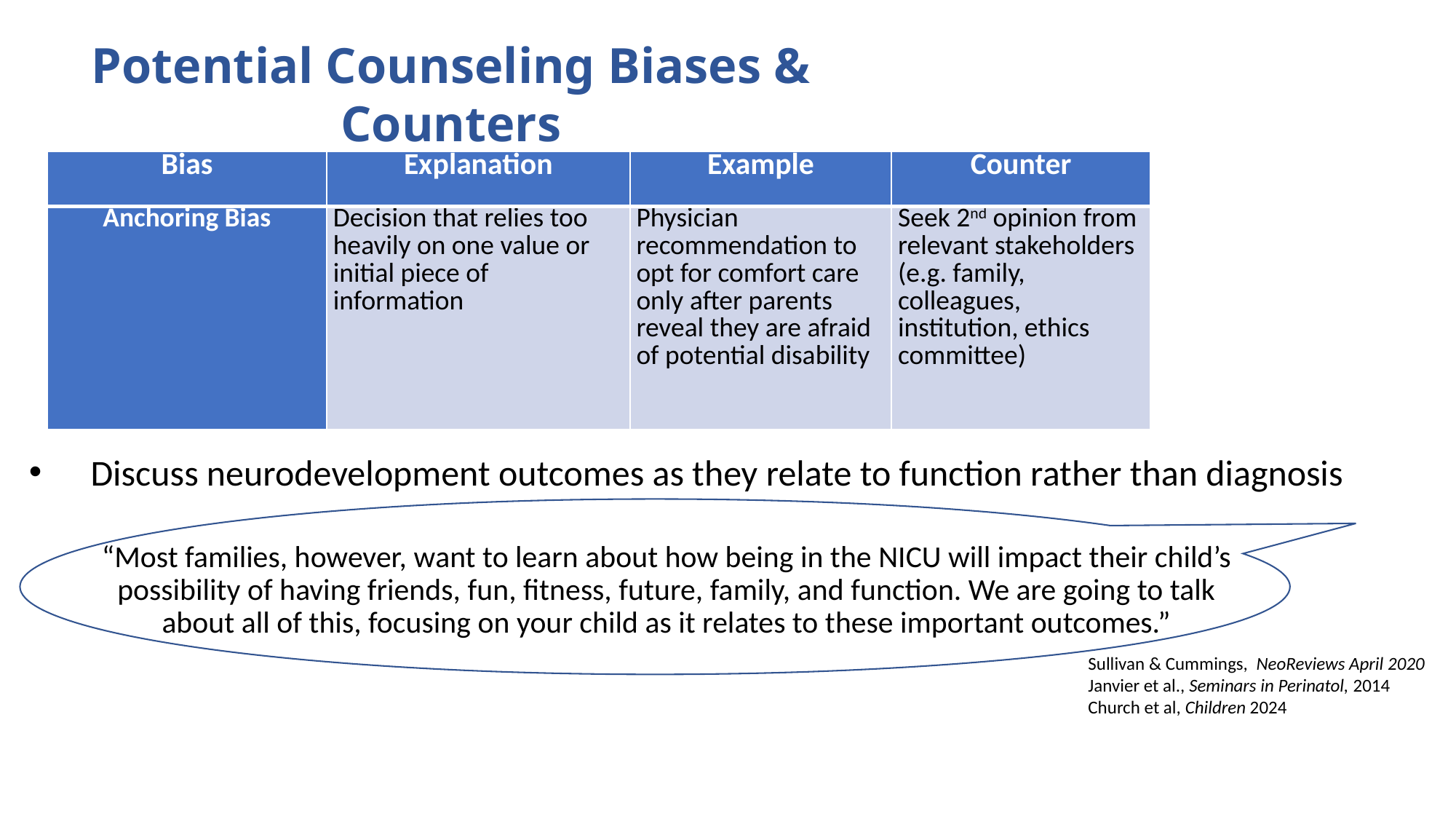

Potential Counseling Biases & Counters
| Bias | Explanation | Example | Counter |
| --- | --- | --- | --- |
| Anchoring Bias | Decision that relies too heavily on one value or initial piece of information | Physician recommendation to opt for comfort care only after parents reveal they are afraid of potential disability | Seek 2nd opinion from relevant stakeholders (e.g. family, colleagues, institution, ethics committee) |
Discuss neurodevelopment outcomes as they relate to function rather than diagnosis
“Most families, however, want to learn about how being in the NICU will impact their child’s possibility of having friends, fun, fitness, future, family, and function. We are going to talk about all of this, focusing on your child as it relates to these important outcomes.”
Sullivan & Cummings, NeoReviews April 2020
Janvier et al., Seminars in Perinatol, 2014
Church et al, Children 2024

## Slide 59
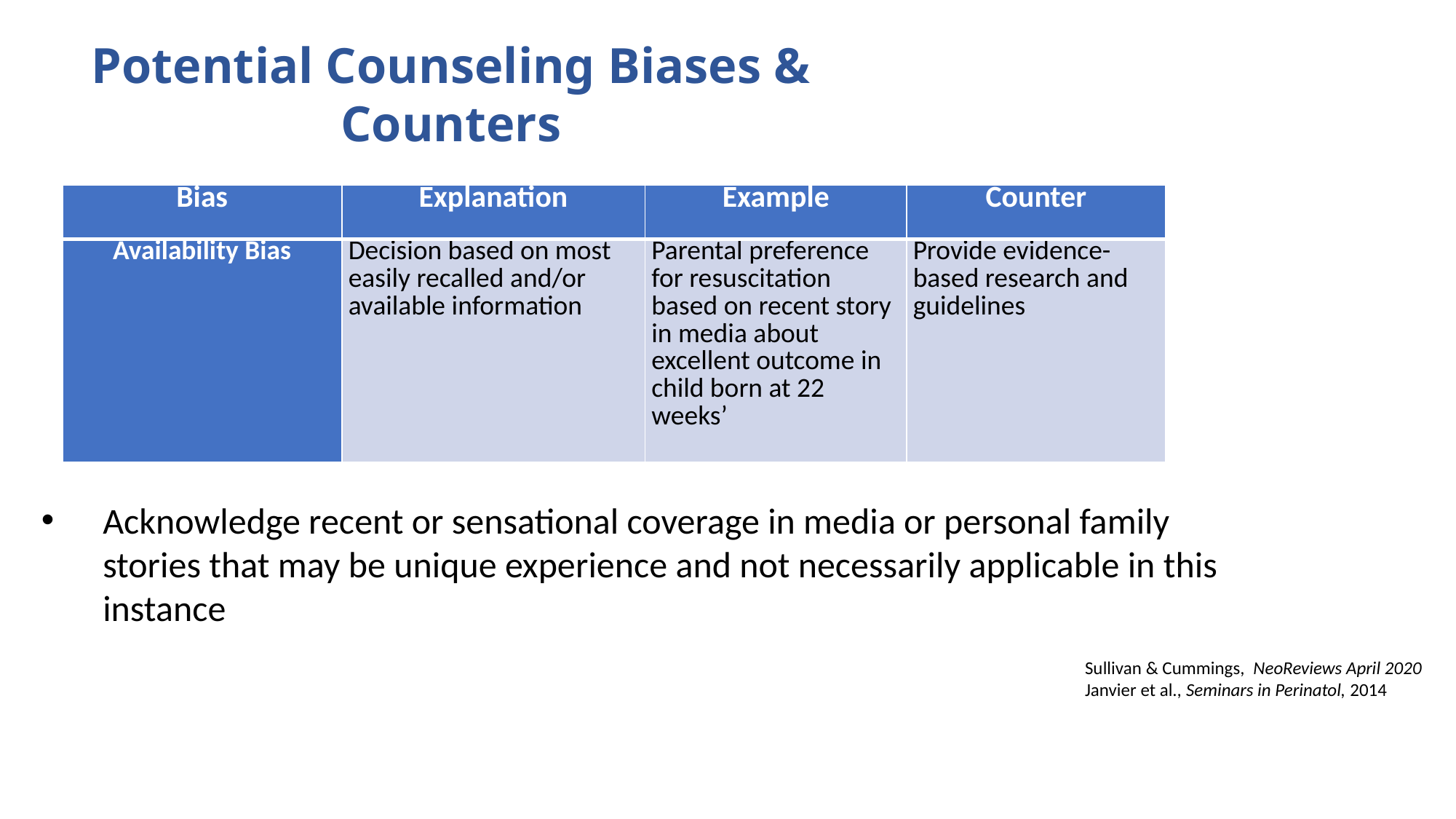

Potential Counseling Biases & Counters
| Bias | Explanation | Example | Counter |
| --- | --- | --- | --- |
| Availability Bias | Decision based on most easily recalled and/or available information | Parental preference for resuscitation based on recent story in media about excellent outcome in child born at 22 weeks’ | Provide evidence-based research and guidelines |
Acknowledge recent or sensational coverage in media or personal family stories that may be unique experience and not necessarily applicable in this instance
Sullivan & Cummings, NeoReviews April 2020
Janvier et al., Seminars in Perinatol, 2014

## Slide 60
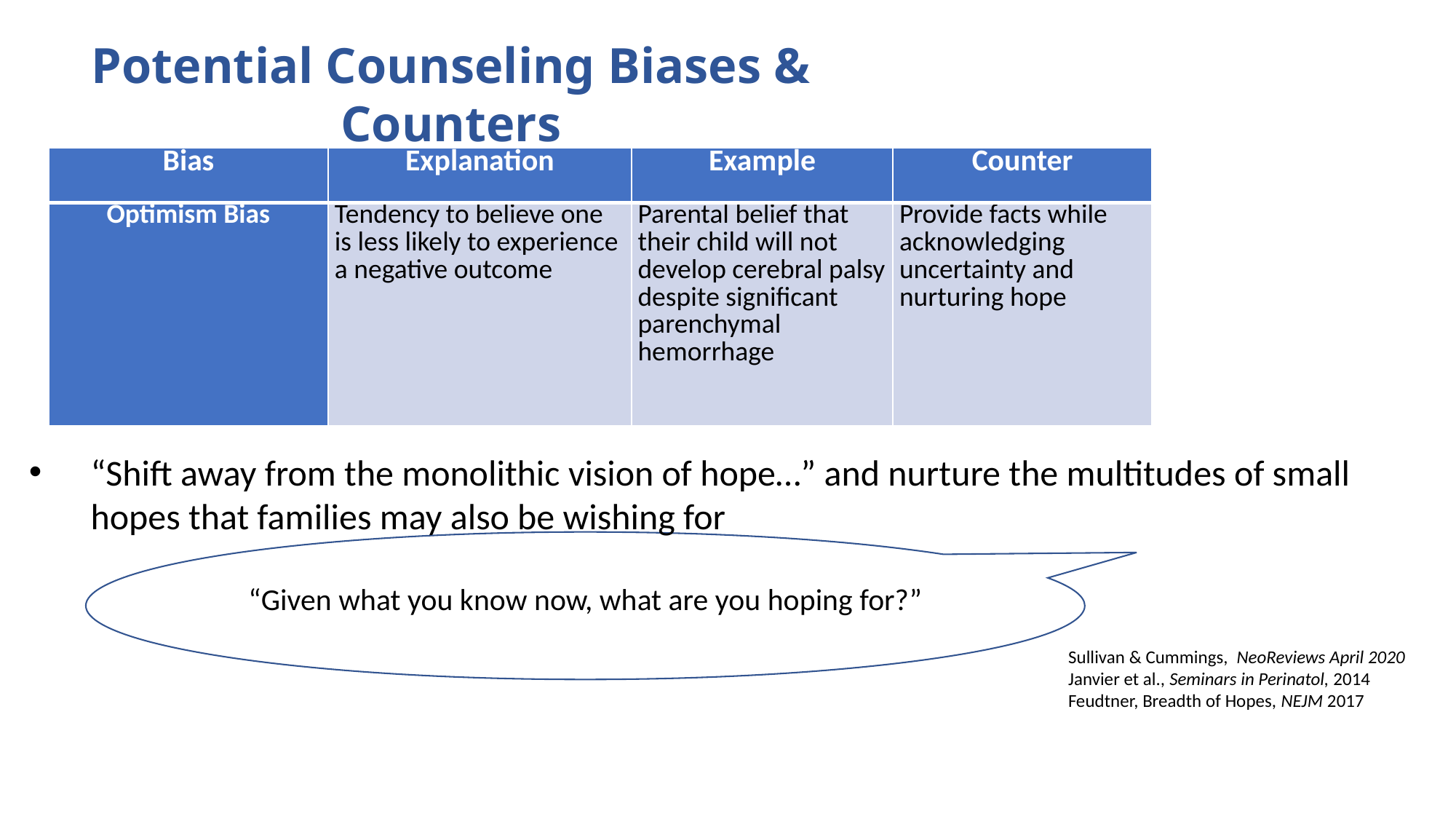

Potential Counseling Biases & Counters
| Bias | Explanation | Example | Counter |
| --- | --- | --- | --- |
| Optimism Bias | Tendency to believe one is less likely to experience a negative outcome | Parental belief that their child will not develop cerebral palsy despite significant parenchymal hemorrhage | Provide facts while acknowledging uncertainty and nurturing hope |
“Shift away from the monolithic vision of hope…” and nurture the multitudes of small hopes that families may also be wishing for
“Given what you know now, what are you hoping for?”
Sullivan & Cummings, NeoReviews April 2020
Janvier et al., Seminars in Perinatol, 2014
Feudtner, Breadth of Hopes, NEJM 2017

## Slide 61
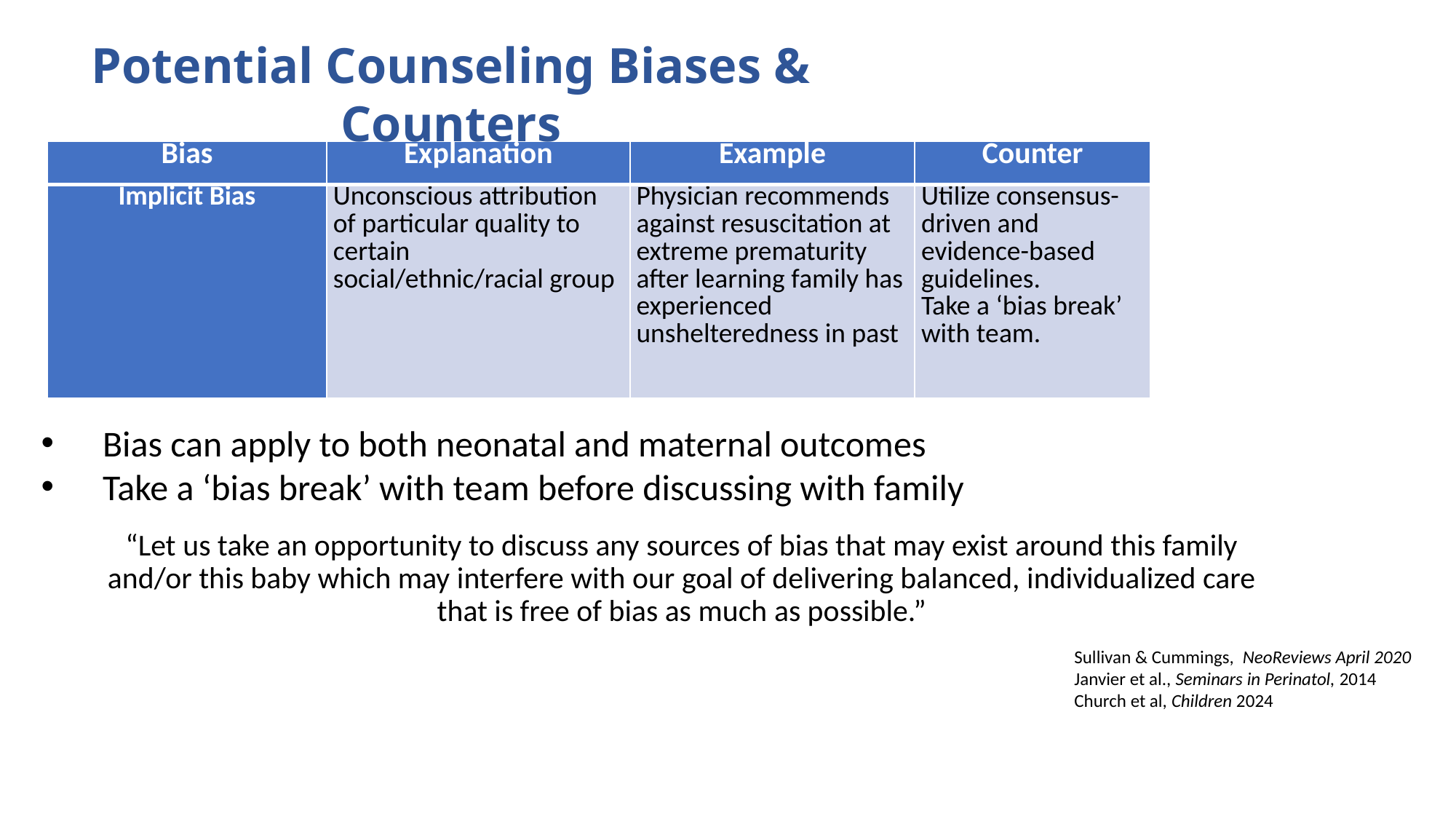

Potential Counseling Biases & Counters
| Bias | Explanation | Example | Counter |
| --- | --- | --- | --- |
| Implicit Bias | Unconscious attribution of particular quality to certain social/ethnic/racial group | Physician recommends against resuscitation at extreme prematurity after learning family has experienced unshelteredness in past | Utilize consensus-driven and evidence-based guidelines. Take a ‘bias break’ with team. |
Bias can apply to both neonatal and maternal outcomes
Take a ‘bias break’ with team before discussing with family
“Let us take an opportunity to discuss any sources of bias that may exist around this family and/or this baby which may interfere with our goal of delivering balanced, individualized care that is free of bias as much as possible.”
Sullivan & Cummings, NeoReviews April 2020
Janvier et al., Seminars in Perinatol, 2014
Church et al, Children 2024

## Slide 62
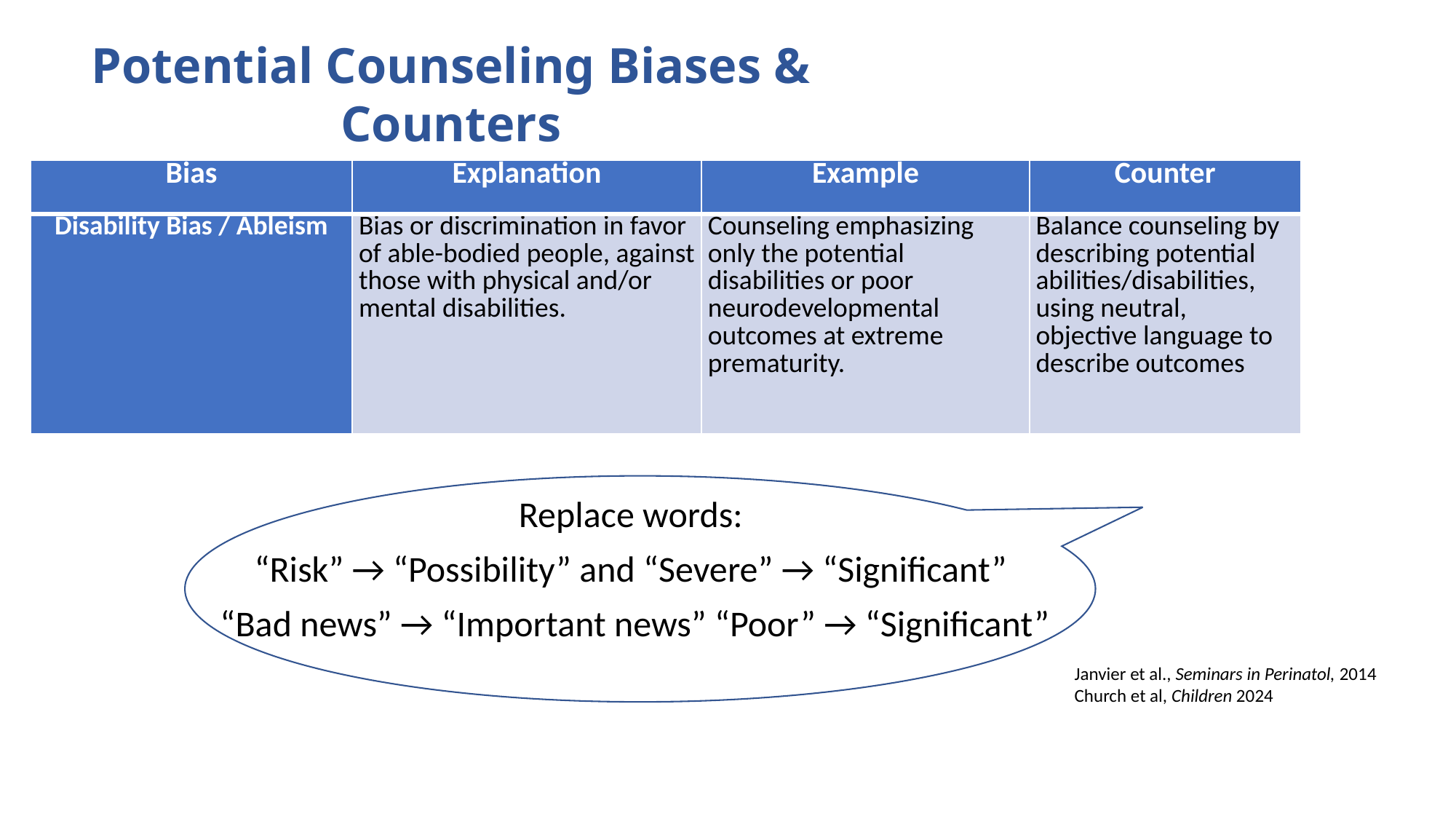

Potential Counseling Biases & Counters
| Bias | Explanation | Example | Counter |
| --- | --- | --- | --- |
| Disability Bias / Ableism | Bias or discrimination in favor of able-bodied people, against those with physical and/or mental disabilities. | Counseling emphasizing only the potential disabilities or poor neurodevelopmental outcomes at extreme prematurity. | Balance counseling by describing potential abilities/disabilities, using neutral, objective language to describe outcomes |
Replace words:
“Risk” → “Possibility” and “Severe” → “Significant”
“Bad news” → “Important news” “Poor” → “Significant”
Janvier et al., Seminars in Perinatol, 2014
Church et al, Children 2024

## Slide 63
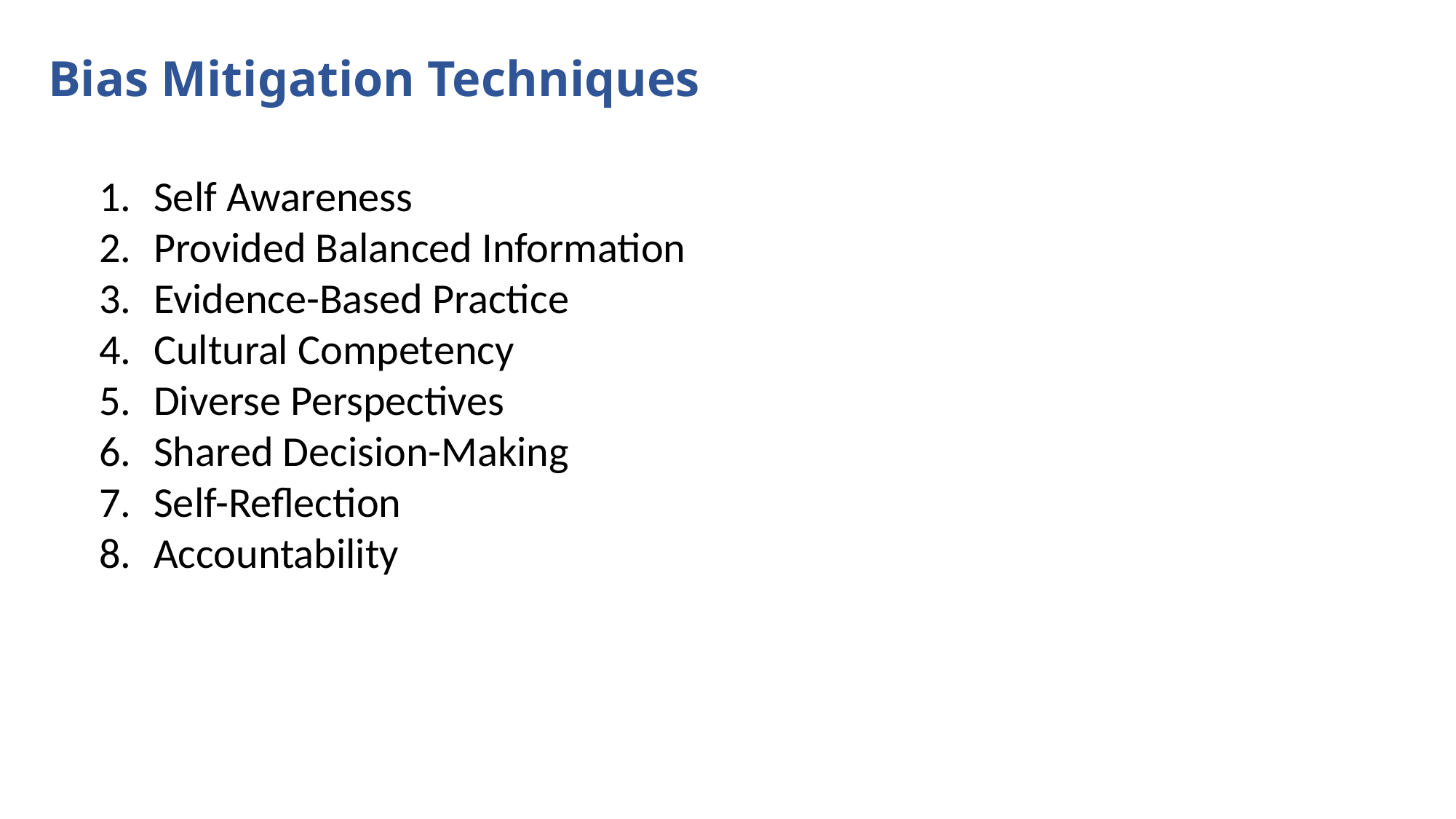

# Bias Mitigation Techniques
Self Awareness
Provided Balanced Information
Evidence-Based Practice
Cultural Competency
Diverse Perspectives
Shared Decision-Making
Self-Reflection
Accountability

## Slide 64
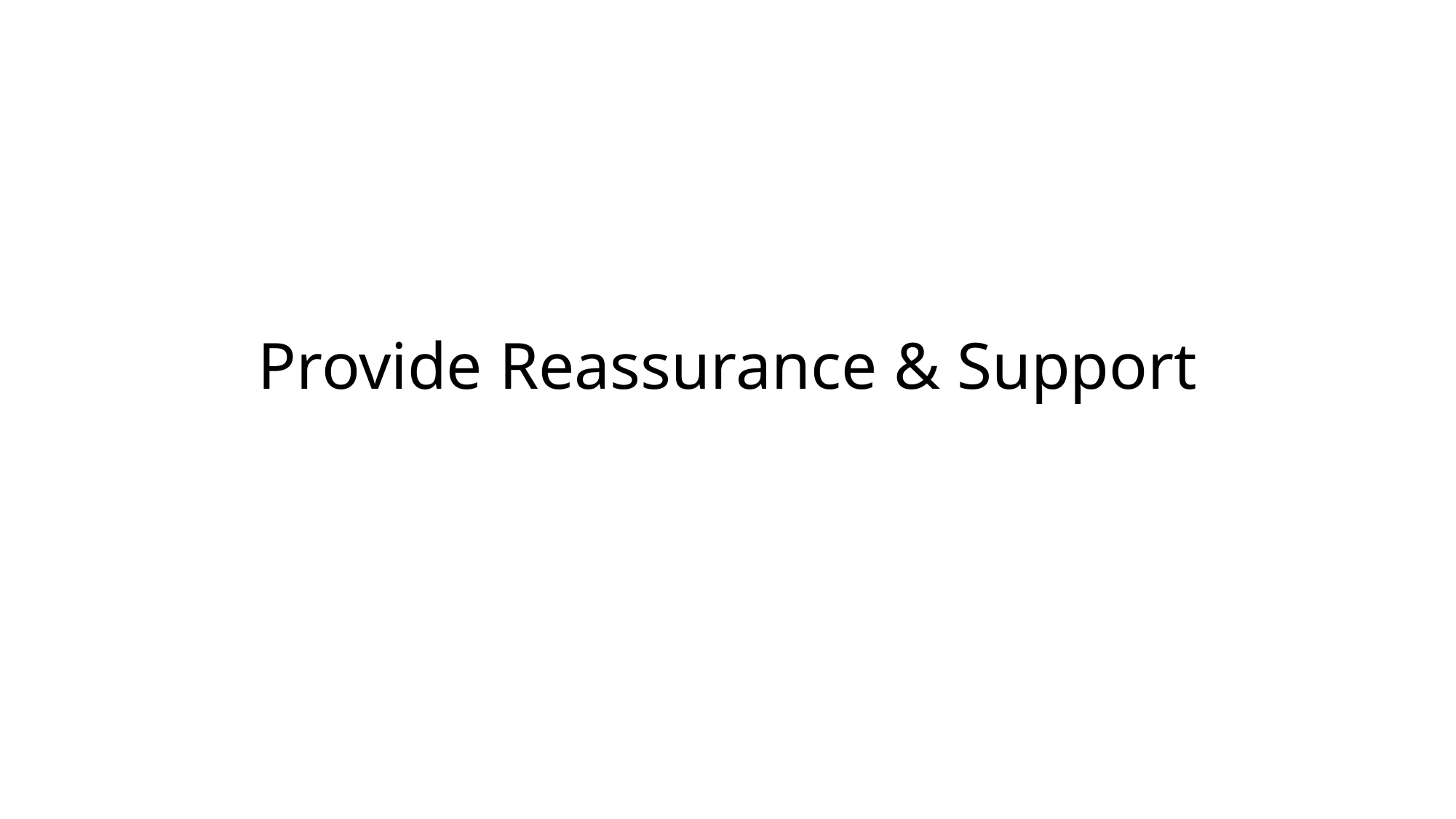

# Provide Reassurance & Support

## Slide 65
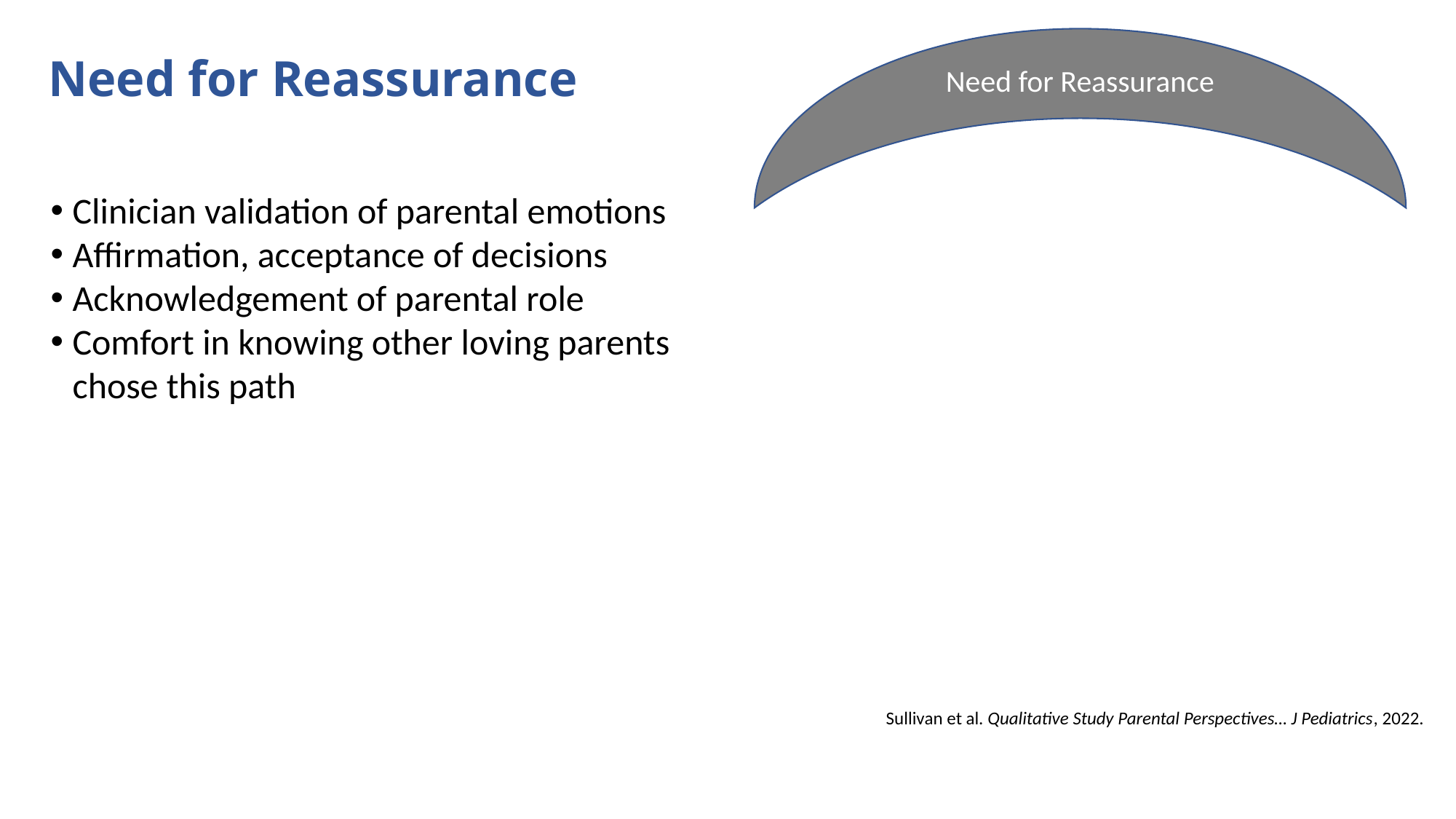

# Need for Reassurance
Need for Reassurance
Clinician validation of parental emotions
Affirmation, acceptance of decisions
Acknowledgement of parental role
Comfort in knowing other loving parents
chose this path
Sullivan et al. Qualitative Study Parental Perspectives… J Pediatrics, 2022.

## Slide 66
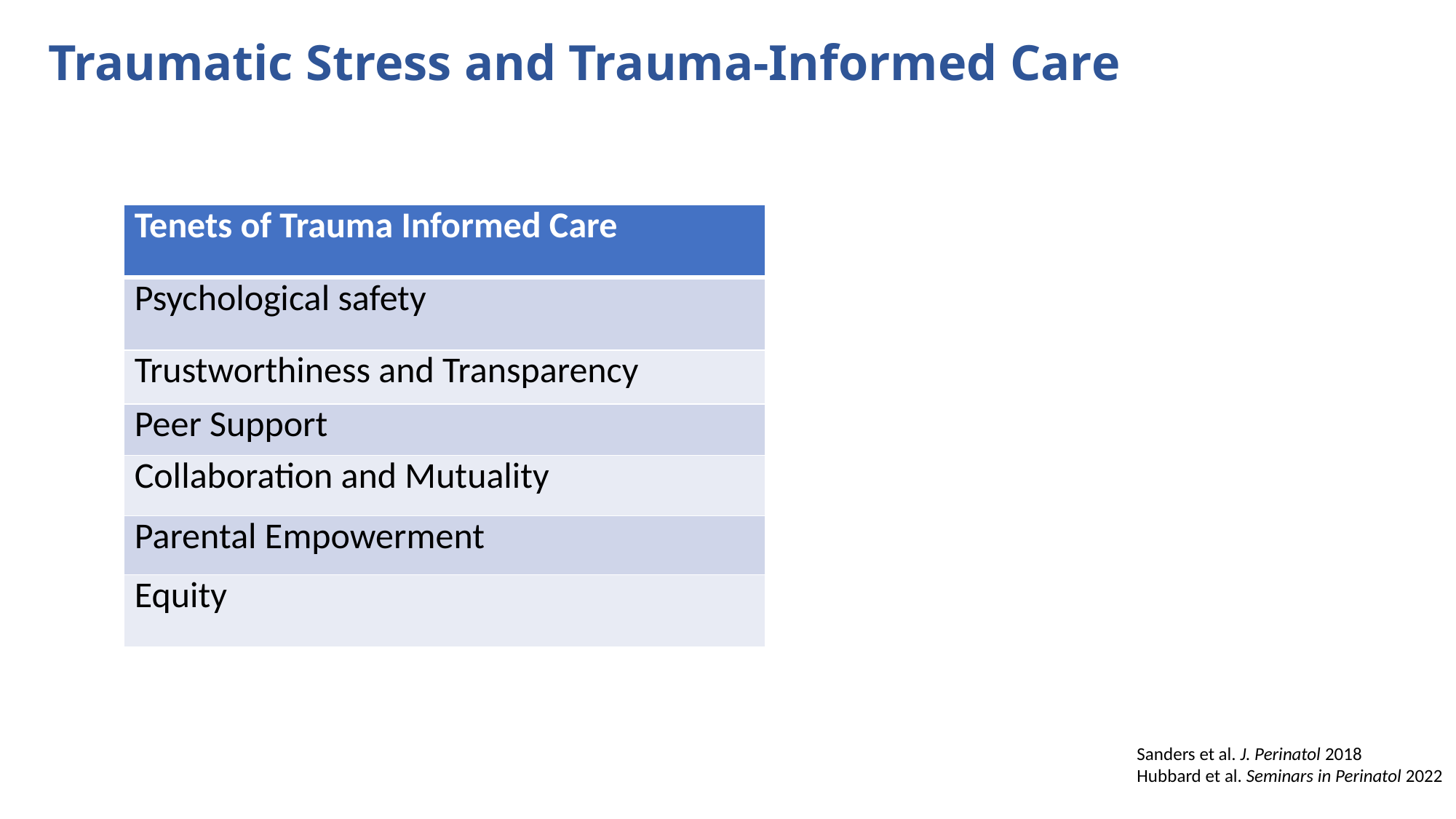

Traumatic Stress and Trauma-Informed Care
| Tenets of Trauma Informed Care |
| --- |
| Psychological safety |
| Trustworthiness and Transparency |
| Peer Support |
| Collaboration and Mutuality |
| Parental Empowerment |
| Equity |
Sanders et al. J. Perinatol 2018
Hubbard et al. Seminars in Perinatol 2022

## Slide 67
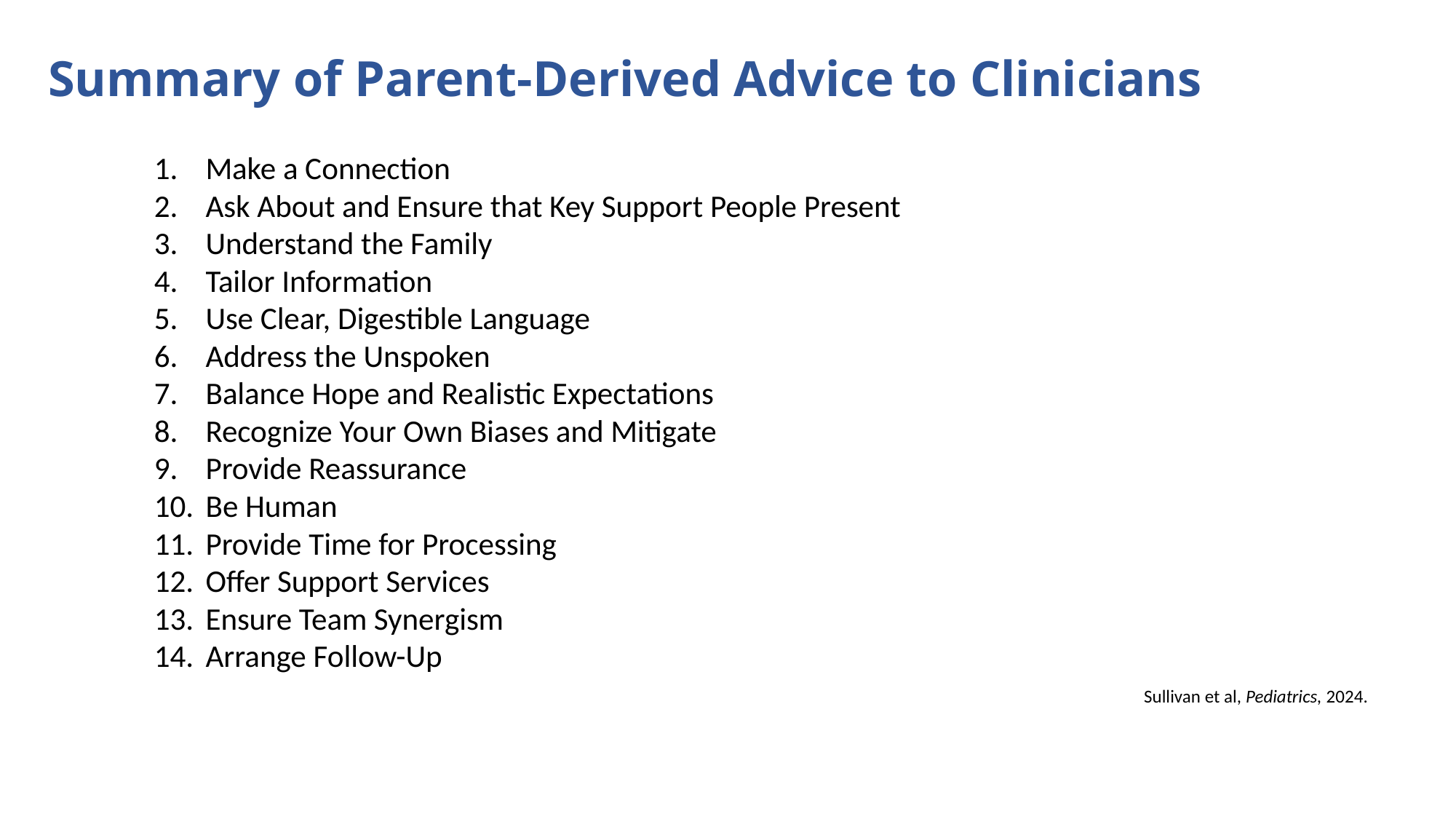

# Summary of Parent-Derived Advice to Clinicians
Make a Connection
Ask About and Ensure that Key Support People Present
Understand the Family
Tailor Information
Use Clear, Digestible Language
Address the Unspoken
Balance Hope and Realistic Expectations
Recognize Your Own Biases and Mitigate
Provide Reassurance
Be Human
Provide Time for Processing
Offer Support Services
Ensure Team Synergism
Arrange Follow-Up
Sullivan et al, Pediatrics, 2024.

## Slide 68
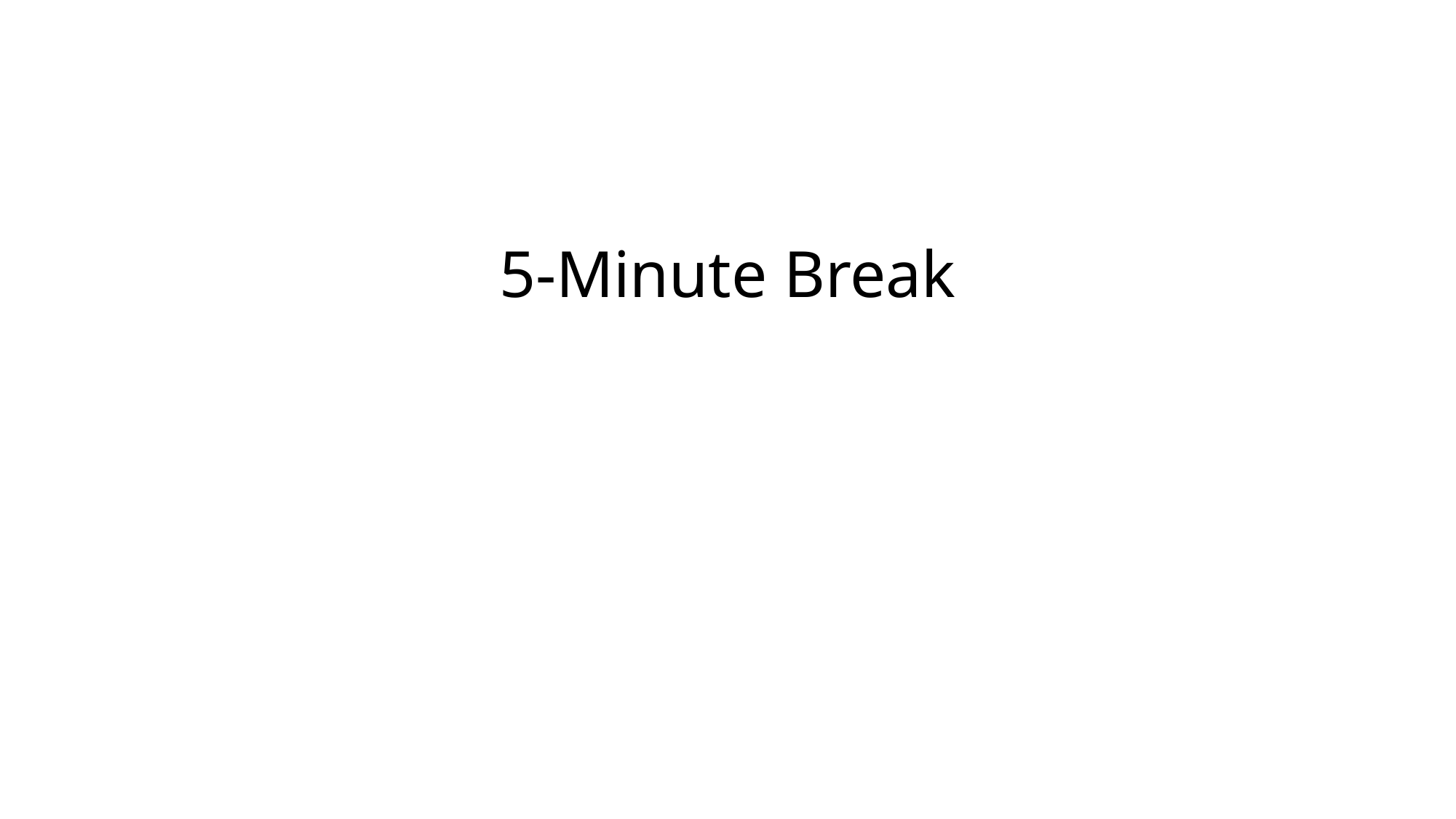

# 5-Minute Break

## Slide 69
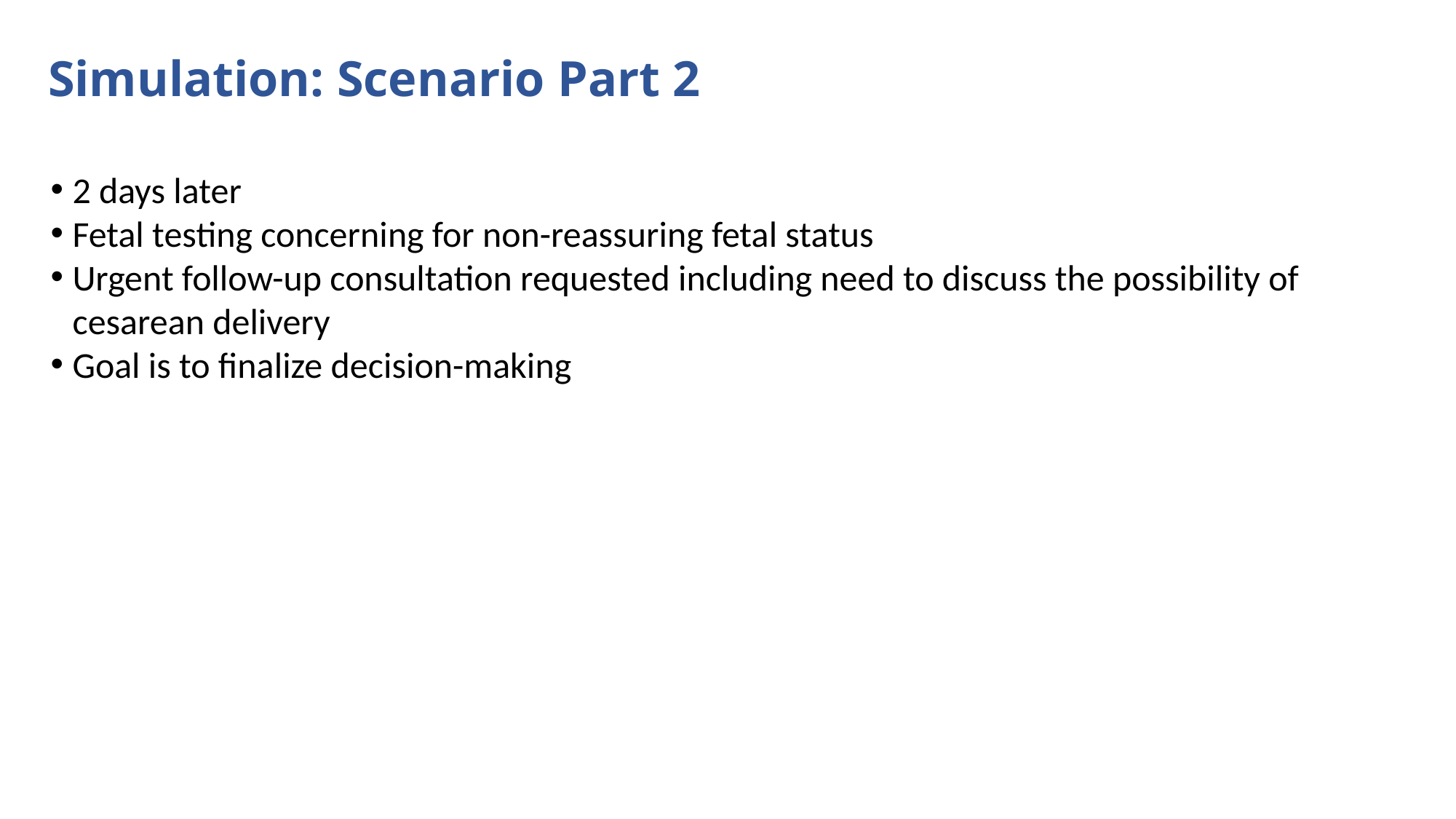

# Simulation: Scenario Part 2
2 days later
Fetal testing concerning for non-reassuring fetal status
Urgent follow-up consultation requested including need to discuss the possibility of cesarean delivery
Goal is to finalize decision-making

## Slide 70
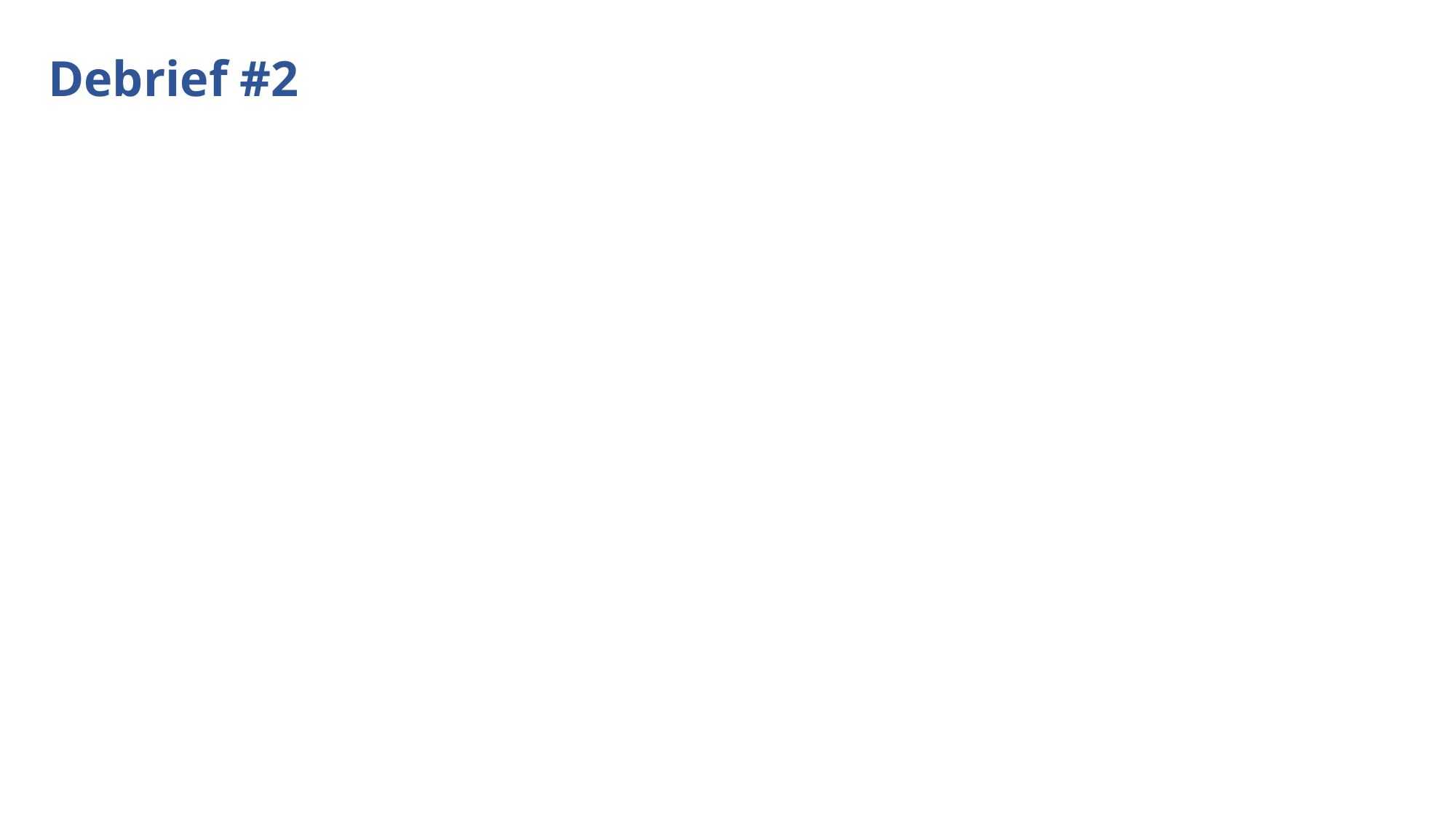

# Debrief #2

## Slide 71
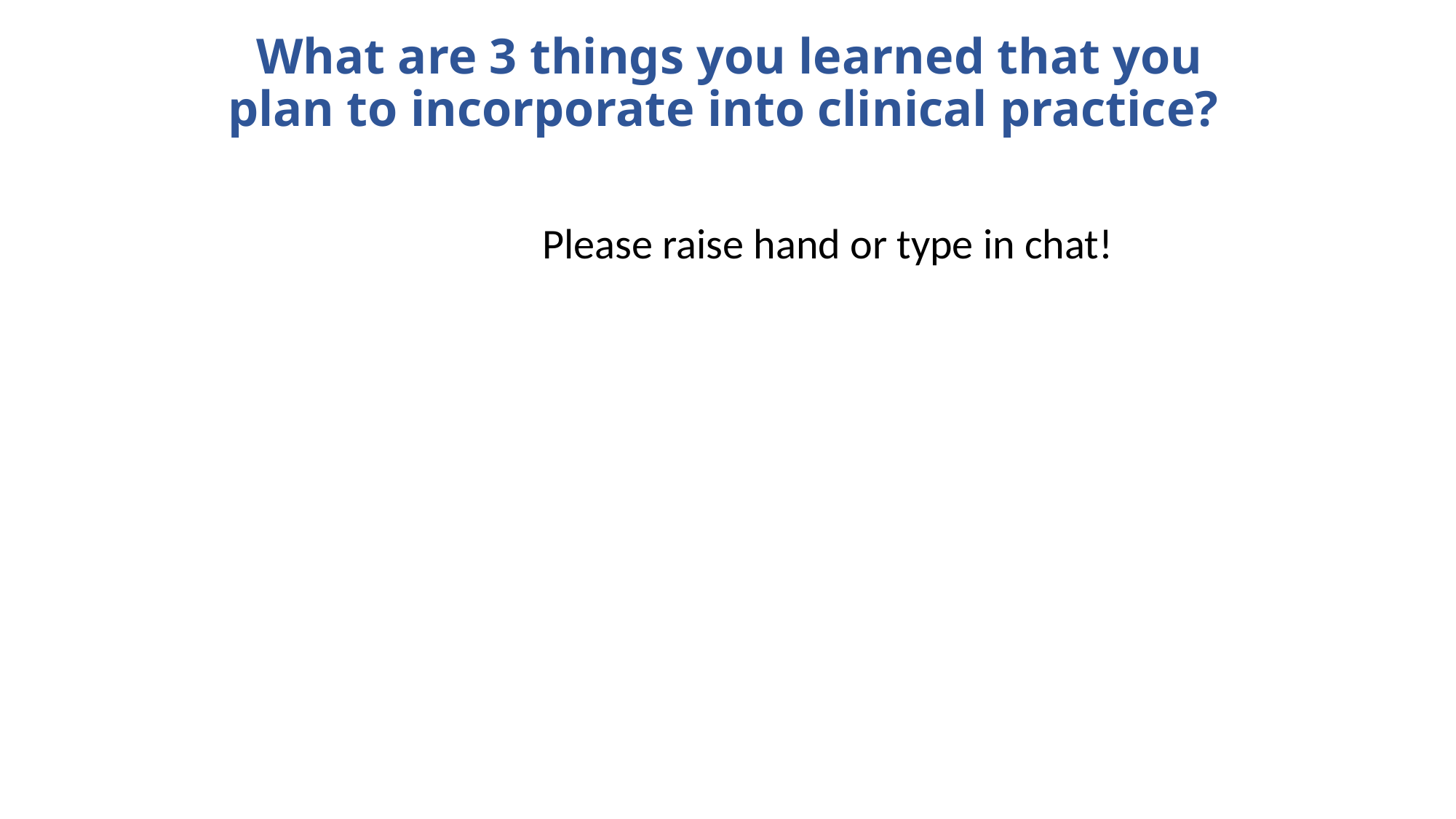

# What are 3 things you learned that you plan to incorporate into clinical practice?
Please raise hand or type in chat!
