# Supplementary material for: Development of a Joint-Specialty Simulation-Based Workshop to Optimize Counseling at Extreme Prematurity
Source: MedEdPORTAL. 2026 Jul 29;22:11623. doi: 10.15766/mep_2374-8265.11623 (PMC13415433; doi:10.15766/mep_2374-8265.11623)
Supplement: Supplementary file 1 — Prenatal Counseling Workshop.pptxPrenatal Counseling Case.docxFacilitator Guide.docxPostworkshop Survey.docx [file mep_2374-8265.11623-s001.zip › B. Prenatal Counseling Case.docx]

**Appendix B: Prenatal Counseling Case**

Date: May 2024

Primary Case Author: Anne Sullivan

Secondary Case Author: Christy Cummings

Simulated Participant Educator: Anne Sullivan and Christy Cummings

Name of Case: Optimizing Counseling at Extreme Prematurity

Name of Educational and/or Assessment Activity: Joint-Specialty Workshop on Optimizing Counseling at Extreme Prematurity

Patient Name: Gabrielle Davis

Chief Complaint: Premature rupture of membranes at 23 weeks’ gestation

Most Likely Diagnosis and Differential With Rationale From History and/or Physical Exam:

Preterm labor at extreme prematurity with need for shared decision-making surrounding preferences for resuscitation of their baby if born prematurely.

Challenge Question: N/A

Domains: Check all that apply

- **Professionalism**
- **Communication and Interpersonal Skills**
- Medical History
- Physical Exam
- **Shared Decision-Making**
- Patient Education
- Clinical Reasoning
- Documentation
- Handoff
- Presentation
- Other:

Type and Level of Learner: Neonatology and Maternal Fetal Medicine Clinicians and Trainees

Case Objectives: Please list specific objectives for each of the domains you have checked above:

1. Recognize the importance of effective interspecialty communication and collaboration during prenatal counseling by applying newly acquired communication techniques and relational skills.
2. Apply bias mitigation techniques to counseling expectant parents at extreme prematurity
3. Demonstrate communication strategies that reflect and incorporates the values, preferences, and perspective of families during counseling expectant parents at extreme prematurity
4. Incorporate evidence-based advice from parents when counseling expectant paretns at extreme prematurity

**Patient- Gabrielle Davis**

| SETTING: outpatient, in patient, ED, home, nursing home, rehab, group, etc. | An expectant mother at 23 weeks’ gestation is admitted to an inpatient room on labor and delivery floor after her water breaks at home. |
| --- | --- |
| PATIENT PROFILE: Information about the “patient” that helps select an SP and helps the learner get an understanding of them as a person. SP will know more information about the patient than learner will ever ask but allows SP to portray a fully developed patient personality. If none of the items below are particulars for the case, please write “all may be used.” | |
| Age range | 25-40 |
| Religious/spiritual background | Strong belief in God. Faith important, believes in miracles |
| Sex (e.g., male, female, intersex, transwoman, transman) | Female |
| Sexual orientation (e.g., heterosexual, lesbian, gay, bisexual, pansexual, queer, asexual) | All may be used |
| Gender expression (e.g., man, woman, genderqueer) | Woman |
| Race and ethnicity (e.g., to promote educational diversity, we use a diverse pool of SPs.) | All may be used, but to enhance education surrounding bias mitigation, a diverse pool of SPs is preferred. |
| Physical description (e.g., BMI, height range) | All may be used |
| Physical limitations | All may be used |
| Patient appearance (e.g., disheveled, hospital gown, business casual, casual) | Worried pregnant patient (at 23 weeks, so small pregnancy belly) in hospital gown |
| Moulage + location (e.g., none, bruises, scars, body piercing, tattoos) | All may be used |
| Affect (e.g., pleasant, cooperative) | Anxious, in shock |
| Family group (e.g., who is family, who they live with) | Lives with husband, Cameron, along with daughter Isabelle (from husband’s prior relationship) |
| Education | College Graduate |
| Level of health literacy | Low to moderate. No formal medical background. Understands most concepts when stated clearly and with sufficient explanation. |
| Employment, if any - present and past, noting any current stresses | Special education teacher |
| Home/homeless - type of dwelling, number of stories, owned or rented | Any |
| Financial situation - any current stresses | Partner, Cameron is unemployed, which has caused some financial stress |
| Insurance status (e.g., un/under/insured, public/private, HMO/PPO) | Insured through her work |
| Habits (i.e., diet, exercise, caffeine, smoking, alcohol, drugs) | No history of alcohol, tobacco, or drug abuse. |
| Activities (i.e., hobbies, sports, clubs, friends) | Any |
| Typical day - what is the usual daily routine | Any |

**Partner- Cameron Davis**

| SETTING: outpatient, in patient, ED, home, nursing home, rehab, group, etc. | Either inpatient on L&D with wife, Gabrielle or calling in via phone due to childcare constraints. |
| --- | --- |
| PATIENT PROFILE: Information about the “patient” that helps select an SP and helps the learner get an understanding of them as a person. SP will know more information about the patient than learner will ever ask but allows SP to portray a fully developed patient personality. If none of the items below are particulars for the case, please write “all may be used.” | |
| Age range | 25-40 |
| Religious/spiritual background | Strong belief in God. Faith important, believes in miracles |
| Sex (e.g., male, female, intersex, transwoman, transman) | Male |
| Sexual orientation (e.g., heterosexual, lesbian, gay, bisexual, pansexual, queer, asexual) | All may be used |
| Gender expression (e.g., man, woman, genderqueer) | Man |
| Race and ethnicity (e.g., to promote educational diversity, we use a diverse pool of SPs.) | All may be used, but to enhance education surrounding bias mitigation, a diverse pool of SPs is preferred. |
| Physical description (e.g., BMI, height range) | All may be used |
| Physical limitations | All may be used |
| Patient appearance (e.g., disheveled, hospital gown, business casual, casual) | Any |
| Moulage + location (e.g., none, bruises, scars, body piercing, tattoos) | All may be used |
| Affect (e.g., pleasant, cooperative) | Quiet, supportive, anxious |
| Family group (e.g., who is family, who they live with) | Lives with wife, Gabrielle, along with daughter Isabelle (from prior relationship) |
| Education | Completed high school and 2-year community college |
| Level of health literacy | Low to moderate. No formal medical background. Understands most concepts when stated clearly and with sufficient explanation. |
| Employment, if any - present and past, noting any current stresses | Currently unemployed, was a manager at Target, but lost his job during the COVID-19 pandemic and hasn’t been able to secure position since. Source of tension at home. |
| Home/homeless - type of dwelling, number of stories, owned or rented | Any |
| Financial situation - any current stresses | Financial stress with unemployment |
| Insurance status (e.g., un/under/insured, public/private, HMO/PPO) | None at the moment |
| Habits (i.e., diet, exercise, caffeine, smoking, alcohol, drugs) | No history of alcohol, tobacco, or drug abuse. |
| Activities (i.e., hobbies, sports, clubs, friends) | Any |
| Typical day - what is the usual daily routine | Any |

| CASE INFORMATION | |
| --- | --- |
| Chief Concern: What the patient will say when greeted by the student. The patient’s primary reason for seeking medical care often stated in their own words. | “My water broke at home” |
| Additional Concerns: Other, if any, concerns the patient has today (i.e., symptoms, requests, expectations, etc.) that will become part of set agenda. |  |
| THE PATIENT’S STORY: The SP will be asked to tell their symptom story and the personal and emotion impact for each of their concerns. You will want to write this in the patient’s voice. The symptom story should be able to answer this question: “Tell me more about [chief concern/additional concern], starting at the beginning and bringing me up to now.”  The personal context should be able to answer questions concerning the broader personal/psychosocial context of symptoms, especially the patient’s beliefs/attributions.  The emotional context should be able to ask how are you doing with this, how does this make you feel, how has this affected you emotionally? IMPACT: How has this affected your life? How has this been for your family? | I’ve struggled with infertility for years because of my endometriosis, which has meant endless doctor’s appointments. This pregnancy was so deeply hoped for—it took IVF to get here. Before this, I went through multiple miscarriages. I still carry a lot of guilt that I couldn’t get pregnant naturally, and losing those pregnancies was devastating. It even created tension between Cameron and me.  Now, with this pregnancy going wrong too, I can’t help but wonder what I’ve done to cause it. My faith is a big part of my life, and I believe strongly in God. I’m praying for a miracle. We already know we’re having a boy—we’re naming him Damien, after my dad.  **Trigger Comments/Questions** for SP to use during Simulation:   1. If jargon used, act confused or stop paying attention 2. If given a lot of statistical information, act overwhelmed 3. Offer name/gender of baby only if asked 4. If given information on resuscitation options, ask “Is there a choice here?” 5. If offered a choice, ask “What would you do?” 6. If asked what goals are, state “to stay pregnant” 7. Reference difficulty conceiving when discussing values and how this baby is a gift 8. If given bad news or a poor prognosis, reference your hope for your baby and faith, only provide more about faith if directly asked. If probed, do state that you would not want your son to suffer needlessly, or to be able to interact with the world, feel love 9. If there appears to be differences between MFM and NICU, ask ‘if the teams are on the same page?” 10. If provider states baby may die/not survive, become tearful and stop paying attention 11. Here are some other potential comments to bring in:     1. “I don’t know who to trust around here”     2. “Is my baby going to be okay?”     3. “What are the chances my baby will survive if he’s born now?”     4. “How sure are you? Is there a chance everything might turn out ok?”     5. “If I need surgery or medication, will that make it harder for me to have another baby?”     6. “I keep wondering if this is somehow my fault.”   **Partner**  I’m so excited to be having a child with Gabrielle. We’ve wanted this for so long. But, if I’m honest, I’m also worried—especially about what it would mean if our baby were to have severe disabilities. I don’t share these fears much with Gabrielle because I want to be supportive, and I know how much this pregnancy means to her.  Part of me can’t help thinking about the financial side. With just Gabrielle’s salary, raising a child with complex medical needs could be overwhelming. I’ve seen it firsthand—my cousin has trisomy 21. He had a few heart surgeries when he was young, still gets sick a lot, and sometimes needs to be in the hospital. Now, as an adult, he needs full-time care. I love him, but I’ve also seen how hard it can be on a family.  I’m also incredibly worried about Gabrielle and her health, and what the possibility of preterm labor means for her.  **Trigger Comments/Questions** for SP to use during Simulation:   1. Offer name/gender of baby if asked 2. If discussion of a prolonged NICU stay and post-NICU medical needs, reply with worry about “how we will manage this with our finances?” 3. If impairment or delay mentioned, ask what that means and ask if their son will be like his cousin who has trisomy 21 (can use the word “Downs”). 4. If asked about values, ask what they mean by that. If further expanded, say you want your son to “be normal” 5. If option of ‘comfort care’ offered, ask if that means their son will die, “like pull the plug” 6. If the delivery options for Gabrielle are presented, you should ask questions such as: “What are the risks to Gabrielle if she has surgery or if she goes into labor now?” or “I want to make sure she’s safe too, can you tell us what this means for her health?” or “If things get dangerous for her, what will you do?” 7. Some potential other comments to bring in:    1. “What does that mean?” in response to medical terms    2. “Why do we have a choice?”    3. “What do you think is best?”    4. “What will he look like?”    5. “Have any babies born this early been ok?” or “grown to be healthy?”    6. “How do we even make this kind of decision?” |
| HISTORY OF PRESENT ILLNESS: Although some of the HPI will be given in the patient’s symptom story, the learners will expand the story during the direct question section. Below, describe the detailed history, usually about the chief concern, which the student must develop in order to make a useful assessment of the problem: | |
| Onset (when; gradual or sudden) | S |
| Setting (what was going on or where was patient when symptoms first noticed?) | At home |
| Duration (how long) | N/A |
| Time relationships (frequency, constant or intermittent) | N/A |
| Location | N/A |
| Radiation | N/A |
| Quality | N/A |
| Amount | N/A |
| Aggravated by what | N/A |
| Relieved by what | N/A |
| Associated with what | N/A |
| Attitude (what does the patient think is the problem, and how do they feel about it) |  |
| Overall course |  |
| REVIEW OF SYSTEMS: Significant positives and negatives | |
|  | None |
| Past medical history |  |
| Medication allergies (name and reaction) | None |
| Environmental allergies (name and reaction) | None |
| Illnesses | None |
| Vaccinations | N/A |
| Surgeries | None |
| Accidents/injuries/trauma | None |
| Hospitalization | N/A |
|  |  |
| Inclusive sexual and reproductive history |  |
| Sexual practices  Sexual partners  Protection: Use of safer sex practices  Use of birth control if appropriate  Risk of intimate partner violence | N/A |
| OB/GYN history | Age of onset of menses: N/A  Age of menopause: N/A  Number of pregnancies: 1  Number of live births: 0  Number of miscarriages: 2  Number of abortions: 0 |
| Medications | Prescription/dose/reason: None  Over the counter/dose/reason: None  Herbs/supplements/dose/reason: Prenatal vitamin |
| Immunizations | All up to date |
| Tobacco products:   - Cigarettes - Cigar - Pipe - Chew   E-cigarettes | Never |
| Alcohol   - Beer - Wine - Liquor   Other | None currently while pregnant |
| Drugs   - Weed - Cocaine - Heroin - Meth - IV - Inhalants   Other | Never |
| Diet (describe) | N/A |
| - Exercise (describe) | N/A |
| - List any other important social history or information important to this case |  |
| - Family history |  |
| Mother, father, siblings, grandparents, and other significant findings | Partner (Cameron) has a cousin with trisomy 21 |
|  |  |
| Physical Exam - List exam maneuvers expected for this case and any abnormal findings that SP will simulate. (tenderness, hyper-hypo reflex, rebound, weakness, etc.)  None, no exam to be performed |  |
| PHYSICAL EXAM FINDINGS |  |
| Written in layperson’s terms | N/A |
| General appearance - affect, appearance, position of patient at opening (i.e., sitting, lying down, holding abdomen, etc.) | Patient sitting up in hospital bed in hospital gown |
| Vital signs | N/A |
| Specific findings and affect | N/A |
| 1. Response to certain physical movements | None |
|  |  |
| 1. DIAGNOSIS AND DIFFERENTIAL | N/A |
| 1. Diagnosis with support from positive and negative history and PE findings |  |
| 1. Differential with support from positive and negative history and PE findings |  |
|  |  |
| MANAGEMENT OR DIAGNOSTIC PLAN | N/A |
|  |  |
| PROFESSIONALISM ISSUES OR CHALLENGES | None |
|  |  |
