# Supplementary material for: Development of a Joint-Specialty Simulation-Based Workshop to Optimize Counseling at Extreme Prematurity
Source: MedEdPORTAL. 2026 Jul 29;22:11623. doi: 10.15766/mep_2374-8265.11623 (PMC13415433; doi:10.15766/mep_2374-8265.11623)
Supplement: Supplementary file 1 — Prenatal Counseling Workshop.pptxPrenatal Counseling Case.docxFacilitator Guide.docxPostworkshop Survey.docx [file mep_2374-8265.11623-s001.zip › C. Facilitator Guide.docx]

Appendix C: Facilitator Guide

**Recruit and Prepare Facilitators**

Each session should be facilitated by at least 1 Neonatologist and 1 Maternal Fetal Medicine Specialist, ideally with expertise in ethics, professionalism, and advanced communication techniques. If available, a family faculty advisor adds additional expertise in the parental perspective. Facilitators should receive preparatory coaching, including orientation to the session format and review of facilitation techniques. Each facilitator is assigned specific slides to present but is encouraged to review the entire slide deck beforehand to ensure familiarity with all material and in case one of the facilitators becomes unavailable.

**Simulation Instructions**

This was designed as an in-person simulation with virtual participants, but can be modified depending upon needs and available resources. Possible options include, but are not limited to:

1. Simulation in simulated clinical setting (i.e. sim center or empty patient care room) with professional actors portraying pregnant person and partner.
2. Simulation in mock clinical setting with colleagues portraying pregnant person and partner
3. Simulation in classroom with colleagues portraying pregnant person and partner
4. Any of the above scenarios can be performed with virtual or in person participants

**Location/Date**

Once you have determined your available resources and desired session format, you will need to confirm session location and dates. If participants will be in person, have the learners/SPs participate in the scenario in a separate room so that they can better suspend reality, while observers watch from a separate location. This way, those who are not participating in the simulation can also learn through observation and take part in the discussion and debriefing afterwards. If video recording with remote viewing is not possible, it is fine to have participants take part in the scenario with the SPs in the same room as those observing.

**Prepare Learners & Participants**

Advertise this session in advance to your learners, so that learners can block off the time in their schedules and mentally prepare for the emotionally demanding nature of the activity. During the registration period, ask potential participants if they would be willing to participate in the simulation. While participation in the simulation is encouraged, giving learners the option helps ensure that those who do participate feel psychologically safe and fully prepared to engage, while those less comfortable can still learn through observation. If you have a professional actor or colleague portraying the pregnant person and partner, give them the **Case Development Tool** in advance to familiarize them with the role, including specific questions to ask during the simulation to prompt discussion. Find a time to meet with them prior to the session to orient to the scenario including the focus on joint counseling, communication, and recognition of biases with their goal to respond as concerned parents with emotional, practical, and values-based questions. Encourage SPs to use suggested quotes as prompts, weaving them into the conversation rather than reciting them verbatim. If able, use the pilot workshop as an opportunity for the SPs to rehearse the scenario.

Whenever possible, we recommend engaging trained SPs to portray the pregnant person and partner. SPs provide consistency across sessions, ensuring that all learners encounter a similar scenario and have equitable opportunities to practice communication skills. SPs also bring expertise in maintaining realism, delivering authentic emotional responses, and offering structured feedback to learners. We recognize, however, that not all programs have access to professional SPs due to financial or logistical constraints. In such cases, faculty members or colleagues can role-play these roles effectively, particularly if they are given clear scripts, prompts, and rehearsal time.

**Props**

Props can help engage learners by enhancing reality. However, the use of props depends on resources and setting. Possible props include a maternal fetal heart tracing belt from L&D, monitor, pillow (to simulate a pregnant belly), hospital gown, sheet and a hospital bed for the pregnant woman.

**Pilot Workshop**

If time and funding allow, we recommend conducting a small pilot workshop prior to the scheduled session. A pilot provides the opportunity to rehearse the flow of the simulation, identify logistical challenges, and refine the standardized patient prompts and facilitator roles. Feedback from pilot participants can highlight areas of confusion, reveal unanticipated learner questions, and ensure that the session objectives are met. Running a pilot also enhances facilitator confidence and helps create a smoother, more effective learning experience during the full workshop.

**Session**

At the start of the session, establish psychological safety by emphasizing that the activity will take place in a confidential, respectful, and supportive environment. Drawing on the Center for Medical Simulation’s 'fiction contract,' assure your learners that they are well prepared, intelligent, and want to do well. Remind them to suspend reality and remain in their roles during the simulation. Learners should be given background information on the patient, including medical and obstetrical history, estimated fetal weight and sex, as well as some social history, prior to participation in the simulation. The simulation should be limited to 15-20 minutes to allow adequate time for debriefing.

**Suggested Agenda/Time Frame**

Welcome and Introduction (15 min)

Interactive Didactics Part 1 (45 min)

Break (5 min)

Simulation and Debriefing #1 (1 hour)

Break (5 min)

Interactive Didactics Part 2 (35 min)

Break (5 min)

Simulation and Debriefing #2 (1 hour)

Summary, Closure, and Evaluations (10 min)

**Debriefing**

Debrief with your learners after the simulation, including the actors or colleagues who portrayed the pregnant person and partner. Use this time to reflect on the scenario, discuss emotions, analyze communication strategies, and connect observed behaviors to best practices. Including the actors’ perspectives offers authentic feedback on how the interaction felt from a patient or family standpoint, helping learners deepen empathy and refine their skills. Remind learners that this is a safe, confidential and supportive environment.

**Evaluate**

Thank learners and actors/participants for their participation. Ensure learners complete the evaluation form at the end.
