# Supplementary material for: Development of a Joint-Specialty Simulation-Based Workshop to Optimize Counseling at Extreme Prematurity
Source: MedEdPORTAL. 2026 Jul 29;22:11623. doi: 10.15766/mep_2374-8265.11623 (PMC13415433; doi:10.15766/mep_2374-8265.11623)
Supplement: Supplementary file 1 — Prenatal Counseling Workshop.pptxPrenatal Counseling Case.docxFacilitator Guide.docxPostworkshop Survey.docx [file mep_2374-8265.11623-s001.zip › D. Postworkshop Survey.docx]

Appendix D- Postworkshop Survey

**What is your clinical role?**

◦ Physician (Attending)

◦ Physician (Fellow)

◦ Resident

◦ Respiratory Therapist

◦ Nurse Practitioner

◦ Physician Assistant

◦ Registered Nurse

◦ Pharmacist

◦ Social Worker

◦ Physical or Occupational Therapist

◦ Psychologist

◦ Child Life Specialist

◦ Technician/Technologist

◦ Clinical Assistant

◦ Student

◦ Other

**How many years have you been in clinical practice (after receiving your clinical degree)?**

◦ 0 to 2

◦ 3 to 5

◦ 6 to 10

◦ 11 to 15

◦ 16 to 20

◦ 21 or more

**Please indicate the extent to which you agree or disagree with the following statements about this simulation.**

**The learning objective(s) were clearly stated.**

◦ Strongly disagree

◦ Disagree

◦ Neither agree nor disagree

◦ Agree

◦ Strongly agree

**This course met the targeted learning objectives.**

◦ Strongly disagree

◦ Disagree

◦ Neither agree nor disagree

◦ Agree

◦ Strongly agree

**I felt engaged as an active participant in this simulation.**

◦ Strongly disagree

◦ Disagree

◦ Neither agree nor disagree

◦ Agree

◦ Strongly agree

**During simulation, I felt comfortable trying new things.**

◦ Strongly disagree

◦ Disagree

◦ Neither agree nor disagree

◦ Agree

◦ Strongly agree

**The course facilitator clearly explained the concept of psychological safety.**

◦ Strongly disagree

◦ Disagree

◦ Neither agree nor disagree

◦ Agree

◦ Strongly agree

**During debriefing, I felt comfortable sharing openly.**

◦ Strongly disagree

◦ Disagree

◦ Neither agree nor disagree

◦ Agree

◦ Strongly agree

**Debriefing provided opportunities to self-reflect on my performance during simulation.**

◦ Strongly disagree

◦ Disagree

◦ Neither agree nor disagree

◦ Agree

◦ Strongly agree

**Please rate the contribution of the simulation environment to your learning experience.**

◦ Poor

◦ Fair

◦ Average

◦ Good

◦ Excellent

**Indicate the extent to which this training session has changed your preparedness to have difficult conversations with families.**

◦ I feel much less prepared

◦ I feel less prepared

◦ I feel the same as before

◦ I feel more prepared

◦ I feel much more prepared

**Indicate the extent to which this training has changed your preparedness to manage your emotions during difficult conversations with patients and their families.**

◦ I am much less prepared

◦ I am less prepared

◦ I feel the same as before

◦ I am more prepared

◦ I am much more prepared

**Indicate the extent to which this training has changed your preparedness to encourage patients and families to talk about their emotions.**

◦ I am much less prepared

◦ I am less prepared

◦ I feel the same as before

◦ I am more prepared

◦ I am much more prepared

**Please describe any particular types or elements of conversations with patients and families that you find especially difficult.**

**How effective were the following elements in promoting a positive learning experience?**

**Was the group the right size to facilitate your learning?**

▪ Yes

▪ No - too large

▪ No - too small

**We're interested in areas of strength (plus) and areas of improvement (delta) for this simulation learning experience.**

**Plus: Which element(s) of this training most supported your learning? ___________________________________________________________________________**

**Delta: Which element(s) of this training could be improved to better support your learning? __________________________________________________________________________**

**What changes, if any, will you make to your practice as a result of this training? __________________________________________________________________________**
