# Supplementary material for: Development of a Joint-Specialty Simulation-Based Workshop to Optimize Counseling at Extreme Prematurity
Source: MedEdPORTAL. 2026 Jul 29;22:11623. doi: 10.15766/mep_2374-8265.11623 (PMC13415433; doi:10.15766/mep_2374-8265.11623)
Supplement: Supplementary file 1 — Prenatal Counseling Workshop.pptxPrenatal Counseling Case.docxFacilitator Guide.docxPostworkshop Survey.docx [file mep_2374-8265.11623-s001.zip › _Educational Summary Report_11623.pdf]

MedEdPORTAL
THE AAMC JOURNAL OF TEACHING AND LEARNING RESOURCES

Original Publication

Open Access

Development of a Joint-Specialty Simulation-Based Workshop to Optimize Counseling at Extreme Prematurity

Anne Sullivan, MD*, Stephen D. Brown, MD, Erin Ward, MEd, David Williams, PhD, Donna Luff, PhD, Cassandra R. Duffy, MD, MPH, Yael Hoffman Sage, MD, MPH, Melissa Spiel, DO, Christy Cummings, MD

*Corresponding author: anne.sullivan@childrens.harvard.edu

Abstract

Introduction: Educational interventions addressing counseling at extreme prematurity to date have focused on single-specialty clinicians, overlooking the important communication and collaboration required by maternal-fetal medicine and neonatology for optimal counseling. This simulation-based workshop aimed to unite maternal-fetal and neonatology clinicians to enhance interspecialty-personalized and family-focused counseling at extreme prematurity. **Methods:** We developed and conducted 3 half-day virtual workshops with neonatology and maternal-fetal medicine attendings and fellows utilizing a combination of didactics, scenarios with simulated participants, and facilitated debriefs, including a cofacilitator with lived patient-family neonatal intensive care unit experience. **Results:** A total of 26 clinicians participated in the 3 workshops. After the workshop, 92% of participants felt “more prepared” or “much more prepared” to have difficult conversations with families. Qualitative analysis of open-ended responses found key themes among participants, including increased adoption of parent-driven and personalized counseling approaches, enhanced interspecialty collaboration between maternal-fetal medicine and neonatology, and greater awareness of and efforts to mitigate bias in antenatal counseling. **Discussion:** This novel workshop facilitated interspecialty collaboration between maternal-fetal medicine and neonatology when counseling at extreme prematurity and emphasized relational skills, personalized counseling, and bias mitigation techniques.

Keywords
Neonatal-Perinatal Medicine, OB/GYN, Maternal & Fetal Medicine

Educational Objectives

By the end of this session, learners should be able to:

1. Recognize the importance of effective interspecialty communication and collaboration during prenatal counseling, by applying newly acquired communication techniques and relational skills.
2. Apply bias mitigation techniques to counseling expectant parents at extreme prematurity.
3. Demonstrate communication strategies that reflect and incorporate the values, preferences, and perspective of families during counseling of expectant parents at extreme prematurity.
4. Incorporate evidence-based advice from parents when counseling expectant parents at extreme prematurity.

Citation:
Sullivan A, Brown SD, Ward E, et al. Development of a joint-specialty simulation-based workshop to optimize counseling at extreme prematurity. *MedEdPORTAL*. 2026;22:11623.
https://doi.org/10.15766/mep_2374-8265.11623

Introduction

Both neonatology and maternal-fetal medicine (MFM) specialists counsel families at risk of extremely preterm birth, amidst uncertain, rapidly evolving, and emotionally intense circumstances. Optimal counseling may be promoted through collaborative interspecialty discussions prior to family meetings when possible.[1,2] However, engaging in interspecialty conversations can be difficult to implement without adequate training or exposure to such collaborative models. Educational opportunities for clinical counseling at extreme prematurity are rare, often isolated within specialties, and may not reflect best-practice recommendations for counseling.[3,4] Despite recommendations from professional societies encouraging collaborative interspecialty prenatal counseling, MFM and neonatal specialists often counsel families asynchronously, resulting in potentially conflicting messages to families, which may increase stress and decision-making burden.[1,5-7]

Copyright © 2026 Sullivan et al. This is an open-access publication distributed under the terms of the Creative Commons Attribution license.

Another barrier to effective counseling is clinician bias, which can impact how information is presented, the options emphasized, and the recommendations made. These biases can relate to demographics, such as race, ethnicity, socioeconomic status, religion, language, or disability, as well as biases in clinical recommendations, such as favoring resuscitation or nonresuscitation based on personal beliefs rather than strictly on medical evidence and the family's stated values.[6,8,9] Bias, whether conscious or unconscious, can shape how prognosis is framed, how risks and benefits are presented, and how decision-making conversations unfold.[10,11]

Finally, an essential component of high-quality counseling at extreme prematurity is using language and communication strategies that reflect the family's values, preferences, and lived experience.[4,6,12-14] Families facing the possibility of an extremely premature delivery often navigate overwhelming uncertainty, and the way information is conveyed can shape both their understanding and their sense of partnership in decision-making. Training that explicitly emphasizes respectful, family-centered language helps clinicians avoid jargon, acknowledge potential biases, and create space for the perspectives of both the pregnant person and their partner to guide discussions about resuscitation, maternal health, and goals of care.[4]

To date, no curricula or detailed educational frameworks have been published to inform how best to educate MFM and neonatology clinicians together toward promoting optimal joint counseling practice. Existing educational interventions primarily focus on clinicians within a single specialty,[15,16] often overlooking the crucial communication and collaboration between MFM and neonatology that would likely optimize counseling expectant parents at extreme prematurity.

Despite the recognition that joint counseling improves parental understanding and decision-making, there remains no standardized educational framework to train clinicians in an interspecialty approach. Relational learning models show that effective learning can occur not only through direct participation in conversations but also by observing others and engaging in shared experiential learning with interspecialty colleagues.[17-19] Simulation and enacted role-play using relational learning have been shown to be effective in teaching prenatal counseling for neonatologists.[4,17,18] This framework supports learners in moving beyond scripted communication toward adaptive, authentic dialogue with families. The overall goal of the workshop was to develop and implement a novel, simulation-based interspecialty workshop to unite MFM and neonatology clinicians, by focusing on eliciting values and building partnerships through advanced communication and relational skills, to improve personalized counseling at extreme prematurity.

Methods

Development of the Workshop

The workshop was developed collaboratively with the Boston Children's Hospital's Immersive Design Systems Program (IDS). The half-day workshops incorporated validated relational learning methodology and addressed educational challenges specific to neonatology and MFM when counseling at extreme prematurity. Participants included fellows and attending physicians from both MFM and neonatology involved with counseling expectant families at extreme prematurity at 3 different local institutions. Participants were recruited on a voluntary basis via email. This workshop is part of a larger behavioral intervention study registered with ClinicalTrials.gov (NCT03819933). The study was approved by the Boston Children's Hospital Institutional Review Board (IRB), which acted as a single, reliance IRB for all participating sites (IRB-P00030146).

The workshops were held remotely via Zoom. The workshop consisted of evidence-based didactic presentations coupled with 2 scenarios conducted in-person with simulated participants (SPs), who were professionally trained actors, whereas course participants joined and engaged virtually. Didactic presentations emphasized findings from prior mixed methods work surrounding counseling at extreme prematurity, including compassionately engaging and communicating with families, aligning messaging, and strategies to mitigate bias when counseling[6,20] (Appendix A). Didactic presentations were divided into 2 segments, with each segment followed by a scenario. The goal was to allow participants to apply key learning points from the preceding didactic to the scenario. Each scenario was followed by a facilitated group debriefing, during which participants who took part in the scenario received feedback from course faculty, the SPs, and the other course participants.

The workshops employed a 2-part case scenario (Appendix B), involving SPs portraying a pregnant person admitted with threatened preterm delivery at 23 weeks of gestational age, and the partner, for whom antenatal consultations (MFM and neonatology) have been requested. Appendix B outlines the case and SP character information in detail.

The family faculty advisor (Erin Ward), a former neonatal intensive care unit (NICU) parent with additional expertise in communication skills, participated in the development and facilitation of the workshop, the questionnaire, and the facilitator guide to incorporate the family perspective. After each workshop,

Copyright © 2026 Sullivan et al. This is an open-access publication distributed under the terms of the Creative Commons Attribution license.

all participants received ∃100 gift cards to recognize their time and effort.

Facilitators

Each workshop was facilitated by 1–2 neonatologists; 1 MFM specialist; 1 physician specializing in ethics, professionalism, and health care communication; and 1 family faculty advisor. Appendix C summarizes instructions for recruiting and preparing facilitators for the session. Facilitators were invited to participate based on prior training and expertise in challenging conversations and antenatal counseling. We also conducted a 1-hour facilitator training session to review workshop content and roles during didactics and debriefs (see Appendix C). Each facilitator was assigned different segments of the didactic to present. All were familiar with the full content to allow for role flexibility and redundancy.

Workshop Implementation

SPs were recruited by IDS and received written role preparation materials for their roles in advance, with an in-depth case scenario, character information, and trigger comments/questions for the SP to use during the scenario (see Appendix B). SPs also had an opportunity to prebrief with the course family faculty advisor to ensure the SPs' understanding of the scenario and preparation was informed by authentic lived family experiences. Facilitators met with SPs for 30 minutes before workshops to review goals, actor worksheets, and answer questions.

A pilot workshop was performed with the workshop facilitators and SPs 2 weeks prior to full workshop implementation to refine and optimize the session. During this session, the facilitators practiced the didactic presentations and participated in the scenario, allowing the SPs to rehearse their roles. This was followed by a debriefing where opportunities for improvement were discussed.

Three 4-hour workshops were held. SPs and some facilitators were physically present in the simulation center, whereas all other participants, including those directly participating in the scenarios, joined remotely. To promote active engagement, all participants were asked to keep their videos on during the entire workshop, except for periodic breaks and enactments when they were not directly involved.

The SPs, portraying the pregnant person and partner, were present in person in our simulation laboratory, a labor and delivery triage room. The pregnant person was wearing a hospital gown with an artificial pregnant abdomen underneath. Her partner was seated in the chair next to her, or for one of the workshops, virtually via Zoom. A tablet with video capabilities on a rolling cart was positioned so the participants could interact with the SPs in the virtual platform. One MFM and 1 neonatologist participated in each scenario with the remainder of the participants watching. Participating physicians were asked to perform a precounseling huddle together on Zoom prior to meeting the SPs and then jointly counsel the pregnant person and partner. This was followed by a group debriefing with all workshop attendees, SPs, and facilitators.

To enhance reproducibility, we provide workshop slides, actor scripts, a facilitator guide, and evaluation (see Appendices A-D). Institutions with limited simulation resources can adapt the workshop using locally available technology.

Evaluation

Participants evaluated the workshop via a questionnaire that used a combination of Likert-scale and open-ended questions developed by IDS for simulation scenarios for advanced communication techniques (Appendix D). Two independent researchers coded open-ended responses to ensure reliability, and discrepancies were resolved through discussion.

Results

Demographics and Likert Responses

A total of 26 clinicians participated in the 3 separate workshops, including 23 attending physicians (9 MFM/obstetrics and 14 neonatology) and 3 fellows (2 MFM/obstetrics and 1 neonatology), with 8-9 participants per workshop. Participants included clinicians at varying career stages, with more than half having greater than 10 years of clinical practice (Table 1). The postworkshop questionnaire was completed by 96% of participants. After completion of the workshop, 96% of the respondents reported feeling engaged as an active participant in the simulation/workshop, with 92% of respondents rating the contribution of the simulation environment to their learning

Table 1. Participant Demographics

Item	Frequency (%)
Role	
Neonatology attending	14 (53.8)
Neonatology fellow	1 (3.8)
MFM attending	9 (34.6)
MFM fellow	2 (7.7)
Years of Experience	
0-2	1 (3.8)
3-5	4 (15.4)
6-10	7 (26.9)
11-15	5 (19.2)
16-20	4 (15.4)
>21	5 (19.2)

Abbreviation: MFM, maternal-fetal medicine.

Copyright © 2026 Sullivan et al. This is an open-access publication distributed under the terms of the Creative Commons Attribution license.

experience as “excellent” or “good.” Ninety-two percent of respondents felt “more prepared” or “much more prepared” to have difficult conversations with families, and 84% of respondents felt “more prepared” or “much more prepared” to manage their emotions during difficult conversations with families.

Qualitative Analysis

Qualitative content analysis of open-ended questions identified several unique themes. When asked to describe elements of conversation that they found particularly difficult, a few compelling themes emerged (Table 2). Respondents shared prior difficulties performing nonbiased counseling, sufficiently assessing and getting to know the family to effectively communicate, framing options, communicating uncertainty, and optimizing time management. Respondents also shared a few specific challenging discussion points, including termination of pregnancy, religion, race, and impacts of prematurity.

Many respondents reported the value of the simulation in supporting their learning, particularly the quality of the SPs and realism of the scenario. One participant wrote, “Great actors—they brought their individual humanity—didn’t seem like acting.” Respondents also found the debriefs important and highlighted the value in hearing the SP’s perceptions of the counseling and appreciated the family faculty advisor’s perspective as well. Similarly, respondents valued the quality of the didactic sessions, particularly the inclusion of novel research findings and concrete examples of language to use during the consult.

When prompted, respondents also suggested ways to improve specific elements of the workshop. Many commented on the limitations of having only a few participants participate in the simulated scenario; some suggested shorter simulations or more scenarios to involve more clinicians. Some respondents preferred shorter didactics to maximize time allowed for the simulation and debrief. Many also commented on the limitations of the virtual simulation specifically with respect to participation and picking up on nonverbal communication. While virtual participation was more convenient, some felt an in-person simulation may have improved learning.

Respondents shared concrete changes they planned to make as a result of the training (Table 3). Most commonly, respondents described shifting to a more parent-driven, personalized approach in their counseling. One participant wrote, “Affirms changes in progress to approach being more parent-driven and parent focused and less about my agenda.” Respondents also expressed a desire to engage in more collaborative counseling with both MFM and neonatology, particularly through a preconsult huddle. Other common themes included respondents’ desire to provide less biased counseling, modify their language, tailor the amount of information in the consultation, and be conscious of the pace of the conversation.

Discussion

We developed and implemented a novel interspecialty joint MFM and neonatology simulation-based workshop to improve antenatal counseling, emphasizing relational skills, personalized counseling, and bias mitigation techniques. After engaging in this remotely conducted workshop, our survey results indicated that participants felt more prepared to perform these consultations in the future and collaborate across specialties, and were more

Table 2. Elements of Antenatal Counseling Conversations That Participants Found Challenging

Theme	Example Participant Responses
Getting to know family	• “Assessing the families hopes and dreams”<br>• “Difficult to find words to get to the patients values and biases and goals”
Optimal framing of options	• “The best way to frame all options available, in the context of parental values”<br>• “Making sure I give them all the possible outcomes and be able to talk about potential long-term outcomes in a way that can inform their decision”<br>• “Introducing the option of comfort care and how that can align with a family’s preferences”
Sitting with uncertainty	• “Describing the uncertainty about future developmental outcomes”<br>• “Uncertainty of preferences/beliefs/outcomes/clinical paths”
Disparate family values/perspectives	• “Conflicting religious frameworks”<br>• “Multiple parent perspectives”<br>• “When parents have disparate goals”
Time needed to have discussions	• “Time management! I feel comfortable digging into the feelings but find it hard to get to decision making quickly”<br>• “Time pressure”
Specific difficult discussion points	• Termination<br>• Religion<br>• Race<br>• Possible infant’s death<br>• Long-term effects

Copyright © 2026 Sullivan et al. This is an open-access publication distributed under the terms of the Creative Commons Attribution license.

Table 3. Changes As a Result of the Workshop

Change Theme	Example Participant Responses
More parent-driven/personalized approach	• "I will be more supportive for the parents"<br>• "Incorporating the advice in terms of connecting"<br>• "Focus on parental thinking"<br>• "Try to specifically elicit family's values and preferences"
More collaborative approach with MFM and NICU	• "I will do a pre-consult huddle with MFM and NICU teams"<br>• "Collaborative approach to counseling with MFM and NICU"<br>• "Increase huddling with NICU and MFM together"<br>• "Prioritize joint counseling between OB and MFM"
Appreciate impact of bias	• "Incorporate bias mitigation techniques"<br>• "More comprehensive and less biased counseling"
Modify language	• "Got some great phrases"<br>• "I learned some good language for counseling in the setting of extreme prematurity"<br>• "I would prepare for specific terms for counseling"<br>• "Using certain language (or avoiding others)"
Tailor amount of information	• "Not feel bound to give all the medical data that I think pertinent—at least not all at once"<br>• "It's ok not to be all-encompassing - timing is key and certain issues can be addressed at a later time-point"<br>• "Cementing the relationship before presenting information"
Consciousness of the pace of the conversation	• "Be more conscious about the pace of the conversation (in the context of the urgency of the situation)"<br>• "Slow down the conversation"

Abbreviations: MFM, maternal-fetal medicine; NICU, neonatal intensive care unit; OB, obstetrics.

likely to be family-centered and nonbiased in their approach. Participant feedback was overwhelmingly positive, with free text responses highlighting all elements of the workshop, including the didactics, simulation, and debrief.

Of note, many communication elements that participants found difficult were emphasized during this workshop, including understanding the family perspective, mitigating bias, and empathetically discussing treatment options. This underscores the great need for this type of continuing education, not just for trainees but also for practicing clinicians, as more than half of the participants in our workshop had more than 10 years of experience. Furthermore, while many prior workshops published in the literature focus on single specialty counseling, our workshop demonstrates the positive impact of uniting both specialties to facilitate a more collaborative educational approach to the counseling process.

This workshop was designed during the COVID-19 pandemic, which informed the decision to make all participants remote. The main advantage of a remotely held workshop is the ability for multisite collaboration and the potential to increase participation. For clinicians with busy schedules, the virtual option allows for flexibility and participation without need for commute time. However, there are also potential disadvantages to the virtual workshop that were reflected in some of the evaluations. The suspension of disbelief during an antenatal consultation over Zoom was difficult for some participants, who commented that an in-person simulation might have been more realistic. In addition, in our postpandemic era, the prevalence of multitasking on virtual platforms may have contributed to reduced participant engagement. It is possible, however, that if the workshop had been offered in person, enrollment may have been lower.

A limitation of this workshop was that only a small subset of participants could directly participate in simulated case scenarios, whereas the remainder served as observers. However, significant participant learning can occur from modeling and observing,[19,21] and the facilitated debrief aimed to include reflections from all participants to ensure engagement. Future implementations could more deliberately assess whether learning outcomes differ between participants who engage directly with the SPs and those who observe. Evaluating role-specific outcomes would provide important insights into how to best balance active participation and observation in communication-based simulation. Other limitations that may impede generalizability include variable availability of simulation resources at home institutions. As participants were primarily from academic centers, findings may not be generalizable to community-based or resource-limited settings.

However, by providing a robust actor worksheet and case scenario, this may be overcome through alternative approaches and available resources. In addition, given the small sample

Copyright © 2026 Sullivan et al. This is an open-access publication distributed under the terms of the Creative Commons Attribution license.

size ($N = 26$) and the predominance of attending physicians with more than 10 years of experience, findings may not be fully generalizable to trainees or early-career clinicians. Future studies should explore the impact of this workshop on a broader, multi-institutional cohort. The lack of longitudinal evaluation to assess if the participants' practice changed because of the workshop also remains a limitation in our ability to assess the long-term impact of the workshop and should be considered in future iterations. Finally, while virtual workshops allow for multi-institutional participation and greater accessibility, some participants reported challenges in picking up nonverbal cues. Future iterations could explore hybrid models that incorporate both in-person and virtual elements to optimize engagement.

Finally, this workshop incorporated the perspective of only 1 former NICU family, who had chosen to pursue resuscitation. As such, the family narrative may not reflect the range of decisions or experiences at the threshold of viability. Including families who have chosen comfort or termination of pregnancy could provide a more balanced perspective. Using the same family in both scenarios allowed participants to observe the evolution of counseling over time; however, this approach may have introduced bias toward that family's decision. Future iterations could include 2 different families with differing resuscitation preferences to expose learners to a broader range of decision-making processes.

This workshop represents a scalable, evidence-based approach to interspecialty training in extreme prematurity counseling. The integration of simulation, debriefing, and SP feedback provides a framework adaptable across settings, including community hospitals and international training programs. Future studies should assess long-term skill retention and clinical practice change, including employing interspecialty communication techniques, bias awareness, and incorporating the family perspective, as well as explore parental perspectives on counseling after such training. This workshop aligns with competency-based medical education frameworks, particularly in communication, professionalism, and systems-based practice. Future directions include trialing the workshop in person and measuring direct counseling outcomes resulting from this intervention (in progress).

Appendices

A. Prenatal Counseling Workshop.pptx

B. Prenatal Counseling Case.docx

C. Facilitator Guide.docx

D. Postworkshop Survey.docx

All appendices are peer reviewed as integral parts of the Original Publication.

Anne Sullivan, MD: Assistant Professor, Division of Newborn Medicine, Boston Children's Hospital; ORCID: https://orcid.org/0000-0002-0598-4964

Stephen D. Brown, MD: Associate Professor and Associate Clinical Ethicist, Department of Radiology, Boston Children's Hospital

Erin Ward, MEd: Patient Engagement Consultant, Division of General Pediatrics, Boston Children's Hospital

David Williams, PhD: Senior Biostatistician, Institutional Centers for Clinical and Translational Research, Boston Children's Hospital

Donna Luff, PhD: Director for Center for Educational Excellence and Innovation Division of Anesthesia, Boston Children's Hospital

Cassandra R. Duffy, MD, MPH: Assistant Professor, Division of Maternal-Fetal Medicine, Beth Israel Deaconess Medical Center

Yael Hoffman Sage, MD, MPH: Assistant Professor, Department of Maternal-Fetal Medicine, Brigham and Women's Hospital

Melissa Spiel, DO: Assistant Professor, Division of Maternal-Fetal Medicine, Beth Israel Deaconess Medical Center

Christy Cummings, MD: Associate Professor and Clinical Ethicist, Division of Newborn Medicine, Boston Children's Hospital

Acknowledgments
The authors would like to extend their sincere gratitude to the professional actors, Thomika Marie Bridwell and JaQuan Malik Jones, for their invaluable contributions to the workshop. Their skill and dedication brought authenticity and depth to the simulation experience. We also wish to thank the entire team at Immersive Design Systems for their support in executing this workshop. We acknowledge Anne Fox, Cory Gill, Sydney Manning, Srijita Sinha, and Saja Traoui, whose expertise and hard work were instrumental in ensuring the success of the session.

Disclosures
None to report.

Funding/Support
Dr. Christy Cummings received funding from the National Institute for Child Health and Human Development, grant number R01 HD094794.

Ethical Approval
Boston Children's Hospital Institutional Review Board reviewed this project.

Copyright © 2026 Sullivan et al. This is an open-access publication distributed under the terms of the Creative Commons Attribution license.

References

1. Cummings J, Committee on Fetus and Newborn, Watterberg K, et al. Antenatal counseling regarding resuscitation and intensive care before 25 weeks of gestation. *Pediatrics*. 2015;136(3):588-595. https://doi.org/10.1542/peds.2015-2336
2. Sullivan A, Donovan B, Young BC, Cummings C. Collaboration between maternal-fetal medicine and neonatology when counseling at extreme prematurity. *Neoreviews*. 2023;24(3): e137-e143. https://doi.org/10.1542/neo.24-3-e137
3. Arzuaga BH, Cummings CL. Practices and education surrounding anticipated periviable deliveries among neonatal-perinatal medicine and maternal-fetal medicine fellowship programs. *J Perinatol*. 2016;36(9):699-703. https://doi.org/10.1038/jp.2016.68
4. Stokes TA, Watson KL, Boss RD. Teaching antenatal counseling skills to neonatal providers. *Semin Perinatol*. 2014;38(1):47-51. https://doi.org/10.1053/j.semperi.2013.07.008
5. Tucker Edmonds B, Mckenzie F, Panoch JE, Barnato AE, Frankel RM. Comparing obstetricians' and neonatologists' approaches to periviable counseling. *J Perinatol*. 2015;35(5):344-348. https://doi.org/10.1038/jp.2014.213
6. Sullivan A, Arzuaga B, Luff D, et al. A qualitative study of parental perspectives on prenatal counseling at extreme prematurity. *J Pediatr*. 2022;251:17-23.e2. https://doi.org/10.1016/j.jpeds.2022.09.003
7. Reed R, Grossman T, Askin G, Gerber LM, Kasdorf E. Joint periviability counseling between neonatology and obstetrics is a rare occurrence. *J Perinatol*. 2020;40(12):1789-1796. https://doi.org/10.1038/s41372-020-00796-8
8. McKenzie F, Robinson BK, Tucker Edmonds B. Do maternal characteristics influence maternal-fetal medicine physicians' willingness to intervene when managing periviable deliveries? *J Perinatol*. 2016;36(7):522-528. https://doi.org/10.1038/jp.2016.15
9. Kunkel MD, Downs SM, Tucker Edmonds B. Influence of maternal factors in neonatologists' counseling for periviable pregnancies. *Am J Perinatol*. 2017;34(8):787-794. https://doi.org/10.1055/s-0037-1598247
10. Haward MF, Murphy RO, Lorenz JM. Message framing and perinatal decisions. *Pediatrics*. 2008;122(1):109-118. https://doi.org/10.1542/peds.2007-0620
11. Shapiro N, Wachtel EV, Bailey SM, Espiritu MM. Implicit physician biases in periviability counseling. *J Pediatr*. 2018;197: 109-115.e1. https://doi.org/10.1016/j.jpeds.2018.01.070
12. Boss RD, Hutton N, Sulpar LJ, West AM, Donohue PK. Values parents apply to decision-making regarding delivery room resuscitation for high-risk newborns. *Pediatrics*. 2008;122(3):583-589. https://doi.org/10.1542/peds.2007-1972
13. Kharrat A, Moore GP, Beckett S, Nicholls SG, Sampson M, Daboval T. Antenatal consultations at extreme prematurity: a systematic review of parent communication needs. *J Pediatr*. 2018;196:109-115.e7. https://doi.org/10.1016/j.jpeds.2017.10.067
14. Staub K, Baardsnes J, Hébert N, Hébert M, Newell S, Pearce R. Our child is not just a gestational age. A first-hand account of what parents want and need to know before premature birth. *Acta Paediatr*. 2014;103(10):1035-1038. https://doi.org/10.1111/apa.12716
15. Janice-Woods Reed D, Sharma J. Delivering difficult news and improving family communication: simulation for neonatal-perinatal fellows. *MedEdPORTAL*. 2016;12:10467. https://doi.org/10.15766/mep_2374-8265.10467
16. Olson DN, Brandt A, Greywitt S, Gibson KS. A multidisciplinary standardized patient simulation for using trauma-informed care for pregnant patients. *MedEdPORTAL*. 2024;20:11474. https://doi.org/10.15766/mep_2374-8265.11474
17. Lamiani G, Meyer EC, Browning DM, Brodsky D, Todres ID. Analysis of enacted difficult conversations in neonatal intensive care. *J Perinatol*. 2009;29(4):310-316. https://doi.org/10.1038/jp.2008.228
18. Meyer EC, Brodsky D, Hansen AR, Lamiani G, Sellers DE, Browning DM. An interdisciplinary, family-focused approach to relational learning in neonatal intensive care. *J Perinatol*. 2011;31(3):212-219. https://doi.org/10.1038/jp.2010.109
19. Bell SK, Pascucci R, Fancy K, Coleman K, Zurakowski D, Meyer EC. The educational value of improvisational actors to teach communication and relational skills: perspectives of interprofessional learners, faculty, and actors. *Patient Educ Couns*. 2014;96(3):381-388. https://doi.org/10.1016/j.pec.2014.07.001
20. Sullivan A, Arzuaga B, Luff D, Ward E, Williams DN, Cummings C. Advice to clinicians from expectant parents at extreme prematurity: a multimethod study. *Pediatrics*. 2024;153(3): e2023062178. https://doi.org/10.1542/peds.2023-062178
21. Stegmann K, Pilz F, Siebeck M, Fischer F. Vicarious learning during simulations: is it more effective than hands-on training? *Med Educ*. 2012;46(10):1001-1008. https://doi.org/10.1111/j.1365-2923.2012.04344.x

Received: May 11, 2026
Accepted: June 18, 2026
Published: July 29, 2026

Copyright © 2026 Sullivan et al. This is an open-access publication distributed under the terms of the Creative Commons Attribution license.
